# Temporal Patterns of Outcomes After DMARD Tapering or Withdrawal in Rheumatoid Arthritis With Sustained Disease Control: A Systematic Review and Meta‐Analysis

**DOI:** 10.1111/cts.70740

**Published:** 2026-10-06

**Authors:** Xiaoyan Zhang, Rajan Kumar Pandey, Xi Luo, Zhongwei Xu

**Affiliations:** ^1^ Department of Anesthesiology, West China Hospital Sichuan University Chengdu China; ^2^ West China School of Nursing Sichuan University Chengdu China; ^3^ Medical Inflammation Research, Department of Medical Biochemistry and Biophysics Karolinska Institute Stockholm Sweden; ^4^ Department of Orthopedic Surgery The First Affiliated Hospital of Chongqing Medical University Chongqing China

**Keywords:** antirheumatic agents, drug tapering, rheumatoid arthritis, symptom flare up

## Abstract

Rheumatoid arthritis (RA) requires lifelong treatment with disease‐modifying antirheumatic drugs (DMARDs). In patients with sustained disease control, DMARD de‐escalation has been proposed to lessen treatment burden, but its efficacy and safety remain elusive. We conducted a systematic review and meta‐analysis of randomized controlled trials evaluating DMARD tapering or withdrawal in controlled RA. PubMed, Embase, and the Cochrane Library were searched to January 16, 2026. The primary outcome was disease flare, and secondary outcomes included radiographic progression and adverse events (AEs). Risk ratios (RRs) were pooled using Mantel–Haenszel models. Prespecified subgroup analyses were conducted by DMARD class and follow‐up duration. In total, 27 publications comprising 5262 participants were included. Any DMARD tapering increased flare risk overall (RR 1.56, 95% CI 1.26–1.98), mainly driven by the biologic DMARD (bDMARD) subgroup, with increased flare risk observed < 9 months (RR 1.61, 95% CI 1.13–2.31), but not at longer follow‐up (> 9 months). In contrast, any DMARD withdrawal substantially increased flare risk (RR 2.23, 95% CI 1.83–2.94), primarily driven by the bDMARD subgroup across all follow‐up durations (< 9 months, 9–18 months, and > 18 months). Both tapering (weak evidence) and withdrawal (strong evidence) promoted radiographic progression at 9–18 months and > 18 months. Neither tapering nor withdrawal reduced AEs. In conclusion, DMARD tapering, particularly of bDMARDs, should be considered cautiously in controlled RA because of the increased flare risk, possible radiographic progression, without clear reduction in AEs. DMARD withdrawal should be generally avoided whenever possible.

**Trial Registration:**

CRD420251066774 (PROSPERO)

## Study Highlights

- What is the current knowledge on the topic?
  ○ DMARD tapering has been advocated to reduce treatment burden, toxicity, and cost, but prior reviews often mixed patients with transient or sustained remission/LDA and pooled outcomes from all follow‐up durations, thereby limiting clinical applicability.
- What question did this study address?
  ○ The study asked whether reducing or withdrawing DMARD therapy in rheumatoid arthritis patients with sustained disease control is effective and safe over time. In particular, it examined whether the increased risk of flare after DMARD tapering or withdrawal is temporary or persists during longer follow‐up, especially for biologic DMARDs.
- What does this study add to our knowledge?
  ○ This study adds that the consequences of DMARD de‐escalation follow distinct temporal patterns. DMARD tapering mainly increases flare risk during the first 9 months, whereas complete withdrawal leads to a persistently elevated flare risk across short‐, medium‐, and long‐term follow‐up, particularly for biologic DMARDs. It also shows that neither strategy reduces adverse events, while both may contribute to radiographic progression over time.
- How might this change clinical pharmacology or translational science?
  ○ This study highlights that DMARD tapering alone does not improve safety within the follow‐up durations, despite increasing the risk of flare and radiographic progression. Since lifelong DMARD exposure may increase cumulative adverse effects, our findings open opportunities for new pharmacologic approaches with lower toxicity to be combined with tapered DMARD, with the goal of maintaining disease control while reducing long‐term DMARD burden.

## 1 Introduction

Rheumatoid arthritis (RA) is a chronic autoimmune disease characterized by persistent synovitis, progressive joint damage, functional disability, and potential systemic complications if inadequately controlled. The introduction of disease‐modifying antirheumatic drugs (DMARDs), including conventional synthetic DMARDs (csDMARDs), biologic DMARDs (bDMARDs), and targeted synthetic DMARDs (tsDMARDs), has revolutionized RA management by achieving sustained remission or low disease activity (LDA) in a significant proportion of patients. These therapeutic advances, particularly when applied within treat‐to‐target strategies [1], have prompted important questions regarding optimal long‐term management for patients who attain sustained disease control.

Although sustained remission or LDA is a desirable therapeutic endpoint, lifelong continuous DMARD therapy confers significant concerns. Chronic immunosuppression exposes patients to various adverse effects including infections and tumors [2, 3, 4], and contributes to significant healthcare costs [5]. As a result, tapering or withdrawing DMARDs has become a key research focus, aiming to determine whether patients with sustained disease control can safely reduce treatment intensity without jeopardizing clinical stability or long‐term outcomes.

In recent years, randomized controlled trials (RCTs) have increasingly evaluated tapering and withdrawal strategies in RA patients with sustained remission or LDA. The majority of these studies show that although a meaningful proportion of patients maintain disease control after dose reduction or discontinuation, flare rates generally remain higher compared with continued therapy [6, 7, 8, 9, 10]. At the same time, several trials have reported that tapering or withdrawal can be feasible and even achieve non‐inferiority in selected patient populations [11, 12, 13]. However, interpretation of individual RCTs remains challenging because of heterogeneity in patient characteristics, tapering strategies, DMARD classes, durations of follow‐up, and varying definitions of “sustained” disease control and flare, etc.

Existing systematic reviews and meta‐analyses have contributed valuable insights into DMARD tapering and withdrawal but still leave important gaps. Early reviews [14, 15, 16, 17] often included studies enrolling patients with sustained or temporary remission/LDA, reducing their clinical applicability. This is notable because major RA management guidelines [18, 19, 20] recommend considering tapering only in patients with sustained remission, not those who have temporarily achieved remission or LDA. Most importantly, prior reviews pooled outcomes irrespective of follow‐up duration, which neglects the time‐dependent effects of tapering or withdrawal and may compromise the validity of the pooled estimates.

Given the heterogeneity of individual RCT findings and the lack of a comprehensive synthesis focused specifically on the temporal patterns of DMARD tapering or withdrawal, a systematic review and meta‐analysis is needed. Such a quantitative synthesis can more precisely estimate the time‐dependent risks of flare, structural progression, and adverse events, etc., further supporting decision‐making for clinicians and patients seeking to balance disease control while minimizing life‐long DMARD exposure.

## 2 Methods

This systematic review and meta‐analysis was registered in PROSPERO (CRD420251066774) and conducted in accordance with the PRISMA 2020 reporting guidelines [21].

### 2.1 Inclusion Criteria

Eligible studies were RCTs that evaluated tapering and/or withdrawal of DMARDs in adults with RA who had achieved sustained remission or LDA for a predefined period before randomization (at least two visits). Studies needed to include patients diagnosed according to established RA classification criteria (1987 or 2010) [22, 23], and investigated tapering or withdrawal strategies involving csDMARDs such as methotrexate, bDMARDs such as tumor necrosis factor inhibitor (TNFi), or tsDMARDs (Janus kinase inhibitor, JAKi). Trials were required to compare tapering or withdrawal with continuation of standard treatment and to report at least one clinically relevant outcome related to disease activity such as flare, relapse, or remission maintenance, etc. To maximize clinical applicability while minimizing heterogeneity attributable to outdated therapeutic strategies and definitions, only full‐length reports published within the past 15 years in peer‐reviewed journals were considered.

### 2.2 Exclusion Criteria

Trials were excluded if they were not randomized controlled studies, including observational studies, registry analyses, case reports, reviews, editorials, or conference abstracts lacking extractable data. Studies enrolling patients without sustained disease control at randomization, those focusing on pediatric populations or conditions other than RA, and those examining glucocorticoid tapering without concomitant DMARD tapering or withdrawal were excluded. Specifically, studies were excluded if patients had active RA at randomization, or if sustained disease control prior to de‐escalation was not clearly established. Studies were also excluded if they lacked an appropriate control group, or proper readout, evaluated dose escalation rather than tapering, or reported interim results. Studies were further excluded if full texts were unavailable or outcome data could not be extracted. When multiple reports arose from the same trial, we used the most recent publication with the most complete dataset. An exception was made when separate publications reported outcomes at different follow‐up durations, and in these cases, we included all time‐distinct reports and, for each predefined analysis window, we extracted data from the report corresponding to that window, ensuring that participants were not double‐counted.

### 2.3 Article Identification

A systematic literature search was conducted in PubMed, MEDLINE/Embase, and Cochrane Library to identify RCTs evaluating tapering or withdrawal of DMARDs in patients with RA who had achieved sustained remission or LDA for a predefined period. The search strategy (Note S1) combined controlled vocabulary terms (MeSH and Emtree) and free‐text keywords related to DMARDs, including csDMARDs, bDMARDs, and tsDMARDs; treatment de‐escalation strategies; rheumatoid arthritis; remission or low disease activity states; and randomized trial design. In Embase, searches were restricted to records indexed as articles or clinical trials. In the Cochrane Library, searches were limited to trials, with records sourced from PubMed or Embase excluded to minimize duplication. Lastly, the reference lists of identified trials and relevant reviews were screened, and additional eligible studies were included. No publication year restrictions were applied at the database search stage, but studies published more than 15 years prior were excluded during post‐search processing by filtering imported records. The final search was conducted on 16 January 2026.

All retrieved records were exported to EndNote (version 20.0.1), where duplicates were removed. Automated filtering using the Smart Group function was applied to identify records containing key terms related to rheumatoid arthritis, tapering interventions, and randomized trial methodology, while excluding studies focused primarily on glucocorticoids. Title and abstract screening were then performed manually. Registry‐based studies were predominantly excluded at this stage, and additional eligible records were identified through searches of trial registration numbers and study names. Full‐text review was conducted independently by two reviewers (X.Z. and Z.X.), with disagreements resolved by discussion or by a third reviewer (R.K.P.). Details of the study selection process are presented in Figure 1 following the PRISMA 2020 flow diagram [21].

**FIGURE 1:**
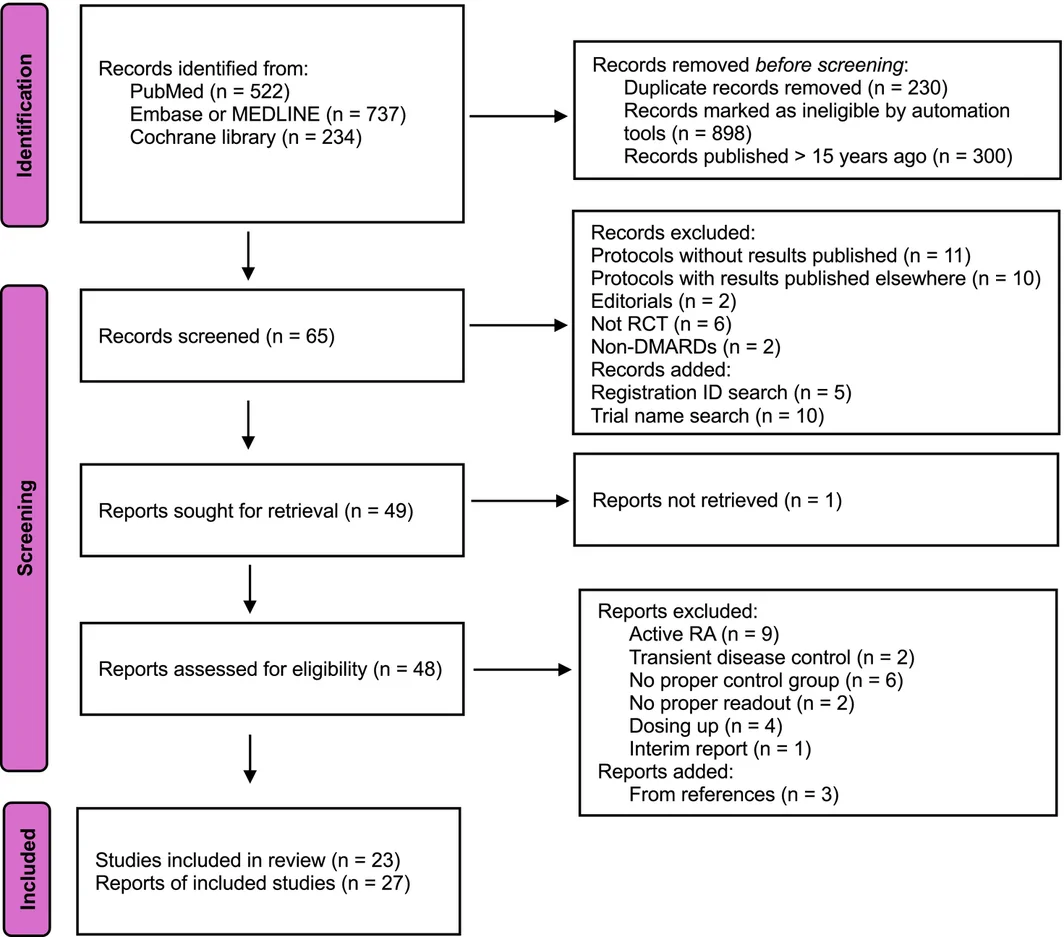
Flow diagram for article search, screening, and identification.

### 2.4 Data Extraction

From each eligible study, basic study‐level and trial characteristics were extracted using a standardized data extraction form. Extracted information included the report identifier (first author, year of publication, and trial name if available), patient eligibility criteria and disease status at randomization (including requirements for sustained remission or LDA), and study grouping and randomization structure. Details of the intervention were recorded, including the type of DMARD therapy, grouping strategy, and the specific tapering or withdrawal approach (e.g., complete withdrawal, stepwise tapering, or dose reduction, etc.). Definitions of flare and/or relapse were extracted as reported by each study, including the disease activity indices and thresholds used. In addition, primary endpoints, duration of follow‐up (in months), and key aspects of study design (e.g., non‐inferiority or superiority framework) were collected.

The primary outcome was disease flare, typically defined using disease activity score (DAS)‐based or other validated algorithmic flare criteria. When flare was not explicitly reported, conceptually similar endpoints such as relapse or loss of remission/LDA were extracted. The definition of flare in studies should capture most real‐world flares [24], but not substantially differ from the validated standard for defining a flare [25], otherwise, equivalent outcomes were used rather than flare. Secondary outcomes included radiographic progression and safety outcomes. Radiographic progression was principally assessed using the Sharp–van der Heijde (SvdH) scoring system, and to minimize over‐interpretation of structural change due to measurement error, we extracted the number of patients exceeding the smallest detectable change (SDC) as defined in each study [26]. Safety outcomes were assessed as the number of patients experiencing any adverse event (AE) or serious adverse event (sAE), irrespective of the investigator's judgment of causality. When multiple analysis populations were reported, intention‐to‐treat (ITT) data were preferentially extracted, and per‐protocol (PP) data were used only when ITT data were unavailable.

To assess the durability of tapering or withdrawal, outcomes related to flare, relapse, or loss of remission/LDA were categorized by follow‐up duration. Preferred extraction windows were 6, 12, 24, and 36 months, or the closest available assessments. Outcomes were grouped as short‐term (< 9 months), intermediate‐term (9–18 months), or long‐term (> 18 months). When multiple outcomes were available within a time window, the most complete dataset was selected. Attempts were made to contact corresponding authors of publications to obtain missing data. All extracted data were cross‐checked independently by at least two reviewers, with disagreements resolved by consensus or adjudication.

### 2.5 Quality Assessment

The risk of bias of included randomized controlled trials was assessed using the Cochrane Risk of Bias 2.0 (RoB 2) tool [27]. Two reviewers independently evaluated the risk of bias for each study across the five RoB 2 domains: bias arising from the randomization process; bias due to deviations from intended interventions; bias due to missing outcome data; bias in measurement of the outcome; and bias in selection of the reported result. Assessments were guided by the RoB 2 signaling questions and the prespecified analysis approach for each study, including protocol or registry information when available. Each domain was judged as “low risk of bias,” “some concerns,” or “high risk of bias,” and an overall risk‐of‐bias judgment was derived according to RoB 2 guidance. Disagreements were resolved through discussion or consultation with a third reviewer.

### 2.6 Meta‐Analysis

Pairwise meta‐analyses were performed following standard Cochrane methods using RevMan (version 5.4.1). When multiple reports were available for a single trial, data from the most complete publication were used. However, for time‐specific analyses, the report corresponding to the relevant follow‐up window was selected to ensure that each participant contributed only once.

Risk ratios (RRs) with 95% confidence intervals (95% CI) were calculated for dichotomous outcomes. Random‐effects models were applied to outcomes where clinical heterogeneity was anticipated, such as flare in any DMARD tapering at any follow‐up duration, while fixed‐effect models were used for outcomes expected to exhibit minimal heterogeneity. Heterogeneity was quantified using the *I* ^2^ and *τ* ^2^ statistics. For sparse outcomes, such as radiographic progression and sAEs, fixed‐effect Mantel–Haenszel pooling served as the primary approach, supplemented by random‐effects models and rare‐event methods in sensitivity analyses.

Subgroup analyses were conducted primarily based on DMARD class (csDMARD vs. bDMARD vs. tsDMARD) and follow‐up duration categories (short‐term vs. intermediate‐term vs. long‐term). Sensitivity analyses included restricting to validated definition of flare, examining alternative effect measures and statistical models, conducting leave‐one‐out (LOO) influence analyses, excluding studies with high RoB, etc.

## 3 Results

### 3.1 Study Selection and Characteristics

The literature search identified 522 records in PubMed and 737 records in Embase. The Cochrane Library search identified 234 records from ICTRP, ClinicalTrials.gov, and CINAHL, without duplicating records retrieved from PubMed or Embase. The selection process is summarized in Figure 1. Particularly, three studies [28, 29, 30] were additionally identified through reference screening of relevant publications. One interim report [31] was excluded because the complete results were subsequently published [10]. One long‐term follow‐up report [32] of an included trial [11] was excluded because the treatment regimen changed during the extended observational phase.

In total, 27 publications (representing 23 trials) met the eligibility criteria and were included in the analyses. Notably, we included two publications from the DRESS trial [12, 13], and four publications from the ARCTIC REWIND trial [7, 8, 33, 34]. The 27 included publications comprised 5262 patients with RA and sustained disease control, including 11 reports enrolling patients with sustained LDA and 16 reports enrolling patients with sustained remission. Across studies, 7 reports [11, 29, 34, 35, 36, 37, 38] compared a DMARD tapering strategy with treatment continuation, 12 reports [6, 7, 9, 12, 13, 28, 33, 39, 40, 41, 42, 43] compared DMARD withdrawal with continuation, and 8 reports [8, 10, 30, 44, 45, 46, 47, 48] evaluated both tapering and withdrawal strategies against continuation.

Participant characteristics were broadly consistent with typical RA cohorts in sustained remission or LDA. Disease duration varied, with many pragmatic tapering/withdrawal trials enrolling patients with long‐standing RA [6, 40, 41], whereas several studies enrolled earlier disease cohorts following initial intensive therapy [30, 45]. Baseline disease activity was uniformly low by design, most commonly defined using DAS28‐ or simplified disease activity index (SDAI)‐based remission criteria, resulting in generally comparable “remission‐selected” populations across trials despite differences in intervention approaches.

Background therapy varied across studies, but participants were generally receiving stable DMARD regimens before de‐escalation, including monotherapy and combination therapy (often a bDMARD with a csDMARD). Several trials were conducted in multicenter settings across Europe, Asia, and multinational networks. Although DMARD classes and de‐escalation protocols differed (dose reduction, interval prolongation, or discontinuation), all included trials required remission or LDA for a predefined period at randomization, resulting in broadly similar entry disease‐control status across studies.

Detailed study characteristics are presented in Table 1.

**TABLE 1:** Characteristics of studies included in the meta‐analysis.

Report, year (trial)	Duration of disease control	Disease activity^a^ at randomization	Grouping	Tapering/withdrawal strategy	Flare/relapse definition	Primary endpoints	Follow‐up (month)	Design
Ward 2025 (STARA) [6]	Sustained remission ≥ 6 months on csDMARDs plus bDMARDs	1.35 ± 0.35 1.55 ± 0.37 (DAS28‐CRP)	Standard bDMARDs (TNFi); withdraw bDMARDs	Complete withdrawal	Flare: △DAS28‐CRP ≥ 1.2 Relapse: DAS28‐CRP ≥ 2.6^b^	% of relapses over 11.1 months	11.1	NI
Asai 2025 (PRIMERA) [39]	Sustained LDA ≥ 12 weeks on csDMARDs plus bDMARDs	1.6 (1.5–1.8) 1.5 (1.4–1.7)^c^ (DAS28‐CRP)	Standard csDMARDs (MTX); Taper csDMARDs to withdrawal	Tapering for 12 weeks followed by withdrawal	Flare: CDAI > 10 or intervention with rescue	% of LDA without a flare at 8.3 months	8.3	NI
Kjørholt 2025 (ARCTIC REWIND‐TNFi) [7]	Sustained remission ≥ 12 months on TNFi plus 90% of csDMARDs	0.9 ± 0.4 0.8 ± 0.3 (DAS44)	Standard bDMARDs (TNFi); Taper bDMARDs to withdrawal	Tapering for 4 months followed by withdrawal	Flare: DAS44 > 1.6, △DAS44 ≥ 0.6, SJC ≥ 2/44^b^	Flare‐free survival and radiographic progression over 36 months	36	NI
Kjørholt 2024 (ARCTIC REWIND‐csDMARDs) [8]	Sustained remission ≥ 12 months on csDMARDs	0.8 ± 0.4 0.8 ± 0.3 0.8 ± 0.3 (DAS44)	Standard csDMARDs; Taper csDMARDs; Taper csDMARDs to withdrawal	Tapering: half dose; tapering to withdrawal: half dose for the 1st year followed by withdrawal	Flare: DAS44 > 1.6, △DAS44 ≥ 0.6, SJC ≥ 2/44^b^	% of flares over 36 months	36	NI
Kedra 2024 (ToLEDo) [9]	Sustained remission ≥ 6 months on bDMARDs or plus csDMARDs	0.8 ± 0.5 1.0 ± 0.5 (DAS44)	Standard bDMARDs (IL‐6 or CTLA‐4 inhibitors); Taper bDMARDs to withdrawal	Four gradual reductions at a 3‐month interval to withdrawal	Flare: DAS28‐ESR > 3.2	ΔDAS44 over 24 months	24	NI
Lillegraven 2023 (ARCTIC REWIND‐TNFi) [33]	Sustained remission ≥ 12 months on TNFi plus 90% of csDMARDs	0.9 ± 0.4 0.8 ± 0.3 (DAS44)	Standard bDMARDs (TNFi); Taper bDMARDs to withdrawal	Tapering for 4 months followed by withdrawal	Flare: DAS44 > 1.6, △DAS44 ≥ 0.6, SJC ≥ 2/44^b^	% of flares over 12 months	12	NI
Uhrenholt 2023 (BIODOPT) [35]	Sustained LDA ≥ 12 months on TNFi	1.8 ± 0.5 1.7 ± 0.4 (DAS28‐CRP)	Standard bDMARDs (TNFi); Taper bDMARDs	Disease activity‐guided tapering strategy	Flare: ΔDAS28‐CRP > 1.2, or ΔDAS28‐CRP > 0.6 when DAS28‐CRP ≥ 3.2	% of achieving biological reduction by half while maintaining equivalent disease activity	18	Equivalence
Wang 2023 (N/A) [44]	Sustained remission or LDA ≥ 3 months on tsDMARDs	2.3 ± 0.6 2.1 ± 0.6 2.2 ± 0.7 (DAS28‐ESR)	Standard tsDMARDs (JAKi); Taper tsDMARDs; withdraw tsDMARDs	Tapering: half dose; withdrawal: complete withdrawal	Flare: repeat DAS28‐ESR > 3.2	% of LDA at 6 months	6	Comparative
Emery 2023 (AVERT‐2) [30]	Sustained remission ≥ 12 weeks on bDMARDs plus csDMARDs	1.63–1.79^d^ (DAS28‐CRP)	Standard DMARDs (TNFi + MTX); Taper bDMARDs to withdrawal; withdraw csDMARDs	Tapering to withdrawal: spaced dosing interval for 6 months followed by withdrawal; withdrawal: complete withdrawal	N/A	% of SDAI remission 7.4 months prior to randomization	11.1	Comparative
Bertrand 2022 (TapERA) [36]	Sustained remission ≥ 6 months on bDMARDs	1.9 (1.4–2.4) 1.7 (1.3–2.1) (DAS28‐CRP)^e^	Standard bDMARDs (TNFi); Taper bDMARDs	Spaced dosing interval	N/A	% of DAS28‐CRP remission at 6 months	12	Comparative
Tascilar 2021 (RETRO) [10]	Sustained remission ≥ 6 months on csDMARDs or bDMARDs or both	1.7 ± 0.7 1.7 ± 0.6 1.7 ± 0.6 (DAS28‐ESR)	Standard DMARDs; Taper DMARDs; Taper to withdrawal	Tapering: half dose; tapering to withdrawal: half dose for 6 months followed by withdrawal	Relapse: DAS28‐ESR ≥ 2.6	% of DAS28‐CRP remission at 6 months	12	Comparative
Lillegraven 2021 (ARCTIC REWIND‐csDMARDs) [34]	Sustained remission > 12 months on csDMARDs	0.8 ± 0.4 0.8 ± 0.3 (DAS44)	Standard csDMARDs; Taper csDMARDs	Half dose	Flare: DAS44 > 1.6, △DAS44 ≥ 0.6, SJC ≥ 2/44^b^	% of flare over 12 months	12	NI
Curtis 2021 (SEAM‐RA) [28]	Sustained remission ≥ 24 weeks on bDMARDs plus csDMARDs	1.2 ± 1.2 1.3 ± 1.0 1.3 ± 1.4 (SDAI)	Standard DMARDs (MTX + TNFi); withdraw bDMARDs; withdraw csDMARDs	Complete withdrawal	Disease‐worsening: 3.3 < SDAI ≤ 11 for 3 visits or 2 visits for 2 weeks, or SDAI > 11	% of SDAI remission without disease worsening at 11.1 months	11.1	Comparative
Sanmarti 2019 (TOZURA) [29]	Sustained remission ≥ 4 weeks on bDMARDs or plus csDMARDs	N/A	Standard bDMARDs (anti‐IL‐6); Taper bDMARDs	Spaced dosing interval	Relapse: DAS28‐ESR ≥ 2.6	Not clearly defined	5.5	Comparative
Verhoef 2019 (REDO) [11]	Sustained LDA ≥ 6 months on bDMARDs or plus csDMARDs	2.45 ± 0.92 2.30 ± 0.96 2.56 ± 1.09 (DAS28‐CRP)	Standard bDMARDs (anti‐CD20); Taper bDMARDs to different doses	Half dose or 1/5 dose	Flare: △DAS28‐CRP > 1.2, or △DAS28‐CRP > 0.6 plus DAS28‐CRP ≥ 2.9 at 6 months	△DAS28‐CRP at 3 months and 6 months	6	NI
Takeuchi 2019 (RA‐BEYOND, baricitinib reduction) [37]	Sustained LDA or remission ≥ 3 months on tsDMARDs	2.73 ± 0.82 2.66 ± 0.93 (DAS28‐ESR)	Standard tsDMARDs (JAKi); Taper tsDMARDs	Half dose	Relapse: CDAI > 10	% of CDAI LDA at 3 months, time to relapse after randomization	11.1	Comparative
Bouman 2017 (DRESS) [12]	Sustained LDA ≥ 3 months on bDMARDs	2.5 ± 0.8 2.5 ± 0.7 (DAS28‐ESR)	Standard bDMARDs (TNFi); Taper bDMARDs to withdrawal	Stepwise spaced dosing interval followed by withdrawal	Flare: △DAS28‐CRP > 1.2, or △DAS28‐CRP > 0.6 when DAS28‐CRP ≥ 3.2	% of major flares^f^ over 36 months	36	NI
Weinblatt 2017 (C‐EARLY) [45]	Sustained LDA ≥ 3 months on bDMARDs plus csDMARDs	2.0 ± 0.7 2.0 ± 0.6 1.9 ± 0.7 (DAS28‐ESR)	Standard bDMARDs (TNFi); Taper bDMARDs; withdraw bDMARDs	Tapering: spaced dosing interval; Withdrawal: complete withdrawal	Flare: DAS28‐ESR > 3.2, △DAS28 ≥ 0.6, duration ≥ 2 weeks, plus confirmed by the investigator's judgment	% of DAS28‐ESR LDA without flares at 12 months	12	Comparative
Yamanaka 2016 (ENCOURAGE) [40]	Sustained remission > 6 months on bDMARDs plus csDMARDs	1.7 ± 0.5 1.8 ± 0.5 (DAS28‐ESR)	Standard bDMARDs (TNFi); withdraw bDMARDs	Complete withdrawal	Flare: DAS28‐ESR ≥ 3.2	% of all remissions at 12 months	12	Comparative
van Vollenhoven 2016 (DOSERA) [46]	Sustained LDA ≥ 8 weeks on csDMARDs plus bDMARDs	1.9 ± 0.6 1.9 ± 0.9 1.9 ± 0.7 (DAS28‐ESR)	Standard bDMARDs (TNFi); Taper bDMARDs; withdraw bDMARDs	Tapering: half dose; withdrawal: complete withdrawal	Flare: DAS28‐ESR > 5.1, or DAS28‐ESR > 3.2 and ΔDAS28 ≥ 1.2, or DAS28 > 3.2 and ΔDAS28 ≥ 0.6 on 2 consecutive visits at least 1–3 weeks apart^b^	% of non‐failures at 11.1 months (standard vs. withdrawal)	11.1	Superiority
Moghadam 2016 (POET) [41]	Sustained LDA ≥ 6 months on csDMARDs plus bDMARDs	2.05 ± 0.73 1.98 ± 0.76 (DAS28‐ESR)	Standard bDMARDs (TNFi); withdraw bDMARDs	Complete withdrawal	Flare: DAS28‐ESR ≥ 3.2, ΔDAS28 ≥ 0.6	% of flares over 12 months	12	Superiority
Fautrel 2016 (STRASS) [42]	Sustained remission ≥ 6 months on bDMARDs or plus csDMARDs	1.7 ± 0.5 1.9 ± 0.6 (DAS28‐ESR)	Standard bDMARDs (TNFi); Taper bDMARDs to withdrawal	Disease activity‐guided tapering strategy followed by withdrawal	Relapse: DAS28‐ESR > 2.6 and △DAS28 > 0.6	Standardized difference of DAS28 slopes over 18 months	18	Equivalence
El Miedany 2016 (N/A) [47]	Sustained remission ≥ 6 months on csDMARDs plus bDMARDs	2.2 ± 0.5 1.97 ± 0.4 2.1 ± 0.3 2.1 ± 0.2 2.04 ± 0.4 (DAS28‐ESR)	Standard csDMARDs plus bDMARDs; Taper bDMARDs; Taper both; Taper csDMARDs plus withdraw bDMARDs; withdraw both	Tapering: half dose; withdrawal: complete withdrawal	Relapse: DAS28‐ESR > 3.2	% of sustained remission over 12 months	12	Comparative
Chatzidionysiou 2016 (ADMIRE) [43]	Sustained remission ≥ 3 months on csDMARDs plus bDMARDs	2.13 (1.6–2.4) 1.69 (1.5–2.37)^e^ (DAS28‐ESR)	Standard bDMARDs; withdraw bDMARDs	Complete withdrawal	Flare: DAS28‐ESR ≥ 2.6 or △DAS28 > 1.2	% of remission at 6.4 months (interim)	6.4	Superiority
van Herwaarden 2015 (DRESS) [13]	Sustained LDA ≥ 3 months on bDMARDs	2.5 ± 0.8 2.5 ± 0.7 (DAS28‐ESR)	Standard bDMARDs (TNFi); Taper bDMARDs to withdrawal	Stepwise spaced dosing interval followed by withdrawal	Flare: △DAS28‐CRP > 1.2, or △DAS28‐CRP > 0.6 when DAS28‐CRP ≥ 3.2	% of major flares over 18 months (a switch from all flares to major flares^f^)	18	NI
Raffeiner 2015 (N/A) [38]	Sustained remission ≥ 12 months on bDMARDs or plus csDMARDs	N/A	Standard bDMARDs (TNFi); Taper bDMARDs	Spaced dosing interval	Flare: DAS28‐ESR > 2.6	Not clearly defined	43.2 ± 18	Superiority
Smolen 2013 (PRESERVE) [48]	Sustained LDA ≥ 24 weeks on csDMARDs plus bDMARDs	2.0 ± 0.6 2.1 ± 0.6 2.1 ± 0.6 (DAS28‐ESR)	Standard bDMARDs (TNFi); Taper bDMARDs; withdraw bDMARDs	Tapering: half dose; withdrawal: complete withdrawal	Loss of efficacy: DAS28‐ESR > 3.2 plus △DAS28 ≥ 0.6	% of LDA at 12 months (standard vs. withdrawal)	12	Superiority

Abbreviations: CRP, C‐reactive protein; DAS, disease activity score; ESR, erythrocyte sedimentation rate; JAKi, Janus kinas inhibitor; LDA, low disease activity; MTX, methotrexate; NI, non‐inferiority; SJC, swollen joint count; TNFi, tumor necrosis factor inhibitor.

^a^ Mean ± SD unless otherwise noted.

^b^ Flare can also be defined by the communications between doctors and patients.

^c^ Mean (95% CI).

^d^ Range of means across groups.

^e^ Median (IQR).

^f^ Major flares defined as lasting for longer than 3 months, otherwise classified as short‐lived flares.

Following the RoB 2 tool [27], most of the included studies were judged as overall “low risk” or “some concerns.” A few trials [36, 38, 39, 40, 41, 43, 44, 47] were judged as “high risk,” mostly due to design drawbacks. Detailed evaluation of RoB of each study is displayed in Figure 2.

**FIGURE 2:**
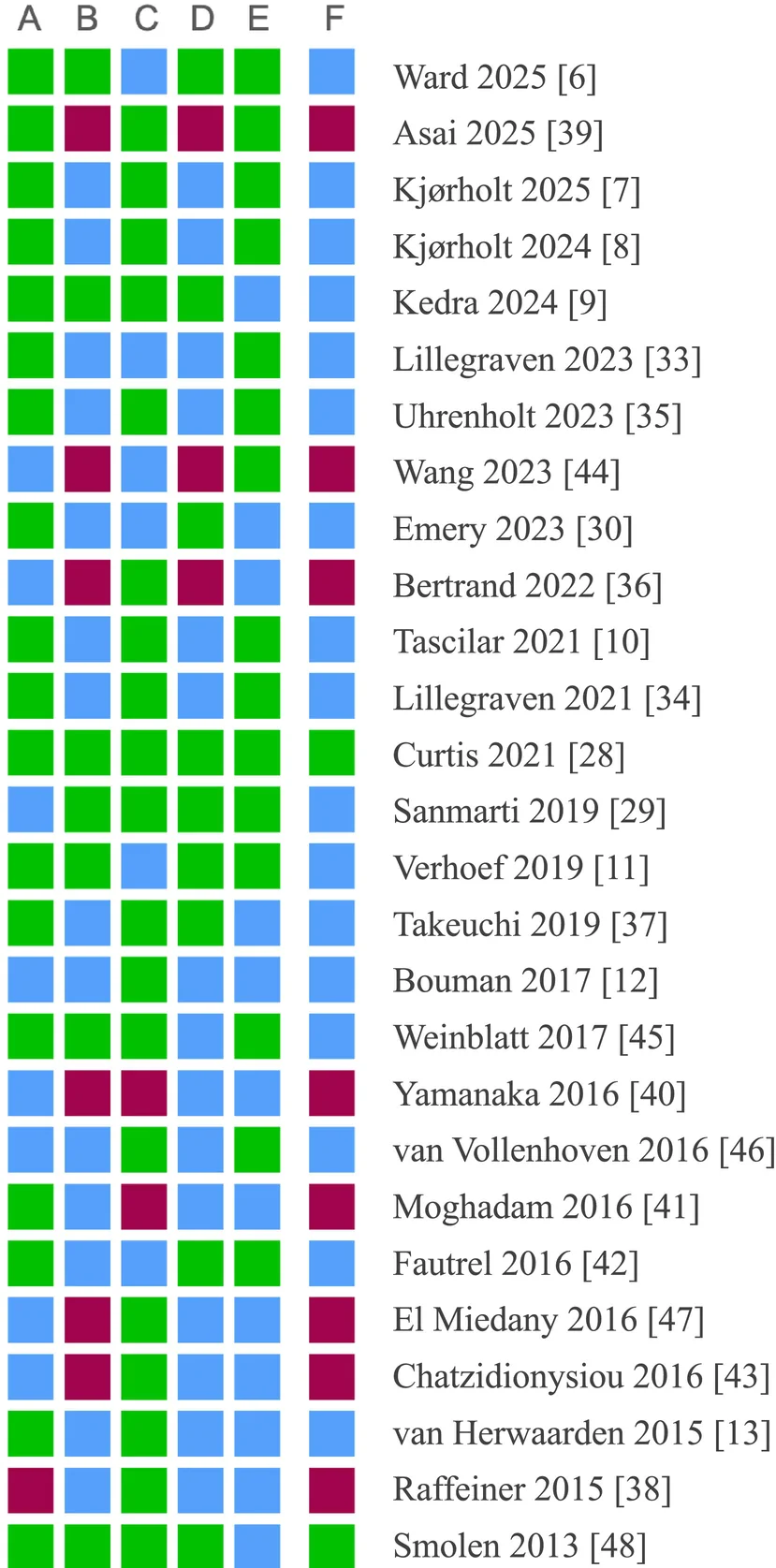
Quality assessment of studies included in the meta‐analysis based on the revised risk of bias tool. Risk of bias derived from randomization process (A), deviations from intended interventions (B), missing outcome data (C), measurement of the outcome (D), selection of the reported result (E), and overall risk of bias (F). Bias was judged as low risk (green), some concerns (blue), and high risk (plum) within each domain and overall for each study.

### 3.2 Disease Activity

#### 3.2.1 Tapering

We first meta‐analyzed 15 publications [8, 10, 11, 29, 30, 34, 35, 36, 37, 38, 44, 45, 46, 47, 48] comparing DMARD tapering strategies with continued standard treatments. Overall, any DMARD tapering was associated with a higher risk of disease flare (pooled RR 1.56, 95% CI 1.23–1.98), with moderate‐to‐high heterogeneity (*τ* ^2^ = 0.11, *I* ^2^ = 64%) (Figure S1a). Visual inspection of the funnel plot suggested possible publication bias, with smaller studies showing more variable and, in some cases, larger effect estimates (Figure S1b).

Because tapering effects were anticipated to differ by DMARD class, we conducted prespecified subgroup analyses. The pooled RRs for disease flare were 1.21 (95% CI 0.99–1.50) for bDMARD tapering, 2.14 (95% CI 1.23–3.74) for csDMARD tapering, 2.02 (95% CI 0.86–4.79) for tsDMARD tapering, and 4.28 (95% CI 1.11–16.57) for tapering all DMARDs (Figure S2). Heterogeneity was attenuated in the bDMARD tapering subgroup (*τ* ^2^ = 0.03, *I* ^2^ = 29%). In contrast, heterogeneity remained high in the tsDMARD and all‐DMARDs subgroups, and could not be meaningfully assessed for csDMARD tapering because only one study contributed to that estimate (Figure S2).

Next, we stratified studies by DMARD type and follow‐up duration.

Within the bDMARD tapering subgroup, we further examined the influence of follow‐up duration by grouping outcomes as short‐term (< 9 months), intermediate‐term (9–18 months), and long‐term (> 18 months). The risk of flare was increased in the short term (RR 1.61, 95% CI 1.13–2.31; *p* = 0.009), but not in the intermediate term (RR 1.10, 95% CI 0.91–1.33; *p* = 0.33) or long term (RR 0.96, 95% CI 0.61–1.52; *p* = 0.88) (Figure S3). Heterogeneity was further reduced within the short‐term (*τ* ^2^ = 0.01, *I* ^2^ = 7%) and intermediate‐term groups (*τ* ^2^ = 0, *I* ^2^ = 0%) (Figure S3). Only one study contributed data to the long‐term bDMARD tapering category, precluding estimation of heterogeneity. Funnel plot inspection did not indicate clear asymmetry (Figure S4). To assess robustness, we repeated analyses using odds ratios (ORs). The corresponding ORs were 1.99 (95% CI 1.26–3.13; *p* = 0.003) in the short term, 1.20 (95% CI 0.87–1.65; *p* = 0.26) in the intermediate term, and 0.96 (95% CI 0.55–1.68; *p* = 0.88) in the long term (Figure S5), consistent with the RR findings. Overall, these results indicate a significantly increased risk of flare following bDMARD tapering in the short term (< 9 months), whereas this risk was not sustained at longer follow‐up.

Studies for other tapering subgroups were few. Therefore, we summarized results by follow‐up duration without further interpreting these findings. For csDMARD tapering, only ARCTIC REWIND reports [8, 34] evaluated flare risk among patients with sustained remission, and the estimates are shown in Figure S6. For tsDMARD tapering, one study [44] reported short‐term outcomes, and the other [37] reported outcomes through 12 months. Estimated risks are shown in Figure S7. For tapering of all DMARD types, two studies reported outcomes through 12 months, with pooled estimates presented in Figure S8.

Pooled estimates of flare risk following tapering are summarized in Table 2.

**TABLE 2:** Primary outcome (flare risk) upon DMARD tapering or withdrawal.

Risk ratio	Tapering				Withdrawal			
	Any DMARD	csDMARDs	bDMARDs	tsDMARDs	Any DMARD	csDMARDs	bDMARDs	tsDMARDs
Any time	**1.56 (1.23–1.98)**	2.14 (1.23–3.74)^a^	1.21 (0.99–1.50)	2.02 (0.86–4.79)	**2.32 (1.83–2.94)**	1.62 (0.92–2.84)	**2.12 (1.70–2.64)**	8.15 (3.17–20.95)^a^
< 9 m	—	5.57 (1.28–24.31)^a^	**1.61 (1.13–2.31)**	2.12 (1.04–4.30)^a^	—	1.44 (0.95–2.18)	**2.47 (1.84–3.32)**	8.15 (3.17–20.95)^a^
9–18 m	—	4.00 (1.58–10.12)^a^	1.10 (0.91–1.33)	1.47 (1.15–1.88)^a^	—	1.24 (0.84–1.85)	**2.30 (1.77–2.99)**	N/A
> 18 m	—	2.14 (1.23–3.74)^a^	0.96 (0.61–1.52)	N/A	—	3.16 (1.92–5.20)^a^	**2.48 (1.67–3.69)**	N/A

*Note:* Significantly increased risk ratios (RRs) of flare from at least two studies are labeled bold.

Abbreviation: N/A, no available results reported.

^a^ Effect size from one study only.

#### 3.2.2 Withdrawal

We next meta‐analyzed 20 publications [6, 7, 8, 9, 10, 12, 13, 28, 30, 33, 39, 40, 41, 42, 43, 44, 45, 46, 47, 48] comparing DMARD withdrawal strategies with continued standard treatment. Overall, any DMARD withdrawal was associated with a substantially higher risk of disease flare (pooled RR 2.32, 95% CI 1.83–2.94; *p* < 0.00001), with considerable heterogeneity (*τ* ^2^ = 0.18, *I* ^2^ = 80%) (Figure S9a). Funnel plot inspection suggested possible publication bias, with smaller studies tending to report larger risk ratios (Figure S9b).

Also, we conducted prespecified subgroup analyses by DMARD type and follow‐up duration. The pooled RRs for flare were 2.12 (95% CI 1.70–2.64; *p* < 0.00001) for bDMARD withdrawal, 1.62 (95% CI 0.92–2.84; *p* = 0.10) for csDMARD withdrawal, 8.15 (95% CI 3.17–20.95) for tsDMARD withdrawal, and 5.35 (95% CI 1.45–19.79) for all DMARD withdrawal (Figure S10). Funnel plot inspection at the subgroup level continued to suggest possible small‐study effects (Figure S11).

##### 3.2.2.1 bDMARDs

Within the bDMARD withdrawal subgroup, heterogeneity remained high (*τ* ^2^ = 0.11, *I* ^2^ = 75%) (Figure S10a). Likewise, we examined follow‐up duration by grouping outcomes as short‐term (< 9 months), intermediate‐term (9–18 months), and long‐term (> 18 months).

In the short term, bDMARD withdrawal significantly increased flare risk (RR 2.47, 95% CI 1.84–3.32; *p* < 0.00001), with reduced but still moderate heterogeneity (*τ* ^2^ = 0.08, *I* ^2^ = 46%) (Figure S12a). Funnel plot inspection showed apparent publication bias, largely driven by the ARCTIC REWIND study [33] (Figure S12b). In LOO analyses, exclusion of individual studies did not change the direction or statistical significance of the pooled estimate. Particularly, excluding the ARCTIC REWIND study [33] materially reduced heterogeneity (*τ* ^2^ = 0.03, *I* ^2^ = 28%), suggesting a potential small‐study effect. Still, the effect size remained similar (RR 2.41, 95% CI 1.88–3.09; *p* < 0.00001) (Figure S13a). A further sensitivity analysis using a fixed‐effect model yielded consistent findings (RR 2.88, 95% CI 2.35–3.53; *p* < 0.00001) (Figure S13b). Collectively, these analyses support a robust short‐term increase in flare risk after bDMARD withdrawal.

In the intermediate term, bDMARD withdrawal also increased flare risk (RR 2.30, 95% CI 1.77–2.99; *p* < 0.00001), although heterogeneity remained high (*τ* ^2^ = 0.15, *I* ^2^ = 79%) (Figure S14a). Funnel plot inspection did not show pronounced asymmetry, but several small studies reported comparatively large risk ratios (Figure S14b). Sensitivity analyses did not materially change the findings: (1) LOO analyses did not meaningfully reduce heterogeneity; (2) sensitivity analysis using ORs yielded a pooled OR of 4.46 (95% CI 3.28–6.07; *p* < 0.00001) with reduced but persistent heterogeneity (*τ* ^2^ = 0.14, *I* ^2^ = 54%) (Figure S15a); (3) restricting analyses by excluding high RoB studies produced similar results (RR 2.25, 95% CI 1.72–2.94) with ongoing heterogeneity (*τ* ^2^ = 0.15, *I* ^2^ = 80%) (Figure S15b). Additional subgroup analyses stratified by disease state at randomization (remission vs. LDA; Figure S16), strategy type (withdrawal vs. taper‐to‐withdrawal; Figure S17), concomitant csDMARD use (Figure S18), and whether flare was explicitly defined (Figure S19a) did not substantially resolve heterogeneity. However, within the “flare defined” and “flare not defined” strata, LOO analyses solved the heterogeneity by identifying one influential study in each stratum [45, 48] (Figure S19b). Importantly, across all analyses the direction and statistical significance of the effect were unchanged. Overall, these findings indicate a robust intermediate‐term increase in flare risk following bDMARD withdrawal, albeit with considerable residual heterogeneity.

In the long term, bDMARD withdrawal remained associated with a higher risk of flare (RR 2.48, 95% CI 1.67–3.69; *p* < 0.00001), again with substantial heterogeneity (*τ* ^2^ = 0.08; *I* ^2^ = 66%) (Figure S20a). LOO analysis identified the ARCTIC REWIND study [7] as the major contributor to heterogeneity, but excluding it did not alter the direction or significance of the pooled effect (Figure S20b).

Taken together, bDMARD withdrawal was consistently associated with higher flare risk across short‐, intermediate‐, and long‐term follow‐up.

##### 3.2.2.2 Other DMARDs

For csDMARD withdrawal, heterogeneity was high in the overall subgroup analysis (*τ* ^2^ = 0.24, *I* ^2^ = 76%) (Figure S10b). When stratified by follow‐up duration, csDMARD withdrawal was not associated with a statistically significant increase in flare risk in the short term (RR 1.44, 95% CI 0.95–2.18; *τ* ^2^ = 0, *I* ^2^ = 0%; *p* = 0.08) (Figure S21a) or the intermediate term (RR 1.24, 95% CI 0.84–1.85; *τ* ^2^ = 0.03, *I* ^2^ = 35%; *p* = 0.28) (Figure S21b). For long‐term follow‐up, only the ARCTIC REWIND study [8] contributed data (RR 3.16, 95% CI 1.92–5.20) (Figure S21c). Overall, these analyses did not demonstrate a significantly increased flare risk following csDMARD withdrawal through 12 months, while evidence for longer follow‐up remained insufficient.

For tsDMARD withdrawal, only one trial [44] evaluated withdrawal in patients with sustained remission/LDA. In this study, JAK inhibitor withdrawal substantially increased flare risk at 6 months (RR 8.15, 95% CI 3.17–20.95) (Figure S22), and no studies reported longer follow‐up.

Two studies [10, 47] assessed withdrawal of all DMARD classes. The short‐term estimate (from one study) suggested a markedly increased flare risk (RR 4.63, 95% CI 2.39–8.95) (Figure S23a), and the intermediate‐term pooled estimate (from two studies) was similarly elevated (RR 5.35, 95% CI 1.45–19.79) (Figure S23b). No data were available beyond 12 months for this comparison.

In summary, withdrawal of bDMARDs was consistently associated with increased flare risk across follow‐up durations (Table 2). For csDMARD withdrawal, the pooled evidence did not show a statistically significant increase in flare risk through 12 months, although longer‐term evidence was limited. Evidence on tsDMARD withdrawal and withdrawal of multiple DMARD classes was sparse but suggested substantially increased flare risk, highlighting the need for avoidance.

### 3.3 Structural Progression

To evaluate the impact of DMARD tapering or withdrawal on structural outcomes, we pooled studies reporting radiographic progression using a clearly defined SDC, which reduces the likelihood of overinterpreting minor score fluctuations that may reflect measurement error rather than true progression, thereby focusing the analysis on reliably detected structural change.

#### 3.3.1 Tapering

Only three studies [8, 34, 48] reported radiographic progression using an SDC threshold. Two studies reported outcomes through 12 months, and one reported outcomes through 36 months. In the intermediate‐term follow‐up, DMARD tapering was associated with a higher risk of radiographic progression (pooled RR 2.17, 95% CI 1.05–4.45; *I* ^2^ = 0%; *p* = 0.04), with the significance driven by the ARCTIC REWIND study [34] (Figure S24a). For long‐term follow‐up, evidence was limited to another ARCTIC REWIND report [8], which yielded a markedly increased risk of progression (RR 13.22, 95% CI 1.69–103.32) (Figure S24b).

#### 3.3.2 Withdrawal

Eight studies reported radiographic progression using an SDC threshold. Four studies [6, 13, 33, 48] contributed intermediate‐term outcomes, four studies [7, 8, 9, 12] contributed long‐term outcomes, and no study reported short‐term radiographic progression following DMARD withdrawal.

In the intermediate term, DMARD withdrawal was associated with increased progression risk (pooled RR 2.64, 95% CI 1.27–5.49; *I* ^2^ = 38%; *p* = 0.009) (Figure S25a). LOO sensitivity analysis identified the STARA study [6] as the main contributor to heterogeneity, and excluding it eliminated heterogeneity and maintained the direction and statistical significance of the effect (RR 3.29, 95% CI 1.47–7.36; *I* ^2^ = 0%; *p* = 0.004) (Figure S26a).

In the long term, DMARD withdrawal remained associated with a higher risk of radiographic progression (pooled RR 2.82, 95% CI 1.39–5.75; *I* ^2^ = 0%; *p* = 0.004) (Figure S25b). Sensitivity analysis using a random‐effects model yielded consistent results (RR 2.57, 95% CI 1.26–5.27; *I* ^2^ = 0%; *p* = 0.01) (Figure S26b). Excluding the highest‐weight study also did not materially alter the findings (RR 3.74, 95% CI 1.36–10.32; *I* ^2^ = 0%; *p* = 0.01) (Figure S26c).

Taken together, both tapering (weak evidence) and withdrawal (strong evidence) were associated with an increased risk of radiographic progression (Table 3).

**TABLE 3:** Secondary outcomes (radiographic progression and adverse events) upon DMARD tapering or withdrawal.

Risk ratio	Tapering	Withdrawal
Radiographic progression		
< 9 m	N/A	N/A
9–18 m	**2.17 (1.05–4.45)**	**2.64 (1.27–5.49)**
> 18 m	13.22 (1.69–103.32)^a^	**2.82 (1.39–5.75)**
Adverse events		
Any AEs	1.04 (0.97–1.12)	1.01 (0.92–1.10)
Serious AEs	0.89 (0.64–1.24)	**1.31 (1.04–1.65)**

*Note:* Significantly increased risk ratios (RRs) of secondary outcomes from at least two studies are labeled bold.

Abbreviation: N/A, no available results reported.

^a^ Effect size from one study only.

### 3.4 Safety

Last, we evaluated the safety of DMARD tapering or withdrawal by analyzing the proportion of patients experiencing at least one adverse event (AE) and at least one serious adverse event (sAE) under each strategy.

#### 3.4.1 Tapering

Ten studies [8, 29, 34, 35, 36, 37, 44, 45, 46, 48] reported the occurrence of any AE following DMARD tapering. Tapering was not associated with an increased risk of any AE (pooled RR 1.04, 95% CI 0.97–1.12; *p* = 0.30), and no heterogeneity was detected (*τ* ^2^ = 0, *I* ^2^ = 0%) (Figure S27a). We further conducted a meta‐analysis evaluating the risk of infection, one of the most clinically relevant adverse events associated with DMARD treatment. Consistent results were observed when infections were analyzed separately (pooled RR 0.95, 95% CI 0.80–1.13; *p* = 0.56), with low heterogeneity (*τ* ^2^ = 0.01, *I* ^2^ = 20%) (Figure S28a). These findings were further supported by sensitivity analysis using OR as the effect measure (Figure S28b). Malignancies were not analyzed because only a few studies [8, 34, 45, 48] reported this relatively rare outcome, and the short follow‐up durations of the included trials were unlikely to capture meaningful differences in cancer incidence, which generally requires substantially longer observation periods. Subgroup analyses yielded similarly nonsignificant results across DMARD classes, with no heterogeneity detected within subgroups either (Figure S29). Overall, these findings suggest that DMARD tapering does not materially change the risk of any AE.

Twelve studies [8, 10, 11, 15, 29, 34, 35, 37, 44, 45, 46, 48] reported serious AEs under tapering regimens. The pooled estimate did not indicate an increased risk of sAEs (RR 0.89, 95% CI 0.64–1.24; *p* = 0.48), with no heterogeneity detected (*I* ^2^ = 0%) (Figure S30a). Subgroup analyses were likewise nonsignificant across all subgroups, with no detectable heterogeneity (Figure S31). Collectively, these results suggest that DMARD tapering does not change the risk of serious AEs.

#### 3.4.2 Withdrawal

Eighteen studies [6, 7, 8, 9, 12, 13, 28, 30, 33, 39, 40, 41, 42, 43, 44, 45, 46, 48] reported any AEs following DMARD withdrawal. Overall, DMARD withdrawal was not associated with the risk of any AE (RR 1.01, 95% CI 0.92–1.10; *p* = 0.91), although moderate heterogeneity was observed (*τ* ^2^ = 0.02, *I* ^2^ = 58%) (Figure S27b). Subgroup analyses remained nonsignificant for both bDMARD withdrawal (RR 1.00, 95% CI 0.92–1.08; *I* ^2^ = 40%; *p* = 0.91) (Figure S32a) and csDMARD withdrawal (RR 1.05, 95% CI 0.82–1.33; *I* ^2^ = 57%; *p* = 0.72) (Figure S32b). Evidence for tsDMARD withdrawal was limited to a single study [44], which suggested a lower risk of any AE but insufficient to support firm conclusions (Figure S32c). Overall, the available evidence indicates that withdrawal of bDMARDs or csDMARDs does not materially alter the risk of any AE.

Eighteen studies [6, 7, 8, 9, 10, 12, 13, 28, 30, 33, 39, 41, 42, 43, 44, 45, 46, 48] reported serious AEs following DMARD withdrawal. In the overall analysis, withdrawal was associated with a modestly increased risk of sAEs (RR 1.31, 95% CI 1.04–1.65; *p* = 0.02), with low heterogeneity (*I* ^2^ = 2%) (Figure S30b). In subgroup analyses, the increased risk was observed in the bDMARD withdrawal subgroup (RR 1.31, 95% CI 1.02–1.70; *p* = 0.04; *I* ^2^ = 10%) (Figure S33a), whereas csDMARD withdrawal (RR 0.94, 95% CI 0.34–2.57; *p* = 0.91) (Figure S33b) and tsDMARD withdrawal (RR 0.35, 95% CI 0.01–8.34; *p* = 0.52) (Figure S33c) were not associated with increased sAE risk. The estimate for withdrawal of multiple DMARD classes was also nonsignificant (RR 1.26, 95% CI 0.58–2.73; *p* = 0.56) (Figure S33d). We further stratified bDMARD withdrawal studies by follow‐up duration. The pooled RR for sAEs was 3.20 (95% CI 0.37–27.49; *p* = 0.29) in the short term (Figure S34a), 1.43 (95% CI 1.03–1.98; *p* = 0.03; *I* ^2^ = 0%) in the intermediate term (Figure S34b), and 1.28 (95% CI 0.89–1.83; *p* = 0.18; *I* ^2^ = 60%) in the long term (Figure S34c). However, the intermediate‐term significance was not robust in sensitivity analyses: use of a random‐effects model attenuated statistical significance (RR 1.38, 95% CI 0.99–1.92; *p* = 0.06; *I* ^2^ = 0%) (Figure S35a), and LOO analyses excluding either the DRESS trial [13] or the POET trial [41] also rendered the estimate nonsignificant (Figures S35b and S35c). Accordingly, evidence supporting an increased risk of sAEs with bDMARD withdrawal is suggestive but not definitive.

Taken together, both DMARD tapering and withdrawal did not reduce the risk of any AEs or serious AEs (Table 3).

## 4 Discussion

In this systematic review and meta‐analysis of RCTs involving RA patients with sustained remission/LDA, we found that both DMARD tapering and withdrawal were associated with increased risk of disease flare compared with full‐dose treatment, but with differences in magnitude, timing, and clinical implications. Tapering, particularly of bDMARDs, was associated with a modest increase in flare risk particularly within 9 months, whereas withdrawal led to consistently higher flare rates across all follow‐up durations (Figure 3). These distinctions have direct relevance for clinical decision making, where it is central to balance treatment burden, patient preference, flare risk, structural protection, and adverse effects.

**FIGURE 3:**
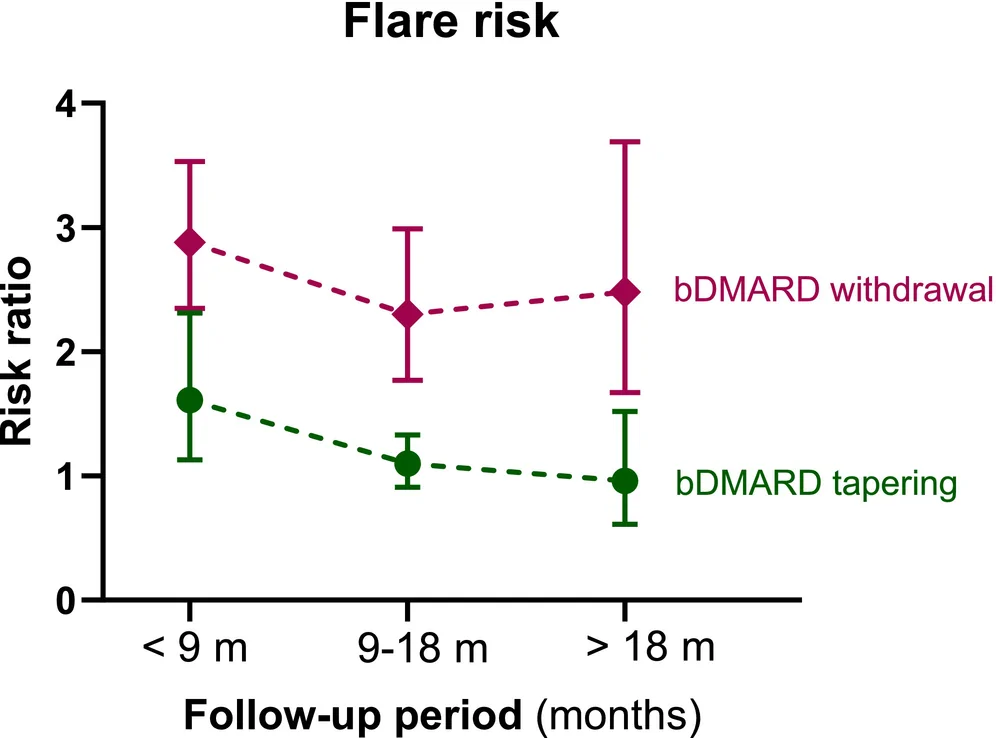
Summary of flare risk following bDMARD de‐escalation at < 9 months, 9–18 months, and > 18 months. Data presented as risk ratios (RRs) with 95% confidence intervals (CI). Risk of flare diminished with time following DMARD tapering but not after withdrawal.

Our results refine previous systematic reviews on DMARD de‐escalation. Earlier reviews [14, 15, 16, 17] synthesized highly heterogeneous patient populations, often combining individuals in sustained remission/LDA with those with temporary disease control. Since patients in temporary remission are inherently unstable and prone to early flare [49], their inclusion can inflate flare rates and bias pooled estimates, limiting the interpretability. Previous reviews also pooled studies across different follow‐up durations, which may dilute the clinical relevance of the estimates. As demonstrated in several of our analyses (e.g., Figures S1 and S9), pooling studies irrespective of follow‐up duration resulted in substantial heterogeneity and evidence of publication bias. Therefore, conclusions from analyses characterized by substantial heterogeneity and potential publication bias should be interpreted with caution. By restricting the population to patients with stable disease control, incorporating more contemporary RCTs, and stratifying analyses by DMARD class and follow‐up duration, our study provides a more comprehensive assessment of the temporal pattern of outcomes following DMARD tapering. These time‐specific estimates may be more clinically informative for the patient populations in whom current guidelines consider DMARD tapering a potential option [18, 20].

The latest EULAR recommendations state that DMARD “dose reduction may be considered” [20] with the ARCTIC REWIND studies [7, 33] providing important evidence supporting this recommendation. These studies appeared to be major contributors to the heterogeneity observed in several of our sensitivity analyses (e.g., Figures S12b, S13a and S20a). One potential explanation for this heterogeneity is the difference in the definition of flare used in the ARCTIC REWIND studies compared with other trials. However, it remains difficult to determine which definition best captures clinically relevant flares in real‐world practice [24]. Using changes in SDAI or CDAI with a proper cutoff to define flare demonstrated satisfactory performance in large independent cohorts [25], but the majority of trials included in the meta‐analysis did not use this definition. The ARCTIC REWIND studies appeared to use a relatively more lenient definition of flare, which may have been particularly appropriate for their early‐RA populations. Although these studies have provided important evidence regarding DMARD tapering, particularly in early RA, their findings represent one component of a broader and heterogeneous evidence base.

Our synthesis incorporated a wider range of studies and, importantly, examined outcomes according to different follow‐up durations, providing additional information on the evolution of disease control following DMARD tapering. Overall, we found that DMARD tapering was associated with a higher risk of flare than continued treatment (Table 2), although this difference appeared to diminish with time. The increased risk of flare may also contribute to the potential for radiographic progression observed at later stages (Table 3) [50]. In addition, reduced DMARD dosing was not associated with a significant reduction in adverse events. These findings suggest that DMARD tapering may not provide clear advantages in terms of disease control or overall safety, although the potential benefits of reducing treatment burden and costs remain clinically relevant.

Furthermore, even among patients in long‐term remission, subclinical synovitis remains detectable in some patients [51], suggesting that sustained clinical remission does not necessarily indicate complete suppression of underlying disease activity. Therefore, DMARD tapering should be considered carefully in appropriately selected patients, with individualized assessment of the potential benefits and risks. This may be particularly relevant when tapering is considered for reasons unrelated to treatment efficacy, such as treatment burden or cost. Indeed, several studies have suggested that DMARD tapering may reduce hospitalization‐related healthcare costs [52, 53, 54], although these potential economic benefits may be accompanied by a reduction in quality of life in some settings [53].

If DMARD tapering is undertaken, close monitoring of disease activity is essential to enable timely treatment adjustment in the event of disease recurrence. Ultimately, the goal should not be to broadly replace continued DMARD therapy with tapering, but rather to identify carefully selected patients in whom treatment de‐escalation can reduce treatment burden and healthcare costs while maintaining adequate disease control as well as minimizing the risk of disease progression.

In RA management, structural protection remains one of the most important considerations, particularly for patients with preexisting joint damage, erosive disease, or other established risk factors for progression, such as female sex, seropositivity, or high ESR [55]. These risk factors may help clinicians and patients jointly weigh the long‐term consequences beyond symptomatic flare when a DMARD tapering strategy is strongly considered. The significant radiographic progression following DMARD tapering found in our analyses is largely driven by the ARCTIC REWIND studies [8, 34] which are the major evidence EULAR made the above recommendations.

Regarding DMARD withdrawal, the available evidence strongly argues against its use in clinical practice, given the persistent increase in disease flares, clear radiographic progression, and no reduction in the risk of adverse events. Our findings are consistent with current guidelines, which strongly discourage the withdrawal of effective DMARD therapy.

Safety outcomes present an unexpected scenario, as neither DMARD tapering nor withdrawal was associated with a reduction in the risk of adverse events or serious adverse events. Moreover, DMARD withdrawal may increase the risk of serious adverse events, potentially due to the loss of immune control and the resurgence of systemic inflammation, such as substantial clonal expansion of T cells [56]. In this context, the risks associated with uncontrolled autoimmunity may outweigh those attributable to antirheumatic drugs themselves. Indeed, our analysis did not demonstrate a significant reduction in the risk of infection following DMARD tapering. In contrast, the risk of infection may be further increased by glucocorticoid exposure and DMARD reinitiation due to disease flares [57]. Regarding cancer risk, months of follow‐up may be insufficient to detect differences between DMARD tapering and continuation, as the development of malignancies generally requires substantially longer observation than the follow‐up durations of the included trials, rendering the effect on malignancy risk uncertain.

Our study has several strengths. First, we included patients with sustained disease control over a predefined period, enhancing the applicability of the findings. Second, we conducted subgroup analyses by DMARD class and follow‐up duration, which revealed clear temporal patterns following DMARD tapering or withdrawal. Notably, the risk of flare after DMARD tapering appeared to diminish over time under current tapering strategies. This finding underscores the importance of close monitoring, particularly during the early phase after tapering, and suggests that future trials may consider even slower, more conservative, and stepwise dose‐reduction strategies, although such approaches may be inferior to standard treatment in maintaining disease control. Third, we evaluated both structural and safety outcomes, providing a more comprehensive assessment of the benefits and risks associated with DMARD tapering or withdrawal. Several limitations should also be acknowledged. First, definitions of flare and radiographic progression were not standardized across studies, contributing to substantial heterogeneity in several analyses. Although we performed multiple sensitivity analyses to explore potential sources of heterogeneity, residual heterogeneity remained in some cases. For example, in the pooled analysis of flare risk following bDMARD withdrawal during intermediate‐term follow‐up, heterogeneity persisted despite multiple sensitivity analyses, including leave‐one‐out analyses, use of odds ratios as the effect measure, exclusion of studies with high risk of bias, and subgroup analyses according to disease state at randomization, withdrawal strategy, concomitant csDMARD use, and whether flare was explicitly defined. Notably, further leave‐one‐out analyses within the subgroup of studies that explicitly defined flare resolved the heterogeneity (Figure S19b). This finding underscores the importance of standardized and clinically meaningful definitions of flare in future trials. Nevertheless, these results should be interpreted cautiously given the residual heterogeneity and the exploratory nature of these analyses. Second, most included studies were conducted in European populations, which may limit the generalizability of our findings to other ethnic populations. Third, the findings regarding tsDMARD and all‐DMARD tapering should be interpreted cautiously because only a limited number of studies were available for these subgroup analyses. Further studies are needed to confirm these findings and better characterize the effects of these tapering strategies. Forth, the economic implications of DMARD tapering could not be directly evaluated because the included trials did not report a direct comparison of healthcare costs between continuation and de‐escalation strategies. Although some external studies suggest potential cost savings with tapering [52, 53, 54], the absence of cost‐effectiveness data and potential trade‐offs in quality of life should be considered when interpreting the clinical implications of DMARD de‐escalation. Fifth, adverse events were not uniformly defined across studies, limiting the comparability of specific safety outcomes. In particular, the limited number of reported malignancy events and the relatively short follow‐up durations preclude definitive conclusions regarding the long‐term effects of DMARD tapering on malignancy risk. Last, the lack of individual patient‐level data precluded adjustment for potential confounding factors and prevented more detailed exploration of patient‐level predictors of successful DMARD de‐escalation.

## 5 Conclusion

In RA patients with sustained disease control, continuation of standard‐dose DMARD therapy remains the preferred approach. DMARD tapering, particularly of bDMARDs, may increase the risk of disease flare and potential radiographic progression without a clear reduction in adverse events. For cost‐saving purposes, tapering may be considered in carefully selected patients with close monitoring, while further studies are needed to determine its long‐term economic benefits and effects on quality of life. In contrast, DMARD withdrawal was associated with a significant increase in disease flare and radiographic progression and should generally be avoided in clinical practice.

## Author Contributions

Z.X. and X.Z. wrote the manuscript. Z.X., X.Z., and R.K.P. designed the research. All authors performed the research and analyzed the data.

## Funding

This study was supported by the Crafoord Prize Foundation (CR25‐0001, via the Royal Swedish Academy of Sciences), the PE Lindahl Foundation (LM25‐0048, via the Royal Swedish Academy of Sciences), the Swedish Association of Rheumatology (R‐1011920), the King Gustaf V's 80th Anniversary Fund (SGI‐2024‐1067 and SGI‐2025‐1200), the Karolinska Institute Research Foundation (2024‐03940 and 2024‐02534), and the National Natural Science Foundation of China (82502185).

## Conflicts of Interest

The authors declare no conflicts of interest.

## Supporting information


**Figure S1:** Forest plot (a) and funnel plot (b) on risk of disease flares following any DMARD tapering. (a) DMARD tapering was associated with a higher risk of disease flare (pooled RR 1.56, 95% CI 1.23–1.98). (b) Funnel plot indicated possible asymmetry.
**Figure S2:** Forest plot and subgroup analysis showing the risk of disease flare associated with tapering versus maintaining standard doses of DMARDs across different drug classes. Pooled random‐effects estimates indicate a higher flare risk with DMARD tapering (overall RR = 1.56, 95% CI 1.23–1.98, *p* = 0.0002). Between‐study heterogeneity was moderate (*I* ^2^ = 64%, *τ* ^2^ = 0.11).
**Figure S3:** Forest plots of subgroup analysis showing the risk of disease flare associated with bDMARD tapering versus maintaining standard doses across different follow‐up durations. Pooled random‐effects estimates indicate that bDMARD tapering was associated with higher flare risk at short term (a) (RR = 1.61, 95% CI 1.13–2.31, *p* = 0.009), but not at intermediate term (b) (RR = 1.10, 95% CI 0.91–1.33, *p* = 0.33), or long term (c) (RR = 0.96, 95% CI 0.61–1.52, *p* = 0.88).
**Figure S4:** Funnel plots did not suggest clear asymmetry on studies concerning disease flares on bDMARD tapering at short (a) or intermediate (b) term.
**Figure S5:** Sensitivity analysis confirmed the robustness of the finding that bDMARD tapering was associated with an increased risk of flare. The corresponding ORs were 1.99 (95% CI 1.26–3.13; *p* = 0.003) in the short term (a), 1.20 (95% CI 0.87–1.65; *p* = 0.26) in the intermediate term (b), and 0.96 (95% CI 0.55–1.68; *p* = 0.88) in the long term (c).
**Figure S6:** Effect sizes of the studies (ARCTIC REWIND) on csDMARD tapering at short term (a), intermediate term(b), and long term (c).
**Figure S7:** Effect sizes of the studies on tsDMARD tapering at short term (a) and intermediate term (b).
**Figure S8:** Effect sizes of the studies on tapering all DMARDs at short term (a) and intermediate term (b).
**Figure S9:** Forest plot (a) demonstrated that DMARD withdrawal was associated with a substantially higher risk of disease flare (pooled RR 2.32, 95% CI 1.83–2.94; *p* < 0.00001); Funnel plot (b) indicated possible asymmetry.
**Figure S10:** Forest plots of subgroup analysis showing the risk of disease flare associated with DMARD withdrawal versus maintaining standard doses across different drug classes. Pooled random‐effects estimates reveal an increased flare risk upon bDMARD withdrawal (a) (RR = 2.12, 95% CI 1.70–2.64, *p* < 0.00001), but not upon csDMARD withdrawal (b) (RR = 1.62, 95% CI 0.92–2.84, *p* = 0.10). Evidence on tsDMARD withdrawal (c) or all DMARD withdrawal (d) was limited.
**Figure S11:** Funnel plots of studies investigating disease activity on withdrawing different DMARDs: (a) bDMARDs; (b) csDMARDs; (c) tsDMARDs; (d) all DMARDs.
**Figure S12:** Forest plot (a) and funnel plot (b) on risk of disease flares following bDMARD withdrawal in the short term (0–9 months). (a) bDMARD withdrawal significantly increased flare risk (RR 2.47, 95% CI 1.84–3.32; *p* < 0.00001) in the short term. (b) Funnel plot indicated small‐study effect.
**Figure S13:** Sensitivity analyses confirmed the robustness of the finding that bDMARD withdrawal was associated with short‐term increase in flare risk. (a) leave‐one‐out (LOO) analysis excluding the ARCTIC REWIND study yielded a similar effect size (RR 2.41, 95% CI 1.88–3.09; *p* < 0.00001), but largely reduced the heterogeneity (*I* ^2^ = 28%); (b) fixed‐effect model yielded consistent findings (RR 2.88, 95% CI 2.35–3.53; *p* < 0.00001).
**Figure S14:** Forest plot (a) and funnel plot (b) on risk of disease flares following bDMARD withdrawal in the intermediate term (9–18 months). (a) bDMARD withdrawal was associated with increased flare risk (RR 2.30, 95% CI 1.77–2.99; *p* < 0.00001) in the intermediate term, with high heterogeneity (*τ* ^2^ = 0.15, *I* ^2^ = 79%). (b) Funnel plot indicated small‐study effect.
**Figure S15:** Sensitivity analyses confirmed the robustness of the finding that bDMARD withdrawal was associated with intermediate‐term increase in flare risk. (a) analysis using Odd Ratio (OR) as effect size yielded a pooled OR of 4.46 (95% CI 3.28–6.07; *p* < 0.00001); (b) restricting analyses by excluding high risk‐of‐bias (RoB) studies produced similar results (RR 2.25, 95% CI 1.72–2.94).
**Figure S16:** Subgroup analysis on disease flares upon bDMARD withdrawal during the intermediate term follow‐up period (remission vs. LDA).
**Figure S17:** Subgroup analysis on disease flares upon bDMARD withdrawal during the intermediate term follow‐up period (immediate withdrawal vs. tapering to withdrawal).
**Figure S18:** Subgroup analysis on disease flares upon bDMARD withdrawal during the intermediate term follow‐up period (with csDMARDs vs. without csDMARDs).
**Figure S19:** Subgroup analysis (a) followed by leave‐one‐out (LOO) sensitivity analysis (b) on disease flares upon bDMARD withdrawal during the intermediate‐term follow‐up period (flare defined vs. flare undefined). (a) risk ratio (RRs) of the subgroups were similar to the pooled effect size; (b) LOO analysis identified one study in each subgroup significantly contributing to heterogeneity.
**Figure S20:** Forest plot (a) and leave‐one‐out (LOO) sensitivity analysis (b) on risk of disease flare following bDMARD withdrawal in the long term (> 18 months). (a) bDMARD withdrawal was associated with increased flare risk (RR 2.48, 95% CI 1.67–3.69; *p* < 0.00001), with substantial heterogeneity (*τ* ^2^ = 0.08; *I* ^2^ = 66%); (b) LOO analysis identified the ARCTIC REWIND study as the major contributor to heterogeneity.
**Figure S21:** csDMARD withdrawal was not associated with a significant increase in flare risk in the short term (RR 1.44, 95% CI 0.95–2.18; *p* = 0.08) (a) or the intermediate term (RR 1.24, 95% CI 0.84–1.85; *p* = 0.28) (b), and only one study (ARCTIC REWIND) reported the long‐term effect size (RR 3.16, 95% CI 1.92–5.20; *p* < 0.00001) (c).
**Figure S22:** Only one study reported the short‐term effect size of tsDMARD withdrawal on the risk of disease flare (RR 8.15, 95% CI 3.17–20.95; *p* < 0.0001).
**Figure S23:** The short‐term estimate (from one study) of flare risk following withdrawing all DMARDs: RR 4.63, 95% CI 2.39–8.95 (a); and the intermediate‐term pooled estimate (from two studies): RR 5.35, 95% CI 1.45–19.79 (b).
**Figure S24:** Pooled estimates of risk of radiographic progression following DMARD tapering in the intermediate term (a) and long term (b).
**Figure S25:** Forest plots of subgroup analysis showing the risk of radiographic progression associated with DMARD withdrawal versus maintaining standard doses across different follow‐up durations. Pooled fixed‐effect estimates support an increased risk of radiographic progression following DMARD withdrawal at both the intermedium term (a) (RR = 2.64, 95% CI 1.27–5.49, *p* = 0.009), and long term (b) (RR = 2.82, 95% CI 1.39 5.75, *p* = 0.004).
**Figure S26:** Sensitivity analyses confirmed the robustness of the finding that DMARD withdrawal was associated with increased risk of radiographic progression. (a) Leave‐one‐out (LOO) sensitivity analysis eliminated heterogeneity and maintained the direction and statistical significance on the risk of radiographic progression in the intermediate term (RR 3.29, 95% CI 1.47–7.36; *I* ^2^ = 0%; *p* = 0.004); (b) sensitivity analysis using a random‐effects model yielded consistent significance on the increased risk of radiographic progression in the long term (RR 2.57, 95% CI 1.26–5.27; *I* ^2^ = 0%; *p* = 0.01); (c) excluding the highest‐weight study maintained the significance on the increased risk of radiographic progression in the long term (RR 3.74, 95% CI 1.36–10.32; *I* ^2^ = 0%; *p* = 0.01).
**Figure S27:** Forest plots of subgroup analysis showing the risk of any adverse events upon DMARD tapering (a) or withdrawal (b) compared with full‐dose treatments. Pooled random‐effects estimates indicate that neither DMARD tapering (RR = 1.04, 95% CI 0.97–1.12, *p* = 0.30), nor withdrawal (RR = 1.01, 95% CI 0.92–1.10, *p* = 0.91) was associated with changes of any adverse events.
**Figure S28:** Forest plots showing that the risk of infections did not change upon DMARD tapering (pooled RR 0.95, 95% CI 0.80–1.13; *p* = 0.56) with low heterogeneity (*n* = 0.01, *I* ^2^ = 20%) (a), which was further validated by sensitivity analyses using OR as effect size (b).
**Figure S29:** Subgroup analysis revealed that tapering was not associated with an increased risk of any adverse events overall (pooled RR 1.04, 95% CI 0.97–1.12; *p* = 0.30) or in any subgroups, and no heterogeneity was detected (*τ* ^2^ = 0, *I* ^2^ = 0%).
**Figure S30:** Forest plots of subgroup analysis showing the risk of serious adverse events upon DMARD tapering (a) or withdrawal (b) compared with full‐dose treatments. Pooled fixed‐effect estimates indicate that DMARD tapering was not associated with the risk of serious adverse events (RR = 0.89, 95% CI 0.64–1.24, *p* = 0.48), but DMARD withdrawal was associated (RR = 1.31, 95% CI 1.04–1.65, *p* = 0.02).
**Figure S31:** Subgroup analysis revealed that tapering was not associated with an increased risk of serious adverse events overall (RR 0.85, 95% CI 0.61–1.20; *p* = 0.36) or in any subgroups, and no heterogeneity was detected (*I* ^2^ = 0%).
**Figure S32:** Subgroup analysis revealed that withdrawing bDMARDs (a) or csDMARDs (b) did not alter the risk of any adverse events; withdrawing bDMARDs: RR 1.00, 95% CI 0.92–1.08; *I* ^2^ = 40%; *p* = 0.91; withdrawing csDMARDs: RR 1.05, 95% CI 0.82–1.33; *I* ^2^ = 57%; *p* = 0.72. Withdrawing tsDMARDs (c) reduced the risk of any adverse events (from one study): RR 0.23, 95% CI 0.07–0.72.
**Figure S33:** Subgroup analysis indicated that bDMARD withdrawal (a) was associated with increased risk of serious adverse events (RR 1.31, 95% CI 1.02–1.70; *p* = 0.04; *I* ^2^ = 10%), whereas csDMARD withdrawal (b) (RR 0.94, 95% CI 0.34–2.57; *p* = 0.91), tsDMARD withdrawal (c) (RR 0.35, 95% CI 0.01–8.34; *p* = 0.52), or multiple DMARD withdrawal (d) (RR 1.26, 95% CI 0.58–2.73; *p* = 0.56) did not increase the risk of serious adverse events.
**Figure S34:** Subgroup analysis indicated that bDMARD withdrawal was associated with an increased risk of serious adverse events only during the intermediate‐term follow‐up (b) (RR 1.43, 95% CI 1.03 1.98; *p* = 0.03), but not in the short term (a) (RR 3.20, 95% CI 0.37–27.49; *p* = 0.29) or the long term (c) (1.28, 95% CI 0.89–1.83; *p* = 0.18).
**Figure S35:** Sensitivity analysis did not confirm the robustness of the finding that bDMARD withdrawal was associated with increased risk of serious adverse events in the intermediate term. Sensitivity analysis using a random‐effects model (a) attenuated statistical significance (RR 1.38, 95% CI 0.99–1.92; *p* = 0.06; *I* ^2^ = 0%), and leave‐one‐out (LOO) analyses excluding either the DRESS trial (b) or the POET trial (c) also rendered the estimate nonsignificant.

## Data Availability

All data generated or analyzed during this study are included in the Supporting Information.
