# Supplementary material for: Temporal Patterns of Outcomes After DMARD Tapering or Withdrawal in Rheumatoid Arthritis With Sustained Disease Control: A Systematic Review and Meta‐Analysis
Source: Clin Transl Sci. 2026 Oct 6;19(10):e70740. doi: 10.1111/cts.70740 (PMC13640517; doi:10.1111/cts.70740)
Supplement: Supplementary file 1 [file CTS-19-e70740-s001.pdf]

Supplementary materials

Temporal patterns of outcomes after DMARD tapering or withdrawal in rheumatoid arthritis with sustained disease control: a systematic review and meta-analysis

Xiaoyan Zhang [1,2], Rajan Kumar Pandey [3,4], Xi Luo [1,2], Zhongwei Xu [3,4] *

[1] Department of Anesthesiology, West China Hospital, Sichuan University, Chengdu, China

[2] West China School of Nursing, Sichuan University, Chengdu, China

[3] Medical Inflammation Research, Department of Medical Biochemistry and Biophysics, Karolinska Institute, Stockholm, Sweden

[4] Department of Orthopedic Surgery, The First Affiliated Hospital of Chongqing Medical University, Chongqing, China

* Correspondence to:

Dr. Zhongwei Xu, MD, PhD. Karolinska Institute.

Email: zhongwei.xu@ki.se or zhongwei.xu@hospital.cqmu.edu.cn

Supplementary figures

Figure S1

a

	DMARDs tapering		Standard DMARDs			Risk Ratio	
Study or Subgroup	Events	Total	Events	Total	Weight	M–H, Random, 95% CI	Year
Kjorholt 2024	18	41	16	78	7.6%	2.14 [1.23, 3.74]	2024
Emery 2023	13	50	11	50	6.1%	1.18 [0.59, 2.38]	2023
Uhrenholt 2023	20	41	7	20	6.3%	1.39 [0.71, 2.74]	2023
Wang 2023	15	42	4	41	3.8%	3.66 [1.33, 10.11]	2023
Bertrand 2022	18	32	13	34	7.9%	1.47 [0.87, 2.49]	2022
Lillegraven 2021	20	78	5	78	0.0%	4.00 [1.58, 10.12]	2021
Tascilar 2021	40	93	16	93	8.2%	2.50 [1.51, 4.14]	2021
Verhoef 2019	14	58	3	29	3.1%	2.33 [0.73, 7.48]	2019
Sanmarti 2019	24	90	9	89	6.0%	2.64 [1.30, 5.35]	2019
Takeuchi 2019	104	243	71	244	11.5%	1.47 [1.15, 1.88]	2019
Weinblatt 2017	59	126	43	84	11.0%	0.91 [0.69, 1.21]	2017
van Vollenhoven 2016	15	27	11	23	7.7%	1.16 [0.67, 2.00]	2016
El Miedany 2016	19	31	2	32	2.4%	9.81 [2.49, 38.62]	2016
Raffeiner 2015	29	159	31	164	8.8%	0.96 [0.61, 1.52]	2015
Smolen 2013	42	201	35	201	9.5%	1.20 [0.80, 1.80]	2013
**Total (95% CI)**		**1234**		**1182**	**100.0%**	**1.56 [1.23, 1.98]**	
Total events	430		272				

Heterogeneity: $Tau^2 = 0.11$; $Chi^2 = 36.27$, df = 13 (P = 0.0005); $I^2 = 64\%$
Test for overall effect: Z = 3.68 (P = 0.0002)

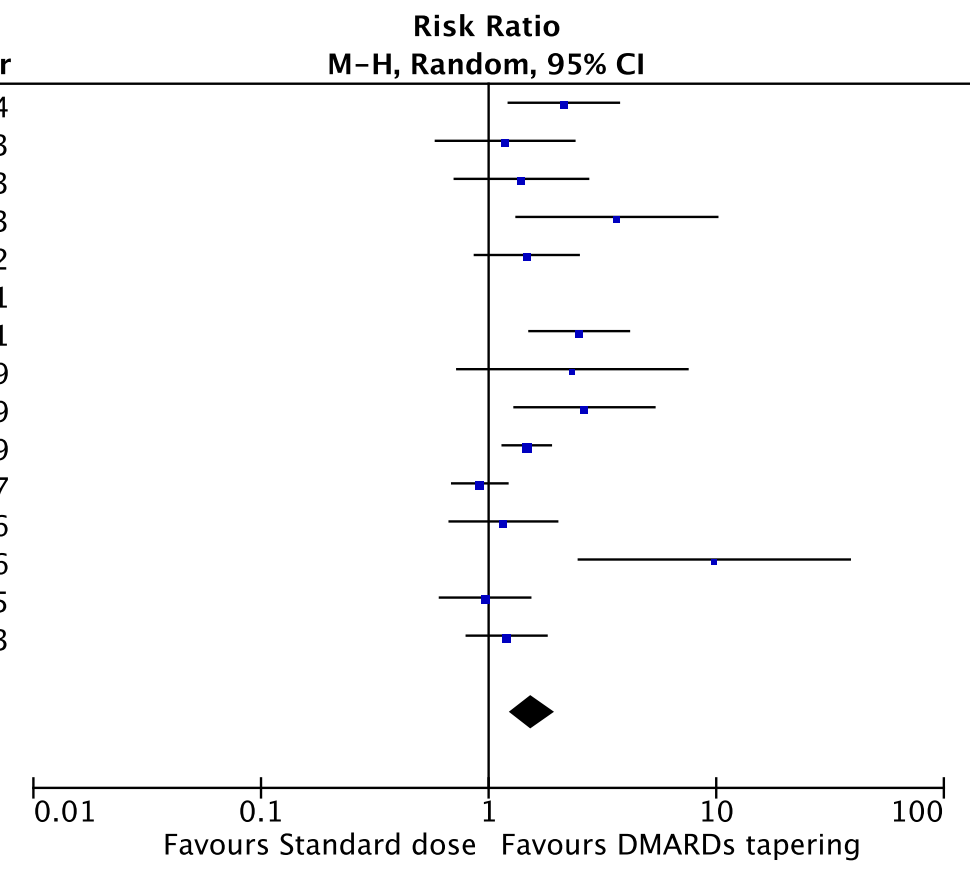

b

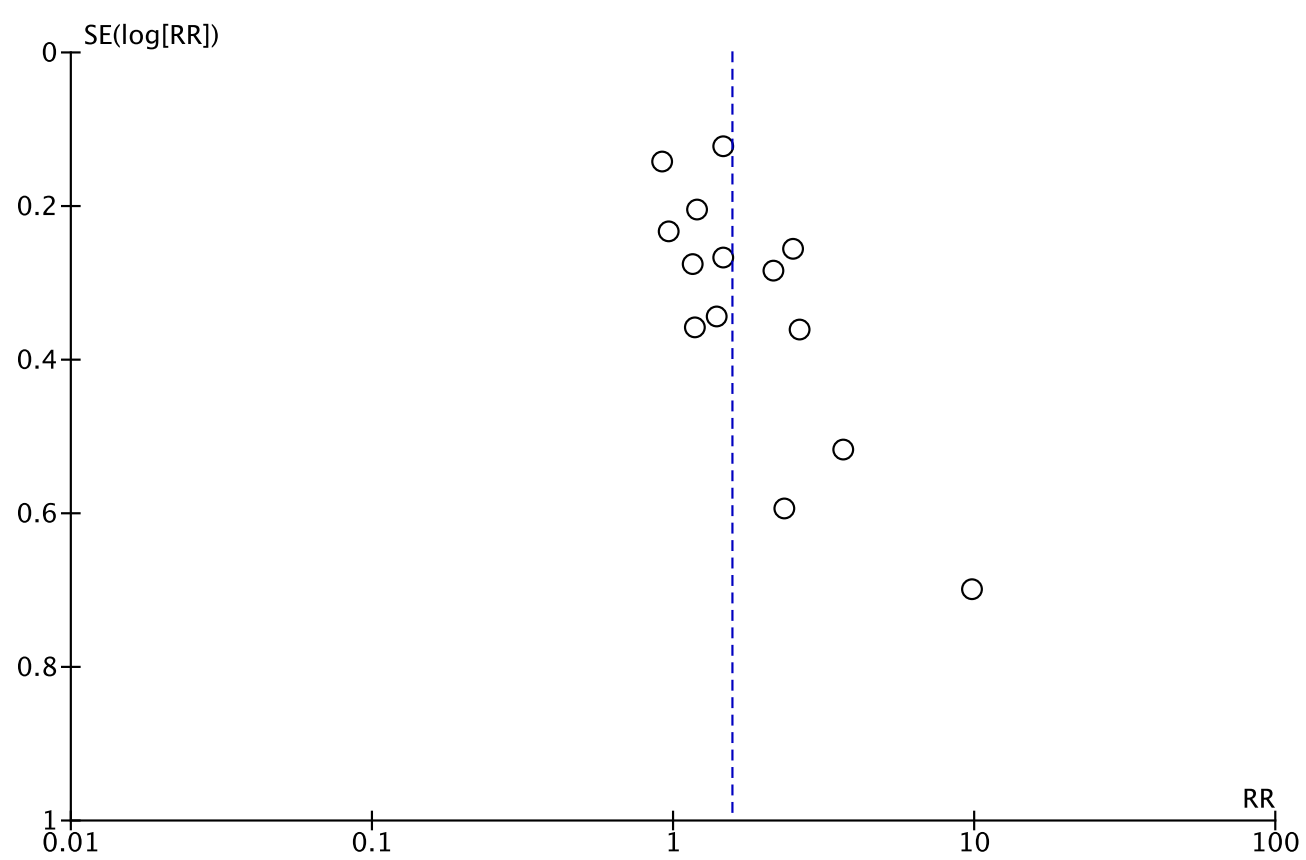

Figure S1. Forest plot (**a**) and funnel plot (**b**) on risk of disease flares following any DMARD tapering. **a**. DMARD tapering was associated with a higher risk of disease flare (pooled RR 1.56, 95% CI 1.23–1.98); **b**. Funnel plot indicated possible asymmetry.

Figure S2

Study or Subgroup	DMARDs tapering Events	DMARDs tapering Total	Standard DMARDs Events	Standard DMARDs Total	Weight	Risk Ratio M–H, Random, 95% CI	Year
**1.2.1 bDMARDs**							
Emery 2023	13	50	11	50	6.1%	1.18 [0.59, 2.38]	2023
Uhrenholt 2023	20	41	7	20	6.3%	1.39 [0.71, 2.74]	2023
Bertrand 2022	18	32	13	34	7.9%	1.47 [0.87, 2.49]	2022
Verhoef 2019	14	58	3	29	3.1%	2.33 [0.73, 7.48]	2019
Sanmarti 2019	24	90	9	89	6.0%	2.64 [1.30, 5.35]	2019
Weinblatt 2017	59	126	43	84	11.0%	0.91 [0.69, 1.21]	2017
van Vollenhoven 2016	15	27	11	23	7.7%	1.16 [0.67, 2.00]	2016
Raffeiner 2015	29	159	31	164	8.8%	0.96 [0.61, 1.52]	2015
Smolen 2013	42	201	35	201	9.5%	1.20 [0.80, 1.80]	2013
**Subtotal (95% CI)**		**784**		**694**	**66.5%**	**1.21 [0.99, 1.50]**	
Total events	234		163				
Heterogeneity: Tau² = 0.03; Chi² = 11.29, df = 8 (P = 0.19); I² = 29%							
Test for overall effect: Z = 1.84 (P = 0.07)							
**1.2.2 csDMARDs**							
Kjorholt 2024	18	41	16	78	7.6%	2.14 [1.23, 3.74]	2024
Lillegraven 2021	20	78	5	78		Not estimable	2021
**Subtotal (95% CI)**		**41**		**78**	**7.6%**	**2.14 [1.23, 3.74]**	
Total events	18		16				
Heterogeneity: Not applicable							
Test for overall effect: Z = 2.68 (P = 0.007)							
**1.2.3 tsDMARDs**							
Wang 2023	15	42	4	41	3.8%	3.66 [1.33, 10.11]	2023
Takeuchi 2019	104	243	71	244	11.5%	1.47 [1.15, 1.88]	2019
**Subtotal (95% CI)**		**285**		**285**	**15.3%**	**2.02 [0.86, 4.79]**	
Total events	119		75				
Heterogeneity: Tau² = 0.28; Chi² = 2.98, df = 1 (P = 0.08); I² = 66%							
Test for overall effect: Z = 1.61 (P = 0.11)							
**1.2.4 all DMARDs**							
Tascilar 2021	40	93	16	93	8.2%	2.50 [1.51, 4.14]	2021
El Miedany 2016	19	31	2	32	2.4%	9.81 [2.49, 38.62]	2016
**Subtotal (95% CI)**		**124**		**125**	**10.6%**	**4.28 [1.11, 16.57]**	
Total events	59		18				
Heterogeneity: Tau² = 0.72; Chi² = 3.59, df = 1 (P = 0.06); I² = 72%							
Test for overall effect: Z = 2.11 (P = 0.04)							
**Total (95% CI)**		**1234**		**1182**	**100.0%**	**1.56 [1.23, 1.98]**	
Total events	430		272				
Heterogeneity: Tau² = 0.11; Chi² = 36.27, df = 13 (P = 0.0005); I² = 64%							
Test for overall effect: Z = 3.68 (P = 0.0002)							
Test for subgroup differences: Chi² = 7.26, df = 3 (P = 0.06), I² = 58.7%							

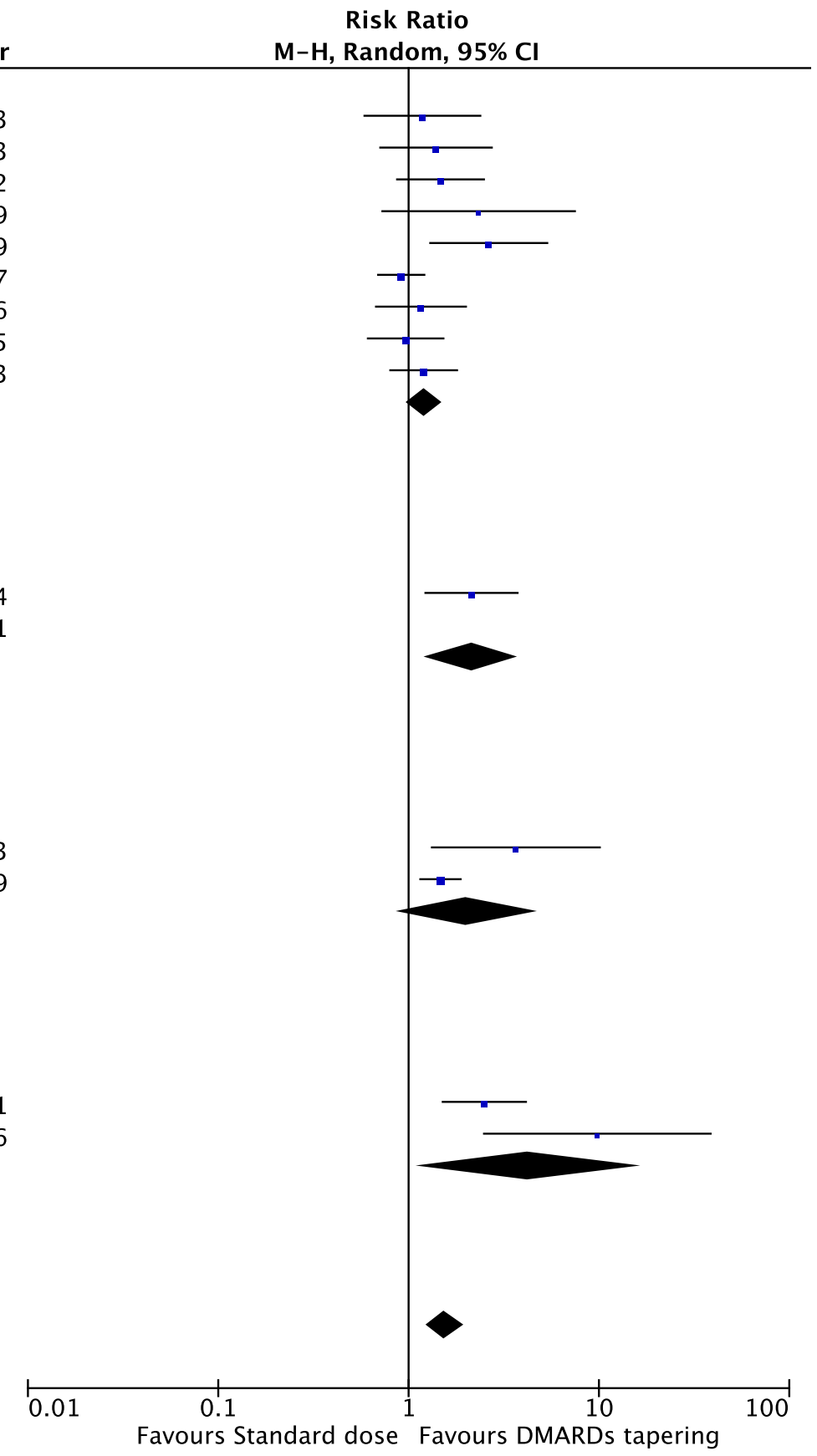

Figure S2. Forest plot and subgroup analysis showing the risk of disease flare associated with tapering versus maintaining standard doses of DMARDs across different drug classes. Pooled random-effects estimates indicate a higher flare risk with DMARD tapering (overall RR = 1.56, 95% CI 1.23–1.98, $p = 0.0002$). Between-study heterogeneity was moderate ($I^2 = 64\%$, $\tau^2 = 0.11$).

Figure S3

a

Study or Subgroup	DMARDs tapering Events	DMARDs tapering Total	Standard DMARDs Events	Standard DMARDs Total	Weight	Risk Ratio M-H, Random, 95% CI	Year
Emery 2023	13	50	11	50	24.0%	1.18 [0.59, 2.38]	2023
Bertrand 2022	13	32	8	34	21.9%	1.73 [0.83, 3.61]	2022
Verhoef 2019	14	58	3	29	9.2%	2.33 [0.73, 7.48]	2019
Sanmarti 2019	24	90	9	89	23.5%	2.64 [1.30, 5.35]	2019
van Vollenhoven 2016	10	27	8	23	21.4%	1.06 [0.51, 2.24]	2016
**Total (95% CI)**		**257**		**225**	**100.0%**	**1.61 [1.13, 2.31]**	
Total events	74		39				

Heterogeneity: $Tau^2 = 0.01$; $Chi^2 = 4.31$, df = 4 (P = 0.37); $I^2 = 7\%$
Test for overall effect: Z = 2.61 (P = 0.009)

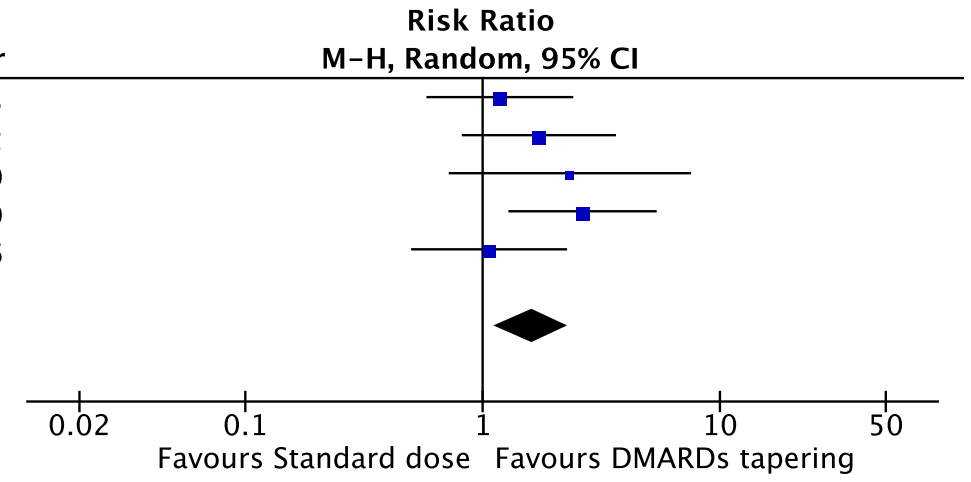

b

Study or Subgroup	DMARDs tapering Events	DMARDs tapering Total	Standard DMARDs Events	Standard DMARDs Total	Weight	Risk Ratio M-H, Random, 95% CI	Year
Uhrenholt 2023	20	41	7	20	7.8%	1.39 [0.71, 2.74]	2023
Bertrand 2022	18	32	13	34	12.9%	1.47 [0.87, 2.49]	2022
Weinblatt 2017	59	126	43	84	45.5%	0.91 [0.69, 1.21]	2017
van Vollenhoven 2016	15	27	11	23	12.0%	1.16 [0.67, 2.00]	2016
Smolen 2013	42	201	35	201	21.8%	1.20 [0.80, 1.80]	2013
**Total (95% CI)**		**427**		**362**	**100.0%**	**1.10 [0.91, 1.33]**	
Total events	154		109				

Heterogeneity: $Tau^2 = 0.00$; $Chi^2 = 3.57$, df = 4 (P = 0.47); $I^2 = 0\%$
Test for overall effect: Z = 0.97 (P = 0.33)

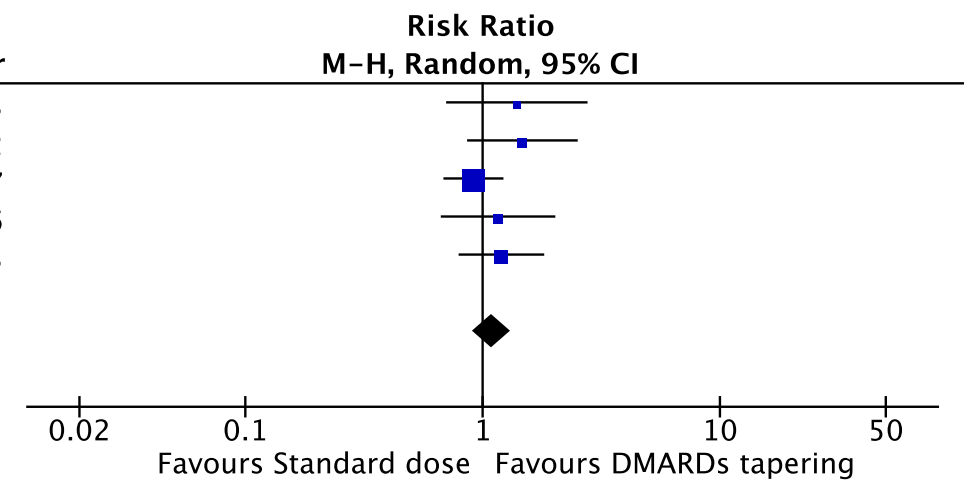

c

Study or Subgroup	DMARDs tapering Events	DMARDs tapering Total	Standard DMARDs Events	Standard DMARDs Total	Weight	Risk Ratio M-H, Random, 95% CI	Year
Raffeiner 2015	29	159	31	164	100.0%	0.96 [0.61, 1.52]	2015
**Total (95% CI)**		**159**		**164**	**100.0%**	**0.96 [0.61, 1.52]**	
Total events	29		31				

Heterogeneity: Not applicable
Test for overall effect: Z = 0.15 (P = 0.88)

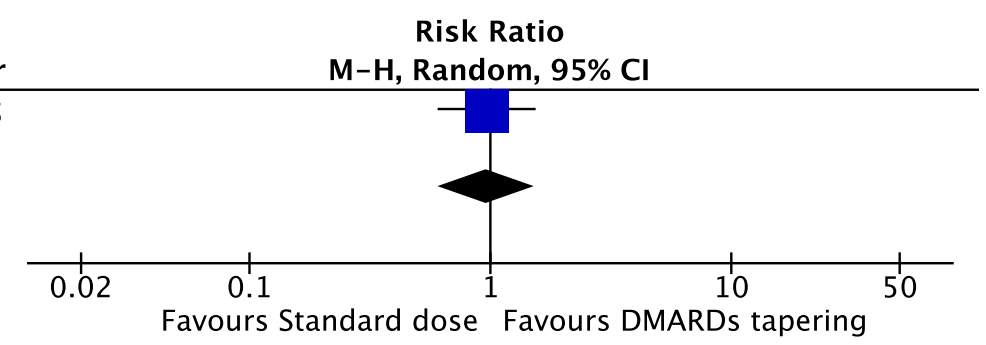

Figure S3. Forest plots of subgroup analysis showing the risk of disease flare associated with bDMARD tapering versus maintaining standard doses across different follow-up durations. Pooled random-effects estimates indicate that bDMARD tapering was associated with higher flare risk at short term (**a**) (RR = 1.61, 95% CI 1.13–2.31, $p = 0.009$), but not at intermediate term (**b**) (RR = 1.10, 95% CI 0.91–1.33, $p = 0.33$), or long term (**c**) (RR = 0.96, 95% CI 0.61–1.52, $p = 0.88$).

Figure S4

a **b**

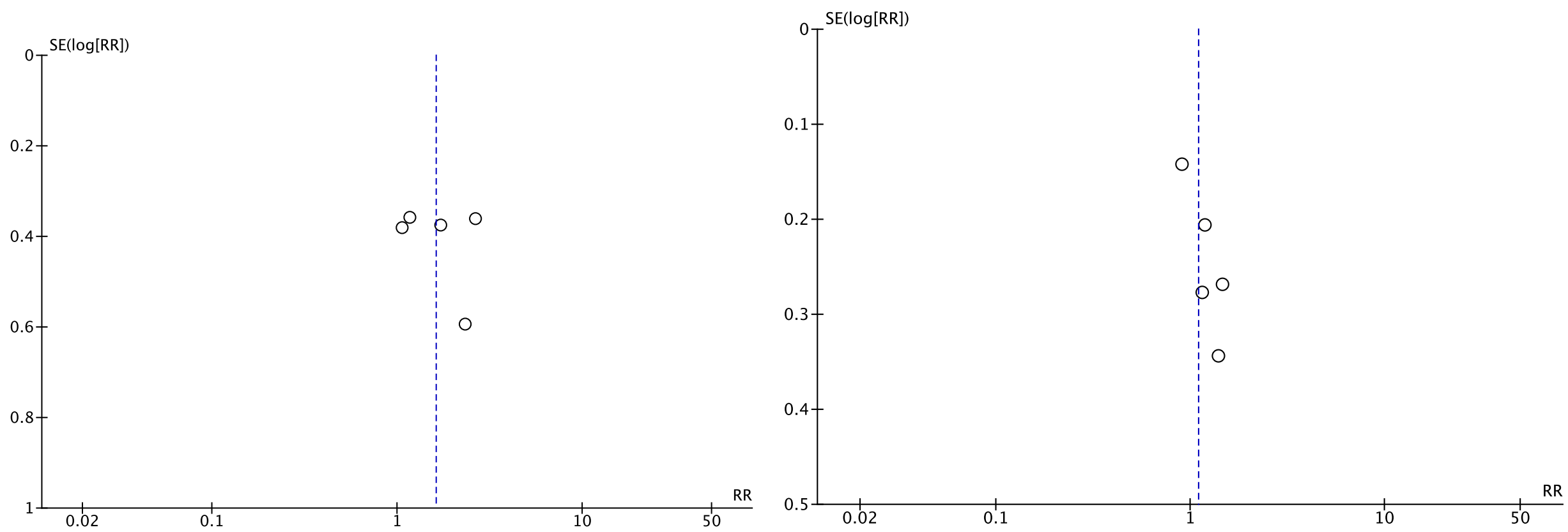

Figure S4. Funnel plots did not suggest clear asymmetry on studies concerning disease flares on bDMARD tapering at short (**a**) or intermediate (**b**) term.

Figure S5

a

Study or Subgroup	DMARDs tapering Events	DMARDs tapering Total	Standard DMARDs Events	Standard DMARDs Total	Weight	Odds Ratio M–H, Random, 95% CI	Year
Emery 2023	13	50	11	50	24.5%	1.25 [0.50, 3.13]	2023
Bertrand 2022	13	32	8	34	18.5%	2.22 [0.77, 6.42]	2022
Verhoef 2019	14	58	3	29	11.6%	2.76 [0.72, 10.51]	2019
Sanmarti 2019	24	90	9	89	30.0%	3.23 [1.41, 7.43]	2019
van Vollenhoven 2016	10	27	8	23	15.4%	1.10 [0.35, 3.52]	2016
**Total (95% CI)**		**257**		**225**	**100.0%**	**1.99 [1.26, 3.13]**	
Total events	74		39				

Heterogeneity: Tau^2 = 0.00; Chi^2 = 3.57, df = 4 (P = 0.47); I^2 = 0%
Test for overall effect: Z = 2.95 (P = 0.003)

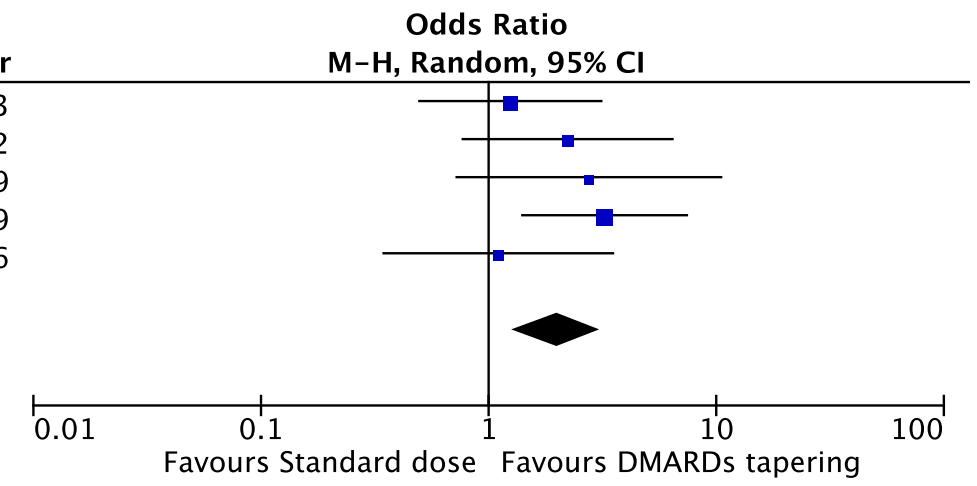

b

Study or Subgroup	DMARDs tapering Events	DMARDs tapering Total	Standard DMARDs Events	Standard DMARDs Total	Weight	Odds Ratio M–H, Random, 95% CI	Year
Uhrenholt 2023	20	41	7	20	8.2%	1.77 [0.59, 5.34]	2023
Bertrand 2022	18	32	13	34	10.4%	2.08 [0.78, 5.55]	2022
Weinblatt 2017	59	126	43	84	32.9%	0.84 [0.48, 1.46]	2017
van Vollenhoven 2016	15	27	11	23	8.1%	1.36 [0.45, 4.16]	2016
Smolen 2013	42	201	35	201	40.4%	1.25 [0.76, 2.06]	2013
**Total (95% CI)**		**427**		**362**	**100.0%**	**1.20 [0.87, 1.65]**	
Total events	154		109				

Heterogeneity: Tau^2 = 0.00; Chi^2 = 3.35, df = 4 (P = 0.50); I^2 = 0%
Test for overall effect: Z = 1.12 (P = 0.26)

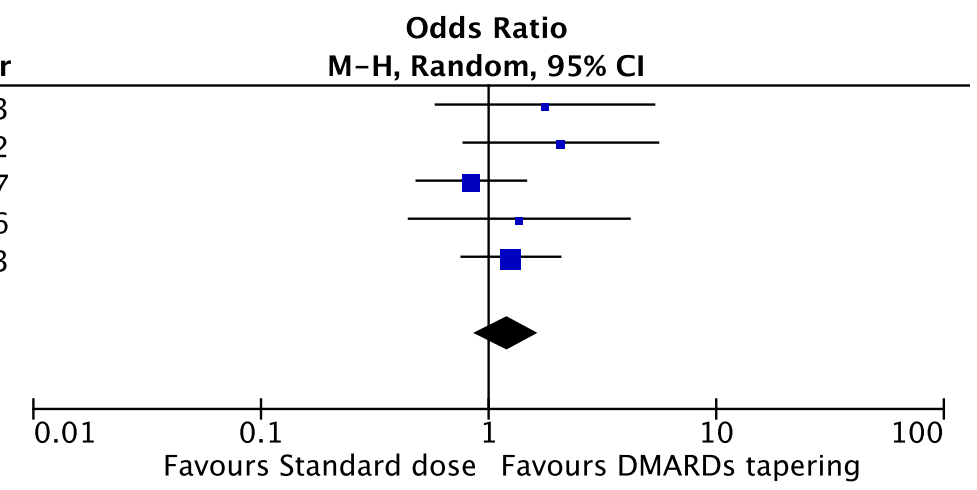

c

Study or Subgroup	DMARDs tapering Events	DMARDs tapering Total	Standard DMARDs Events	Standard DMARDs Total	Weight	Odds Ratio M–H, Random, 95% CI	Year
Raffeiner 2015	29	159	31	164	100.0%	0.96 [0.55, 1.68]	2015
**Total (95% CI)**		**159**		**164**	**100.0%**	**0.96 [0.55, 1.68]**	
Total events	29		31				

Heterogeneity: Not applicable
Test for overall effect: Z = 0.15 (P = 0.88)

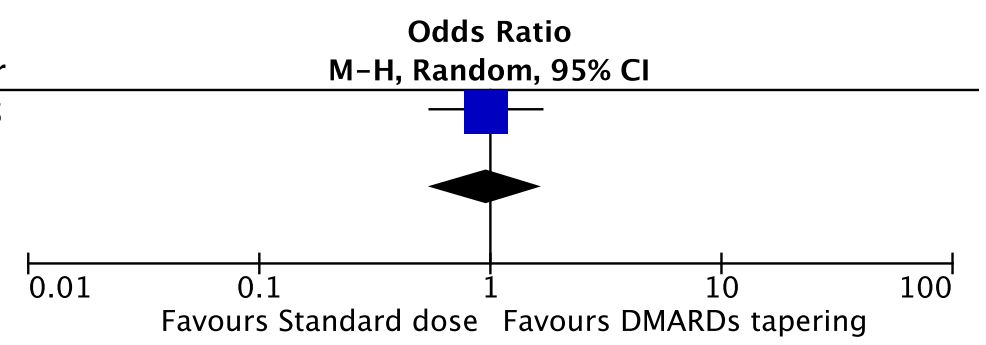

Figure S5. Sensitivity analysis confirmed the robustness of the finding that bDMARD tapering was associated with an increased risk of flare. The corresponding ORs were 1.99 (95% CI 1.26–3.13; p = 0.003) in the short term (**a**), 1.20 (95% CI 0.87–1.65; p = 0.26) in the intermediate term (**b**), and 0.96 (95% CI 0.55–1.68; p = 0.88) in the long term (**c**).

Figure S6

a

Study or Subgroup	DMARDs tapering Events	Total	Standard DMARDs Events	Total	Weight	Risk Ratio M-H, Random, 95% CI	Year
Lillegraven 2021	11	77	2	78	100.0%	5.57 [1.28, 24.31]	2021
**Total (95% CI)**		**77**		**78**	**100.0%**	**5.57 [1.28, 24.31]**	
Total events	11		2				
Heterogeneity: Not applicable							
Test for overall effect: Z = 2.28 (P = 0.02)							

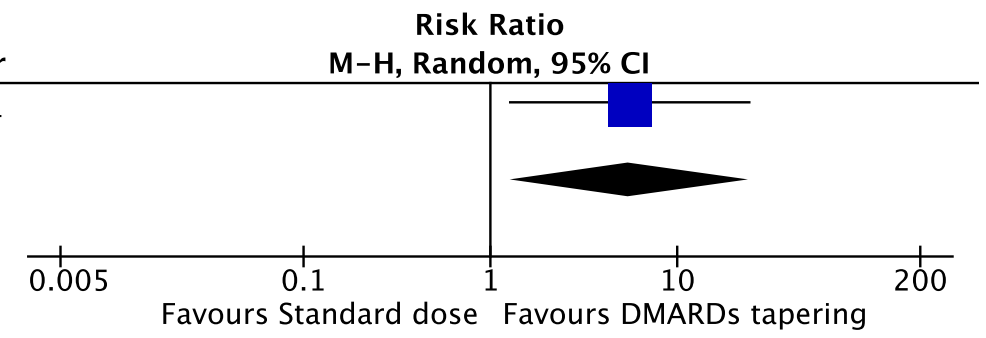

b

Study or Subgroup	DMARDs tapering Events	Total	Standard DMARDs Events	Total	Weight	Risk Ratio M-H, Random, 95% CI	Year
Lillegraven 2021	20	78	5	78	100.0%	4.00 [1.58, 10.12]	2021
**Total (95% CI)**		**78**		**78**	**100.0%**	**4.00 [1.58, 10.12]**	
Total events	20		5				
Heterogeneity: Not applicable							
Test for overall effect: Z = 2.93 (P = 0.003)							

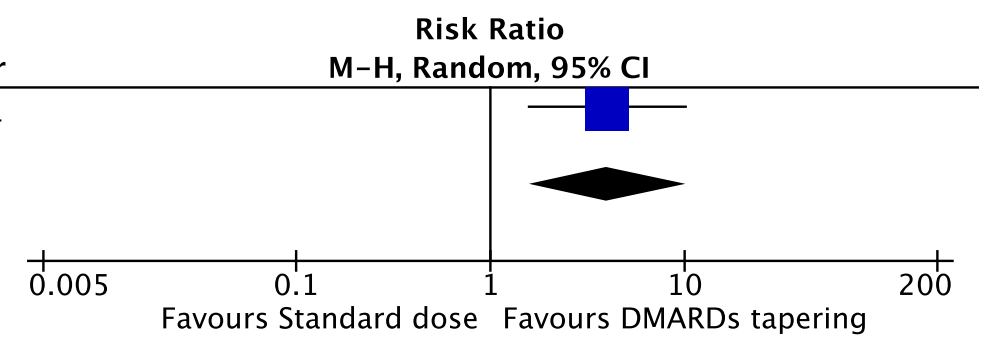

c

Study or Subgroup	DMARDs tapering Events	Total	Standard DMARDs Events	Total	Weight	Risk Ratio M-H, Random, 95% CI	Year
Kjorholt 2024	18	41	16	78	100.0%	2.14 [1.23, 3.74]	2024
**Total (95% CI)**		**41**		**78**	**100.0%**	**2.14 [1.23, 3.74]**	
Total events	18		16				
Heterogeneity: Not applicable							
Test for overall effect: Z = 2.68 (P = 0.007)							

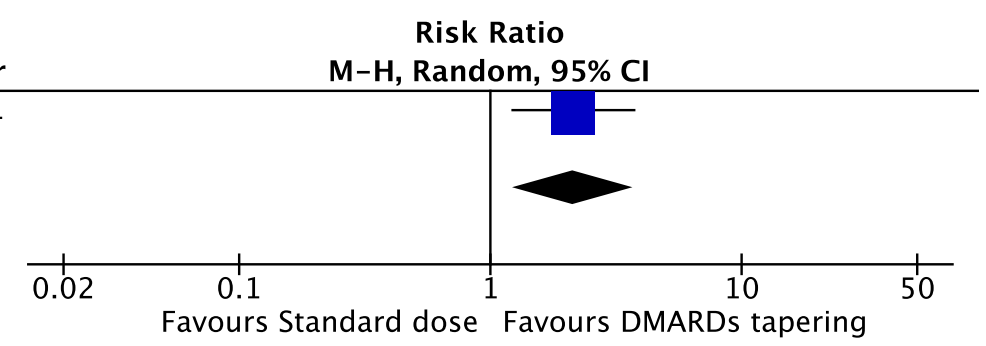

Figure S6. Effect sizes of the studies (ARCTIC REWIND) on csDMARD tapering at short term (**a**), intermediate term(**b**), and long term (**c**).

Figure S7

a

Study or Subgroup	DMARDs tapering Events	DMARDs tapering Total	Standard DMARDs Events	Standard DMARDs Total	Weight	Risk Ratio M-H, Random, 95% CI	Year
Wang 2023	15	42	4	41	30.2%	3.66 [1.33, 10.11]	2023
Takeuchi 2019	85	243	51	244	69.8%	1.67 [1.24, 2.26]	2019
**Total (95% CI)**		**285**		**285**	**100.0%**	**2.12 [1.04, 4.30]**	
Total events	100		55				

Heterogeneity: $Tau^2 = 0.16$; $Chi^2 = 2.12$, df = 1 (P = 0.15); $I^2 = 53\%$
Test for overall effect: Z = 2.08 (P = 0.04)

Risk Ratio
M-H, Random, 95% CI

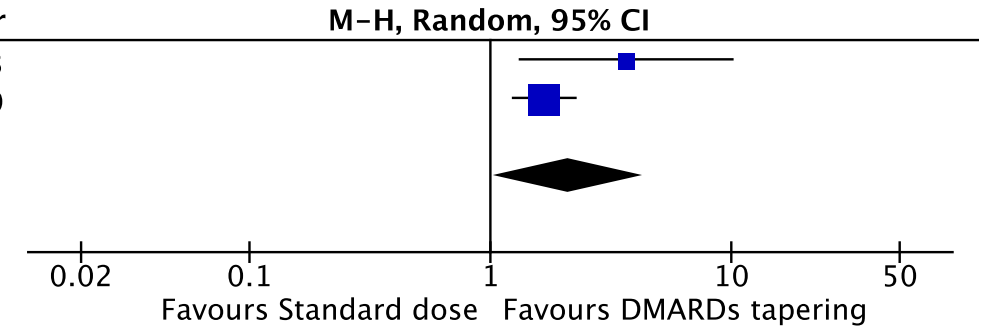

b

Study or Subgroup	DMARDs tapering Events	DMARDs tapering Total	Standard DMARDs Events	Standard DMARDs Total	Weight	Risk Ratio M-H, Random, 95% CI	Year
Takeuchi 2019	104	243	71	244	100.0%	1.47 [1.15, 1.88]	2019
**Total (95% CI)**		**243**		**244**	**100.0%**	**1.47 [1.15, 1.88]**	
Total events	104		71				

Heterogeneity: Not applicable
Test for overall effect: Z = 3.10 (P = 0.002)

Risk Ratio
M-H, Random, 95% CI

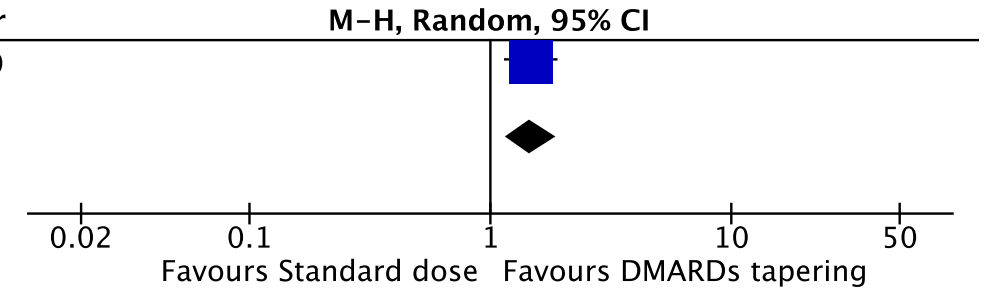

Figure S7. Effect sizes of the studies on tsDMARD tapering at short term (**a**) and intermediate term(**b**).

Figure S8

a

Study or Subgroup	DMARDs tapering Events	DMARDs tapering Total	Standard DMARDs Events	Standard DMARDs Total	Weight	Risk Ratio M-H, Random, 95% CI	Year
Tascilar 2021	28	93	9	93	100.0%	3.11 [1.55, 6.23]	2021
**Total (95% CI)**		**93**		**93**	**100.0%**	**3.11 [1.55, 6.23]**	
Total events	28		9				

Heterogeneity: Not applicable
Test for overall effect: Z = 3.21 (P = 0.001)

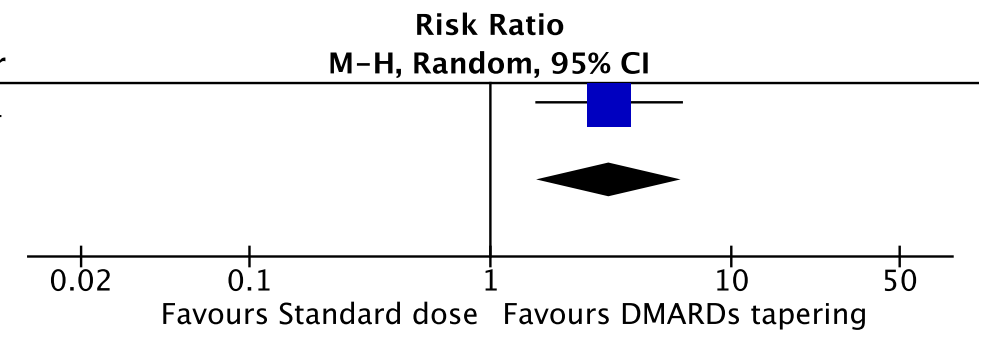

b

Study or Subgroup	DMARDs tapering Events	DMARDs tapering Total	Standard DMARDs Events	Standard DMARDs Total	Weight	Risk Ratio M-H, Random, 95% CI	Year
El Miedany 2016	19	31	2	32	39.4%	9.81 [2.49, 38.62]	2016
Tascilar 2021	40	93	16	93	60.6%	2.50 [1.51, 4.14]	2021
**Total (95% CI)**		**124**		**125**	**100.0%**	**4.28 [1.11, 16.57]**	
Total events	59		18				

Heterogeneity: $Tau^2 = 0.72$; $Chi^2 = 3.59$, df = 1 (P = 0.06); $I^2 = 72\%$
Test for overall effect: Z = 2.11 (P = 0.04)

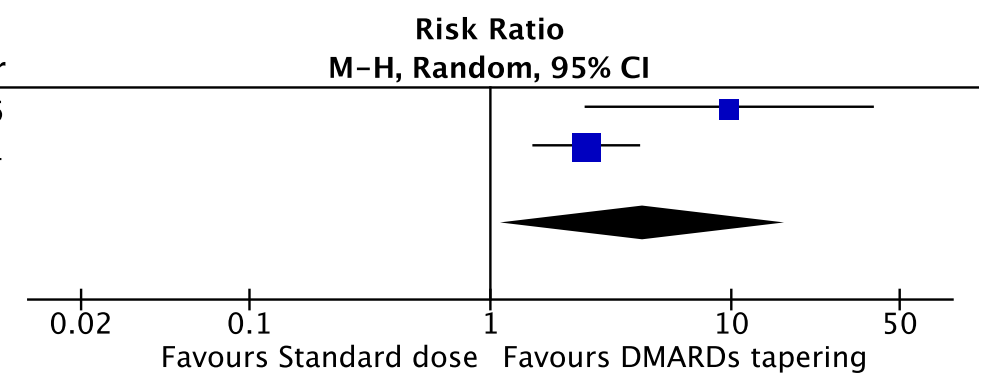

Figure S8. Effect sizes of the studies on tapering all DMARDs at short term (**a**) and intermediate term(**b**).

Figure S9

a

Study or Subgroup	DMARDs withdrawal Events	DMARDs withdrawal Total	Standard DMARDs Events	Standard DMARDs Total	Weight	Risk Ratio M-H, Random, 95% CI	Year
Kjorholt 2025	35	47	7	45	4.7%	4.79 [2.37, 9.65]	2025
Ward 2025	32	73	5	34	4.0%	2.98 [1.27, 6.98]	2025
Asai 2025	7	43	6	41	3.3%	1.11 [0.41, 3.03]	2025
Kedra 2024	76	113	33	115	7.0%	2.34 [1.71, 3.21]	2024
Kjorholt 2024	24	37	16	78	5.9%	3.16 [1.92, 5.20]	2024
Lillegraven 2023	28	47	4	45	0.0%	6.70 [2.55, 17.58]	2023
Wang 2023	31	39	4	41	3.5%	8.15 [3.17, 20.95]	2023
Emery 2023	20	47	13	50	5.5%	1.64 [0.92, 2.90]	2023
Curtis 2021	51	101	24	51	6.8%	1.07 [0.76, 1.52]	2021
Tascilar 2021	53	96	16	93	6.0%	3.21 [1.98, 5.19]	2021
Bouman 2017	96	115	25	57	7.1%	1.90 [1.40, 2.58]	2017
Weinblatt 2017	48	79	43	84	7.3%	1.19 [0.90, 1.56]	2017
Moghadam 2016	272	531	52	286	7.3%	2.82 [2.17, 3.65]	2016
Fautrel 2016	49	64	34	73	7.2%	1.64 [1.24, 2.18]	2016
El Miedany 2016	24	32	2	32	2.2%	12.00 [3.09, 46.60]	2016
van Vollenhoven 2016	20	23	11	23	6.2%	1.82 [1.15, 2.87]	2016
Chatzidionysiou 2016	12	15	8	16	5.6%	1.60 [0.92, 2.78]	2016
Yamanaka 2016	13	28	4	32	3.3%	3.71 [1.37, 10.09]	2016
van Herwaarden 2015	88	121	16	59	0.0%	2.68 [1.74, 4.13]	2015
Smolen 2013	113	197	35	201	7.0%	3.29 [2.38, 4.56]	2013
**Total (95% CI)**		**1680**		**1352**	**100.0%**	**2.32 [1.83, 2.94]**	
Total events	976		338				

Heterogeneity: Tau^2 = 0.18; Chi^2 = 84.40, df = 17 (P < 0.00001); I^2 = 80%
Test for overall effect: Z = 6.94 (P < 0.00001)

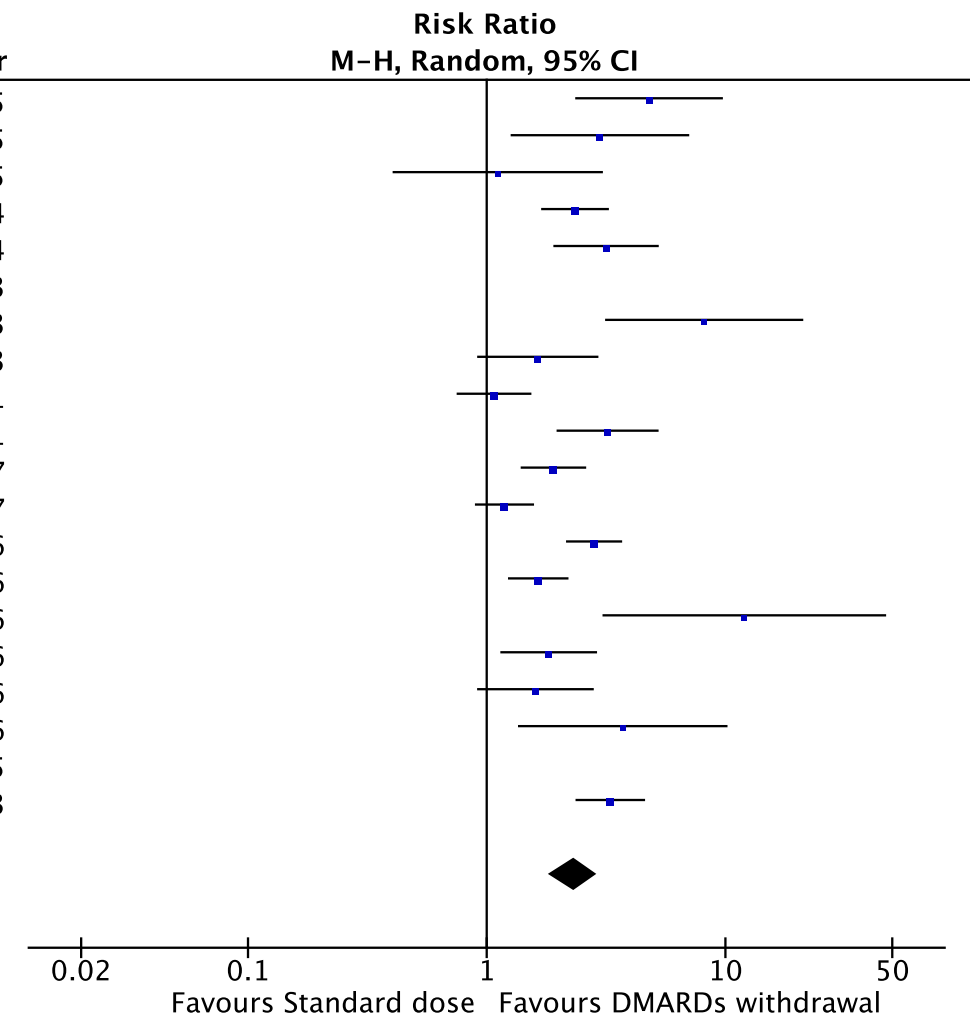

b

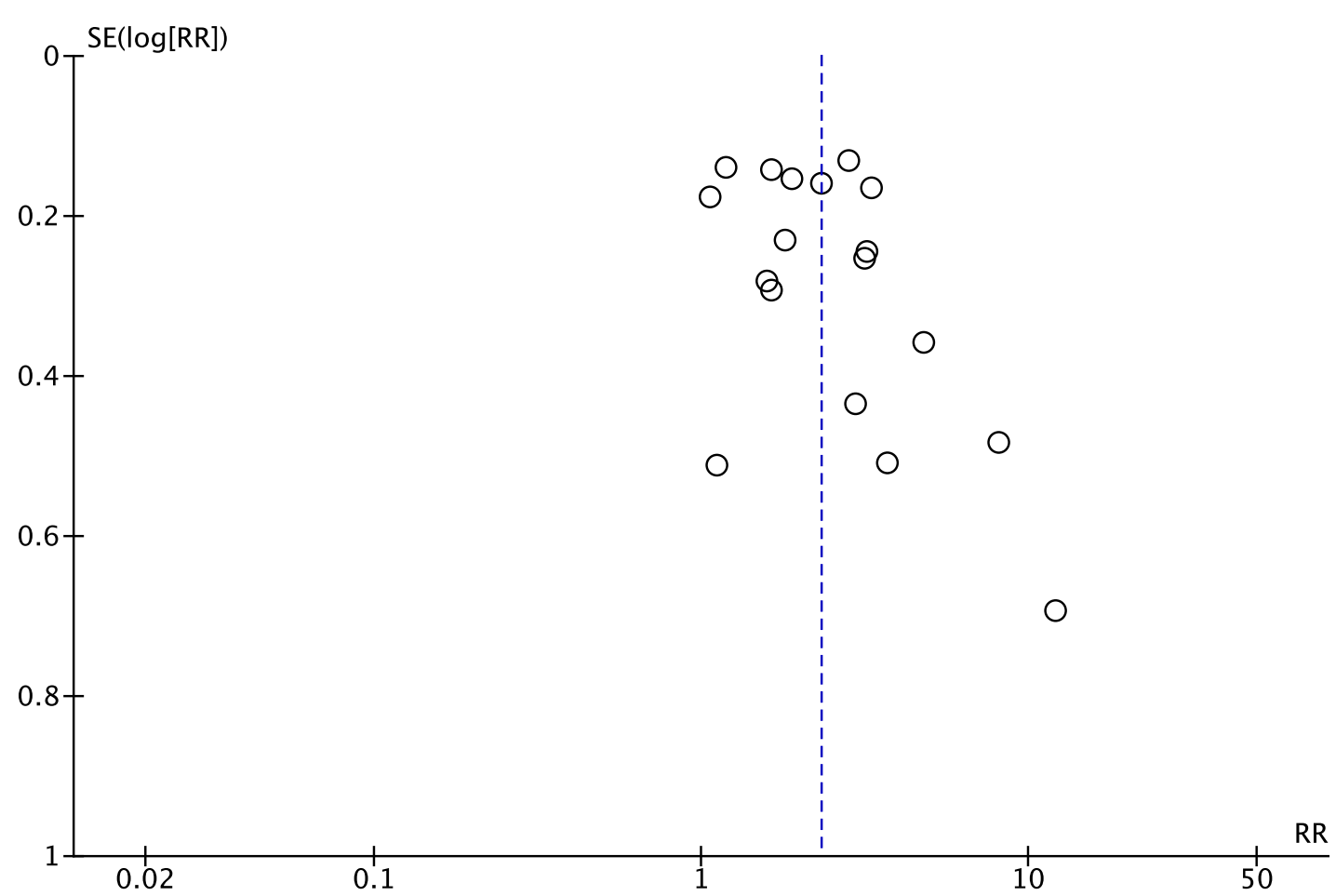

Figure S9. Forest plot (**a**) demonstrated that DMARD withdrawal was associated with a substantially higher risk of disease flare (pooled RR 2.32, 95% CI 1.83–2.94; $p < 0.00001$); Funnel plot (**b**) indicated possible asymmetry.

Figure S10

a

Study or Subgroup	DMARDs withdrawal Events	DMARDs withdrawal Total	Standard DMARDs Events	Standard DMARDs Total	Weight	Risk Ratio M-H, Random, 95% CI	Year
Kjorholt 2025	35	47	7	45	5.3%	4.79 [2.37, 9.65]	2025
Ward 2025	32	73	5	34	4.2%	2.98 [1.27, 6.98]	2025
Kedra 2024	76	113	33	115	9.2%	2.34 [1.71, 3.21]	2024
Lillegraven 2023	28	47	4	45		Not estimable	2023
Emery 2023	26	50	13	50	6.8%	2.00 [1.17, 3.43]	2023
Curtis 2021	72	101	24	51	9.2%	1.51 [1.10, 2.08]	2021
Bouman 2017	96	115	25	57	9.3%	1.90 [1.40, 2.58]	2017
Weinblatt 2017	48	79	43	84	9.7%	1.19 [0.90, 1.56]	2017
Moghadam 2016	272	531	52	286	9.8%	2.82 [2.17, 3.65]	2016
Fautrel 2016	49	64	34	73	9.6%	1.64 [1.24, 2.18]	2016
van Vollenhoven 2016	20	23	11	23	7.7%	1.82 [1.15, 2.87]	2016
Chatzidionysiou 2016	12	15	8	16	6.6%	1.60 [0.92, 2.78]	2016
Yamanaka 2016	13	28	4	32	3.4%	3.71 [1.37, 10.09]	2016
van Herwaarden 2015	88	121	16	59		Not estimable	2015
Smolen 2013	113	197	35	201	9.1%	3.29 [2.38, 4.56]	2013
**Total (95% CI)**		**1436**		**1067**	**100.0%**	**2.12 [1.70, 2.64]**	
Total events	864		294				

Heterogeneity: $Tau^2 = 0.11$; $Chi^2 = 48.90$, df = 12 (P < 0.00001); $I^2 = 75\%$
Test for overall effect: Z = 6.67 (P < 0.00001)

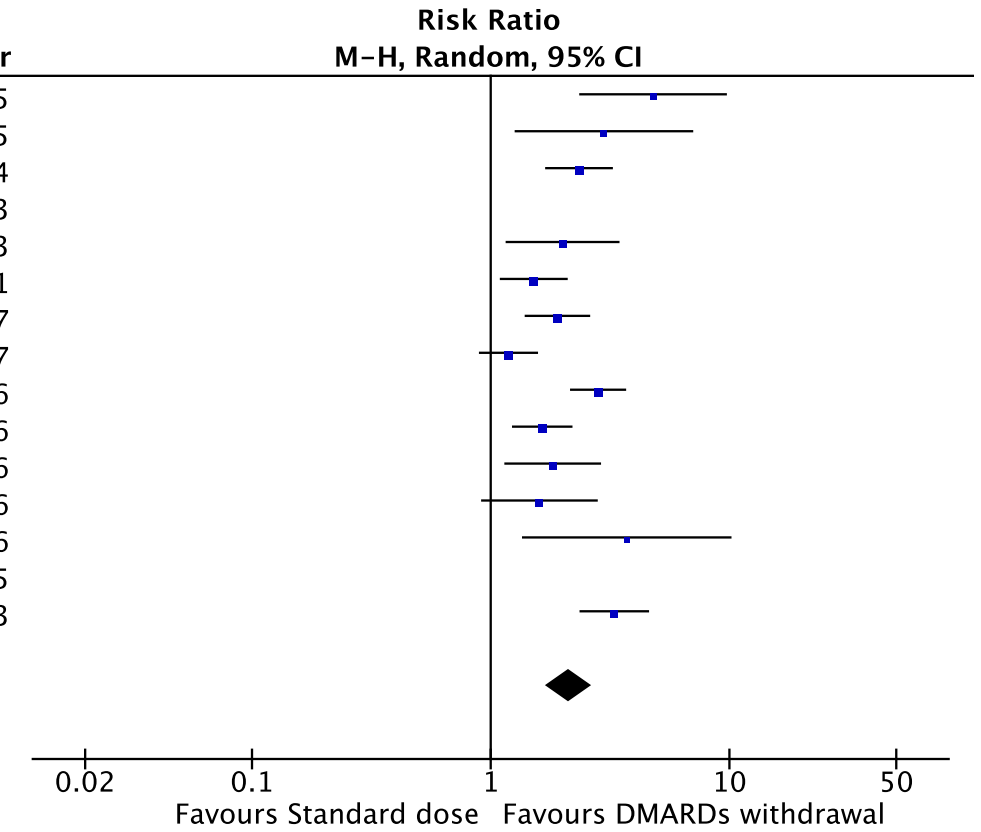

b

Study or Subgroup	DMARDs withdrawal Events	DMARDs withdrawal Total	Standard DMARDs Events	Standard DMARDs Total	Weight	Risk Ratio M-H, Random, 95% CI	Year
Asai 2025	7	43	6	41	16.5%	1.11 [0.41, 3.03]	2025
Kjorholt 2024	24	37	16	78	27.3%	3.16 [1.92, 5.20]	2024
Emery 2023	20	47	13	50	25.5%	1.64 [0.92, 2.90]	2023
Curtis 2021	51	101	24	51	30.6%	1.07 [0.76, 1.52]	2021
**Total (95% CI)**		**228**		**220**	**100.0%**	**1.62 [0.92, 2.84]**	
Total events	102		59				

Heterogeneity: $Tau^2 = 0.24$; $Chi^2 = 12.61$, df = 3 (P = 0.006); $I^2 = 76\%$
Test for overall effect: Z = 1.67 (P = 0.10)

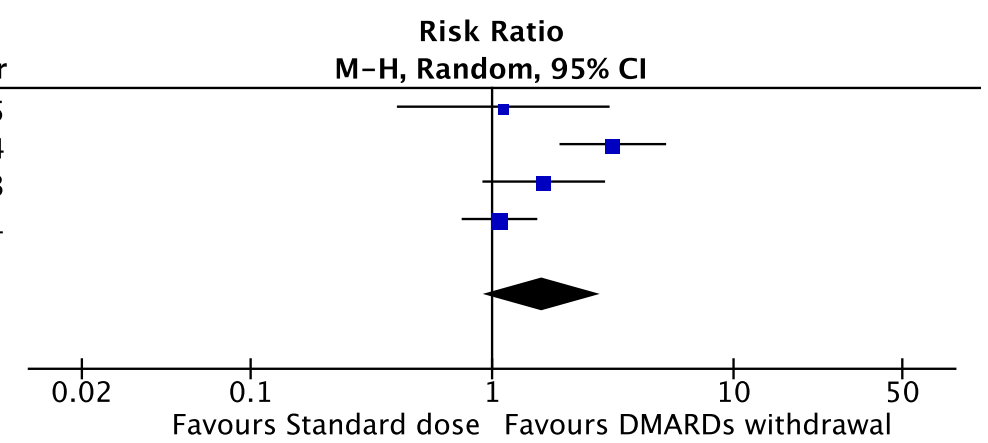

c

Study or Subgroup	DMARDs withdrawal Events	DMARDs withdrawal Total	Standard DMARDs Events	Standard DMARDs Total	Weight	Risk Ratio M-H, Random, 95% CI	Year
Wang 2023	31	39	4	41	100.0%	8.15 [3.17, 20.95]	2023
**Total (95% CI)**		**39**		**41**	**100.0%**	**8.15 [3.17, 20.95]**	
Total events	31		4				

Heterogeneity: Not applicable
Test for overall effect: Z = 4.35 (P < 0.0001)

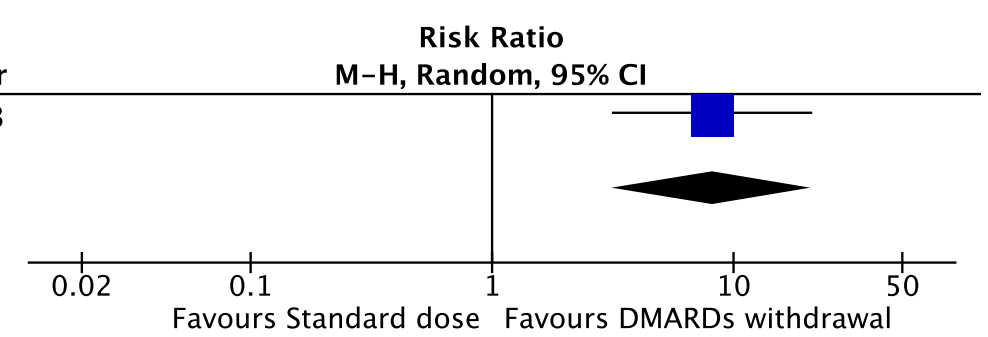

d

Study or Subgroup	DMARDs withdrawal Events	DMARDs withdrawal Total	Standard DMARDs Events	Standard DMARDs Total	Weight	Risk Ratio M-H, Random, 95% CI	Year
Tascilar 2021	53	96	16	93	61.2%	3.21 [1.98, 5.19]	2021
El Miedany 2016	24	32	2	32	38.8%	12.00 [3.09, 46.60]	2016
**Total (95% CI)**		**128**		**125**	**100.0%**	**5.35 [1.45, 19.79]**	
Total events	77		18				

Heterogeneity: $Tau^2 = 0.67$; $Chi^2 = 3.47$, df = 1 (P = 0.06); $I^2 = 71\%$
Test for overall effect: Z = 2.52 (P = 0.01)

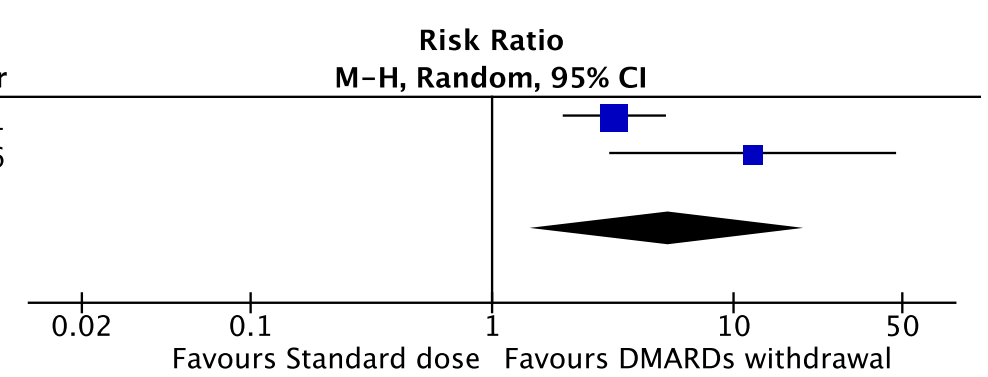

Figure S10. Forest plots of subgroup analysis showing the risk of disease flare associated with DMARD withdrawal versus maintaining standard doses across different drug classes. Pooled random-effects estimates reveal an increased flare risk upon bDMARD withdrawal (**a**) (RR = 2.12, 95% CI 1.70–2.64, $p < 0.00001$), but not upon csDMARD withdrawal (**b**) (RR = 1.62, 95% CI 0.92–2.84, $p = 0.10$). Evidence on tsDMARD withdrawal (**c**) or all DMARD withdrawal (**d**) was limited.

Figure S11

a

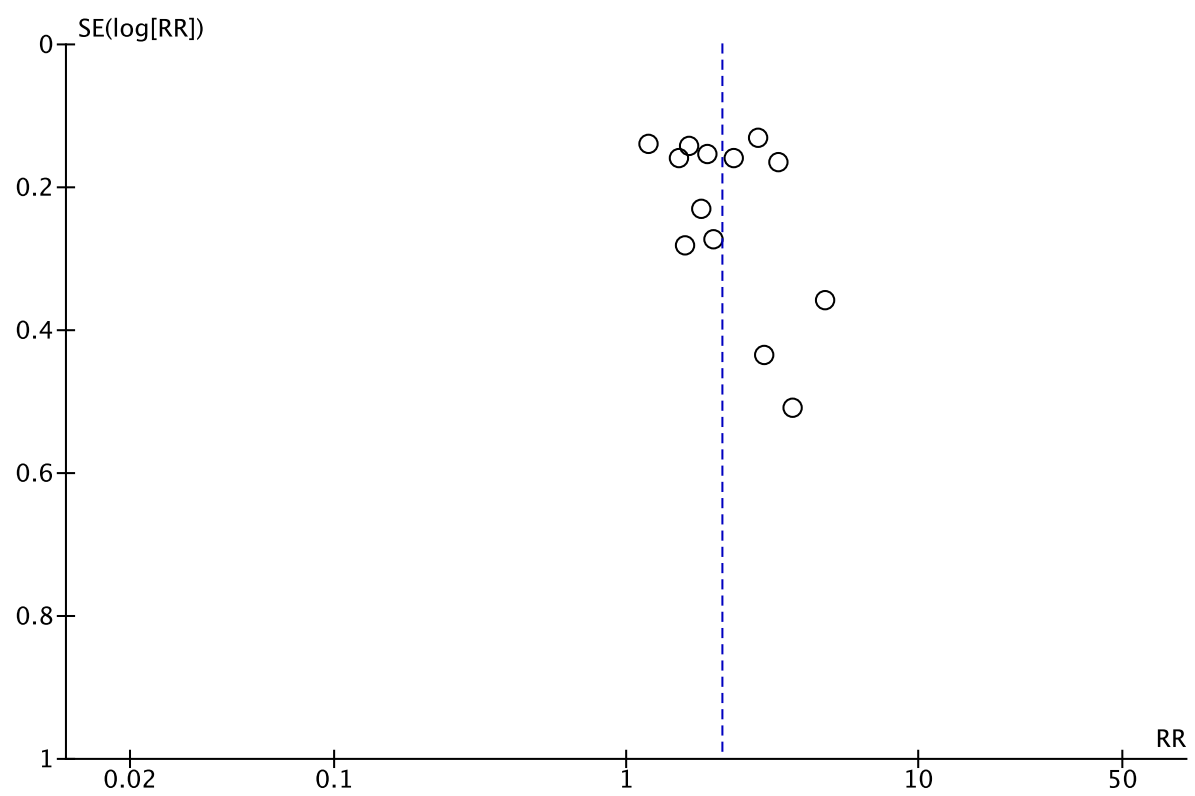

b

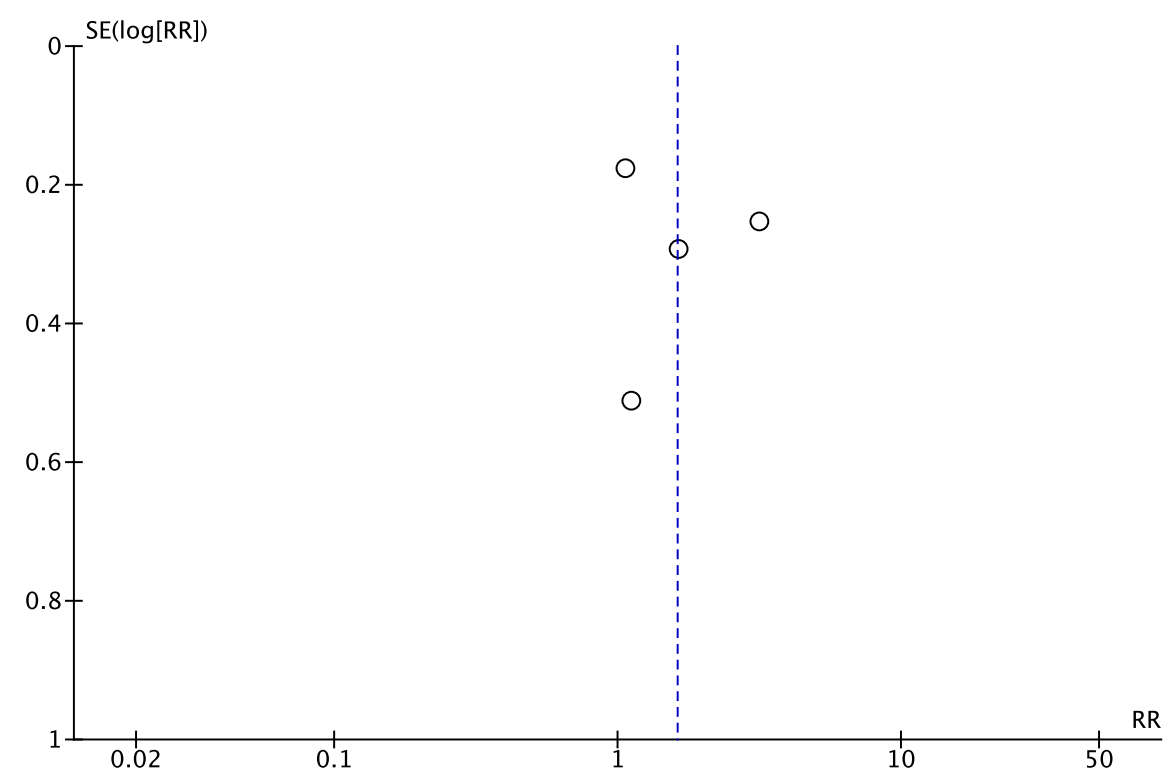

c

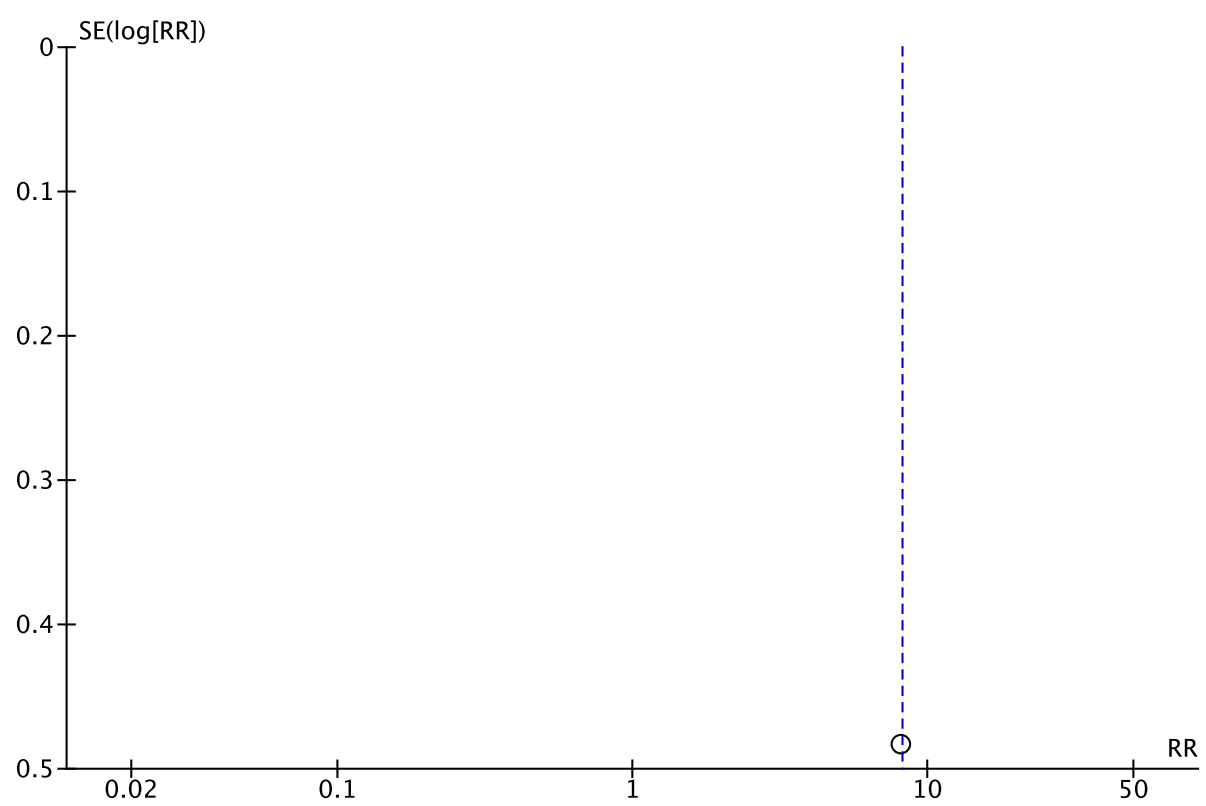

d

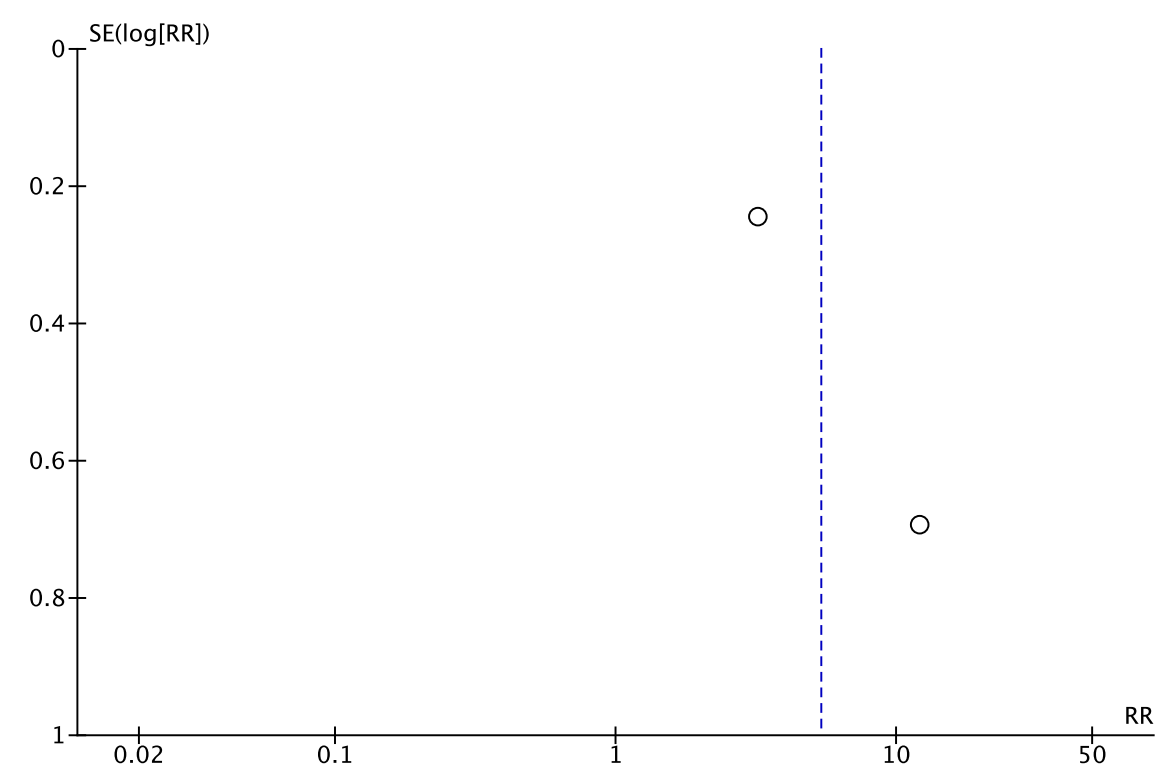

Figure S11. Funnel plots of studies investigating disease activity on withdrawing different DMARDs: **a**. bDMARDs; **b**. csDMARDs; **c**. tsDMARDs; **d**. all DMARDs

Figure S12

a

Study or Subgroup	DMARDs withdrawal Events	DMARDs withdrawal Total	Standard DMARDs Events	Standard DMARDs Total	Weight	Risk Ratio M-H, Random, 95% CI	Year
Ward 2025	22	69	4	33	6.8%	2.63 [0.99, 7.02]	2025
Kedra 2024	23	90	10	112	11.0%	2.86 [1.44, 5.70]	2024
Lillegraven 2023	21	43	0	41	1.1%	41.05 [2.57, 656.20]	2023
Curtis 2021	46	101	10	51	13.0%	2.32 [1.28, 4.21]	2021
Chatzidionysiou 2016	12	15	8	16	14.0%	1.60 [0.92, 2.78]	2016
Yamanaka 2016	10	28	3	32	5.1%	3.81 [1.16, 12.48]	2016
Moghadam 2016	213	531	34	286	20.3%	3.37 [2.42, 4.70]	2016
Fautrel 2016	26	64	18	73	15.4%	1.65 [1.00, 2.71]	2016
van Vollenhoven 2016	19	23	8	23	13.1%	2.38 [1.32, 4.29]	2016
**Total (95% CI)**		**964**		**667**	**100.0%**	**2.47 [1.84, 3.32]**	
Total events	392		95				

Heterogeneity: $Tau^2 = 0.08$; $Chi^2 = 14.78$, df = 8 (P = 0.06); $I^2 = 46\%$

Test for overall effect: Z = 5.98 (P < 0.00001)

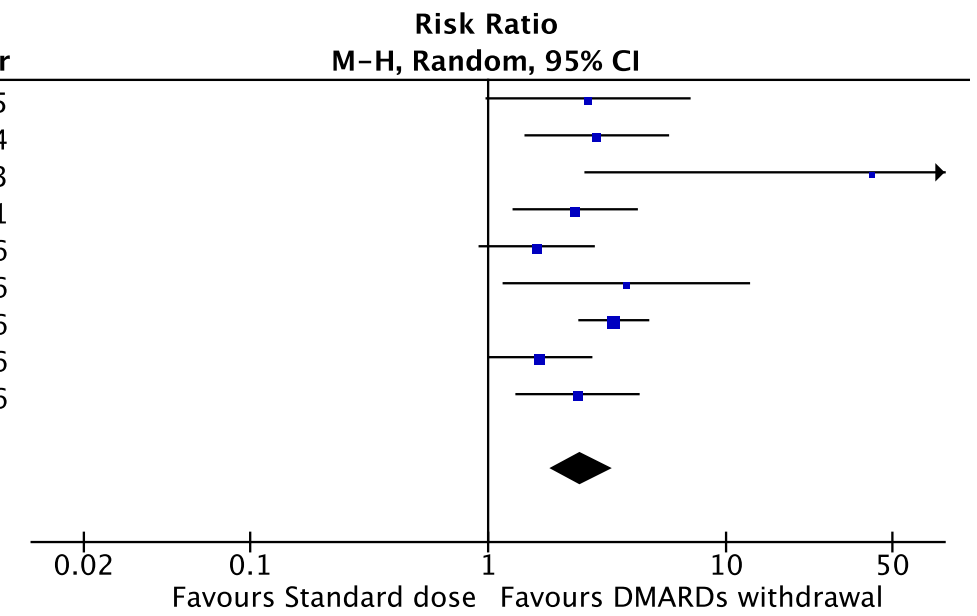

b

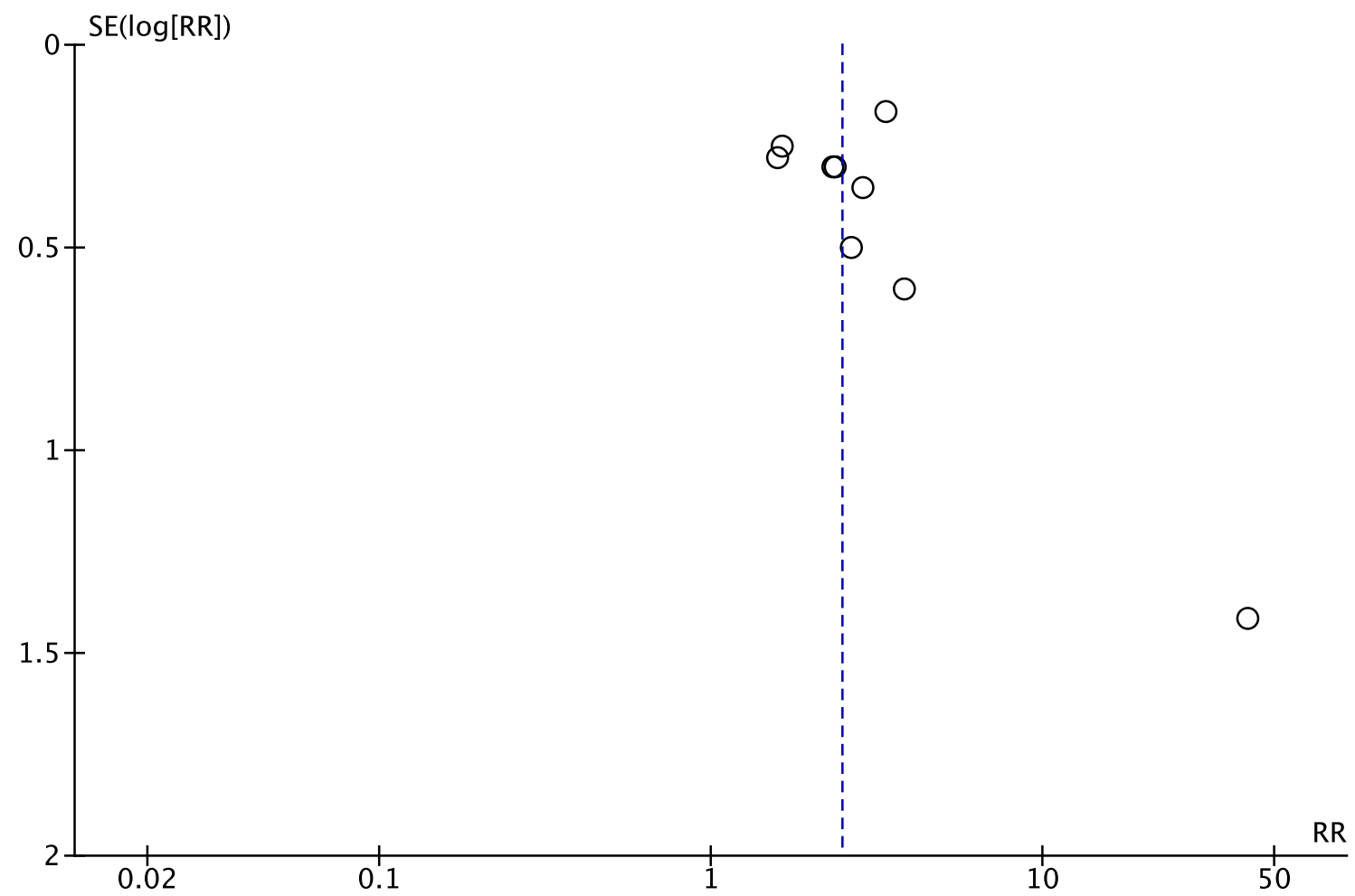

Figure S12. Forest plot (**a**) and funnel plot (**b**) on risk of disease flares following bDMARD withdrawal in the short term (0–9 months). **a**. bDMARD withdrawal significantly increased flare risk (RR 2.47, 95% CI 1.84–3.32; $p < 0.00001$) in the short term; **b**. Funnel plot indicated small-study effect.

Figure S13

a

Study or Subgroup	DMARDs withdrawal Events	Total	Standard DMARDs Events	Total	Weight	Risk Ratio M-H, Random, 95% CI	Year
Ward 2025	22	69	4	33	5.6%	2.63 [0.99, 7.02]	2025
Kedra 2024	23	90	10	112	10.1%	2.86 [1.44, 5.70]	2024
Lillegraven 2023	21	43	0	41	0.0%	41.05 [2.57, 656.20]	2023
Curtis 2021	46	101	10	51	12.5%	2.32 [1.28, 4.21]	2021
Chatzidionysiou 2016	12	15	8	16	14.0%	1.60 [0.92, 2.78]	2016
Yamanaka 2016	10	28	3	32	4.0%	3.81 [1.16, 12.48]	2016
Moghadam 2016	213	531	34	286	25.2%	3.37 [2.42, 4.70]	2016
Fautrel 2016	26	64	18	73	16.0%	1.65 [1.00, 2.71]	2016
van Vollenhoven 2016	19	23	8	23	12.7%	2.38 [1.32, 4.29]	2016
**Total (95% CI)**		**921**		**626**	**100.0%**	**2.41 [1.88, 3.09]**	
Total events	371		95				

Heterogeneity: $Tau^2 = 0.03$; $Chi^2 = 9.75$, df = 7 (P = 0.20); $I^2 = 28\%$
Test for overall effect: Z = 6.99 (P < 0.00001)

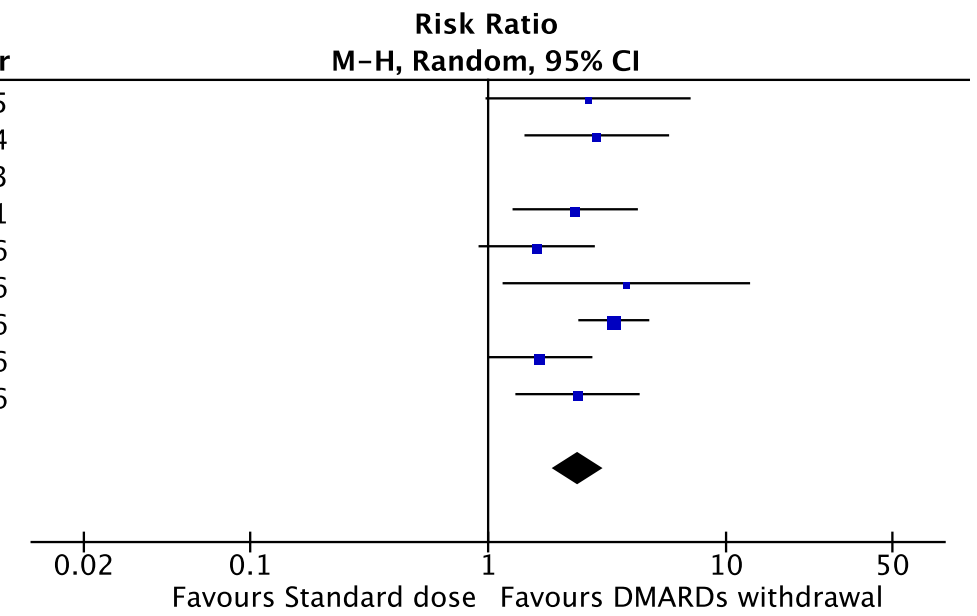

b

Study or Subgroup	DMARDs withdrawal Events	Total	Standard DMARDs Events	Total	Weight	Risk Ratio M-H, Fixed, 95% CI	Year
Ward 2025	22	69	4	33	5.0%	2.63 [0.99, 7.02]	2025
Kedra 2024	23	90	10	112	8.3%	2.86 [1.44, 5.70]	2024
Lillegraven 2023	21	43	0	41	0.5%	41.05 [2.57, 656.20]	2023
Curtis 2021	46	101	10	51	12.3%	2.32 [1.28, 4.21]	2021
Yamanaka 2016	10	28	3	32	2.6%	3.81 [1.16, 12.48]	2016
Moghadam 2016	213	531	34	286	41.0%	3.37 [2.42, 4.70]	2016
Fautrel 2016	26	64	18	73	15.6%	1.65 [1.00, 2.71]	2016
van Vollenhoven 2016	19	23	8	23	7.4%	2.38 [1.32, 4.29]	2016
Chatzidionysiou 2016	12	15	8	16	7.2%	1.60 [0.92, 2.78]	2016
**Total (95% CI)**		**964**		**667**	**100.0%**	**2.88 [2.35, 3.53]**	
Total events	392		95				

Heterogeneity: $Chi^2 = 14.78$, df = 8 (P = 0.06); $I^2 = 46\%$
Test for overall effect: Z = 10.23 (P < 0.00001)

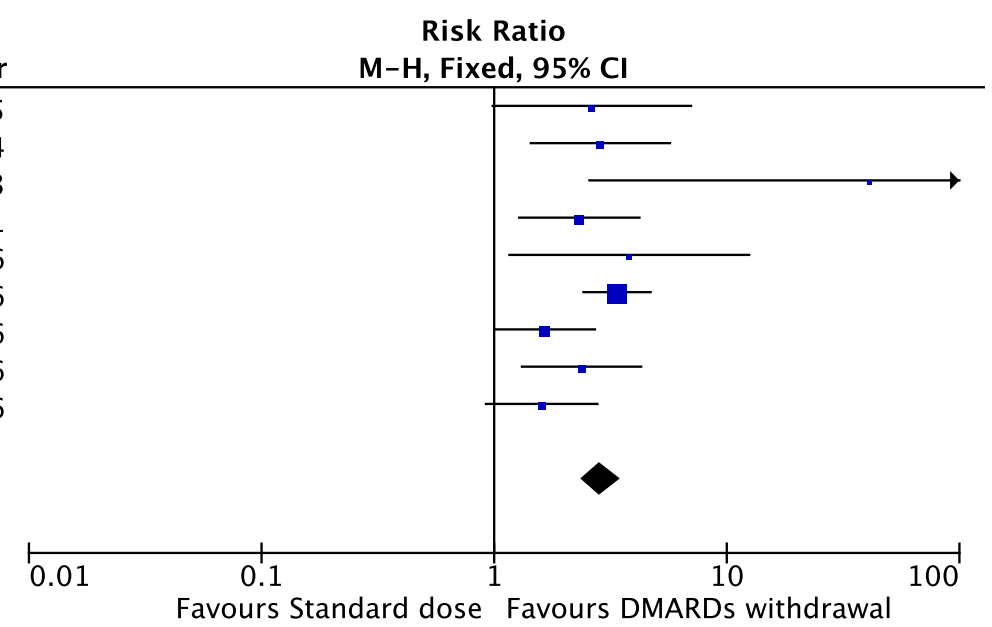

Figure S13. Sensitivity analyses confirmed the robustness of the finding that bDMARD withdrawal was associated with short-term increase in flare risk. **a**. leave-one-out (LOO) analysis excluding the ARCTIC REWIND study yielded a similar effect size (RR 2.41, 95% CI 1.88–3.09; $p < 0.00001$), but largely reduced the heterogeneity ($I^2 = 28\%$); **b**. fixed-effect model yielded consistent findings (RR 2.88, 95% CI 2.35–3.53; $p < 0.00001$).

Figure S14

a

Study or Subgroup	DMARDs withdrawal Events	DMARDs withdrawal Total	Standard DMARDs Events	Standard DMARDs Total	Weight	Risk Ratio M–H, Random, 95% CI	Year
Ward 2025	32	73	5	34	5.3%	2.98 [1.27, 6.98]	2025
Kedra 2024	44	90	18	112	8.5%	3.04 [1.90, 4.88]	2024
Lillegraven 2023	28	47	4	45	4.5%	6.70 [2.55, 17.58]	2023
Emery 2023	26	50	13	50	7.9%	2.00 [1.17, 3.43]	2023
Curtis 2021	72	101	24	51	10.1%	1.51 [1.10, 2.08]	2021
Weinblatt 2017	48	79	43	84	10.5%	1.19 [0.90, 1.56]	2017
Yamanaka 2016	13	28	4	32	4.3%	3.71 [1.37, 10.09]	2016
Moghadam 2016	272	531	52	286	10.6%	2.82 [2.17, 3.65]	2016
Fautrel 2016	49	64	34	73	10.4%	1.64 [1.24, 2.18]	2016
van Vollenhoven 2016	20	23	11	23	8.7%	1.82 [1.15, 2.87]	2016
van Herwaarden 2015	88	121	16	59	9.0%	2.68 [1.74, 4.13]	2015
Smolen 2013	113	197	35	201	10.0%	3.29 [2.38, 4.56]	2013
**Total (95% CI)**		**1404**		**1050**	**100.0%**	**2.30 [1.77, 2.99]**	
Total events	805		259				

Heterogeneity: Tau^2 = 0.15; Chi^2 = 52.49, df = 11 (P < 0.00001); I^2 = 79%

Test for overall effect: Z = 6.23 (P < 0.00001)

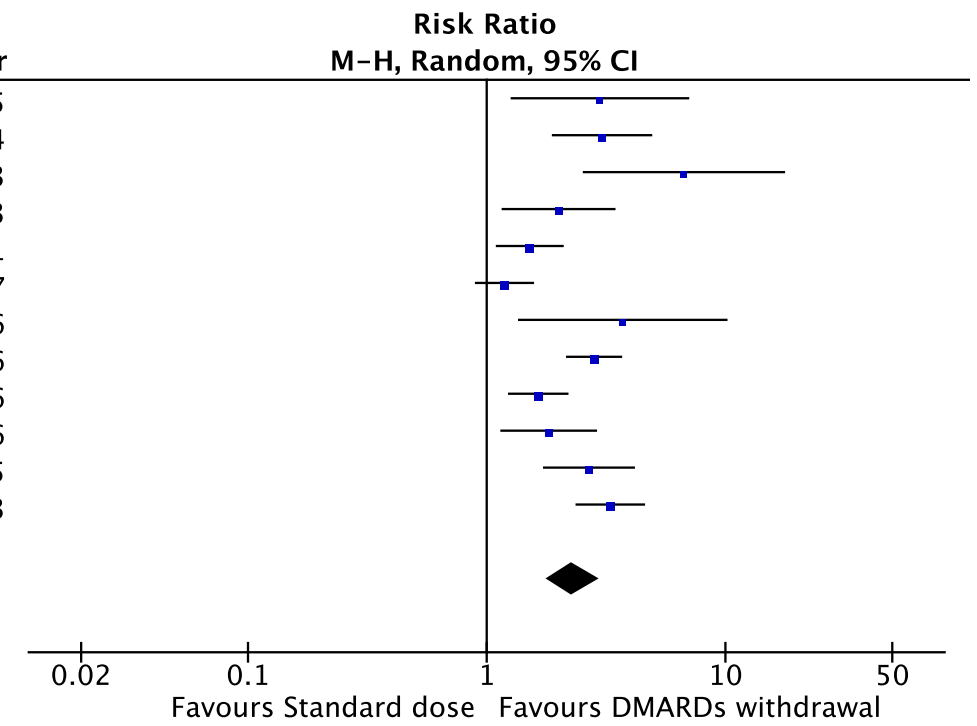

b

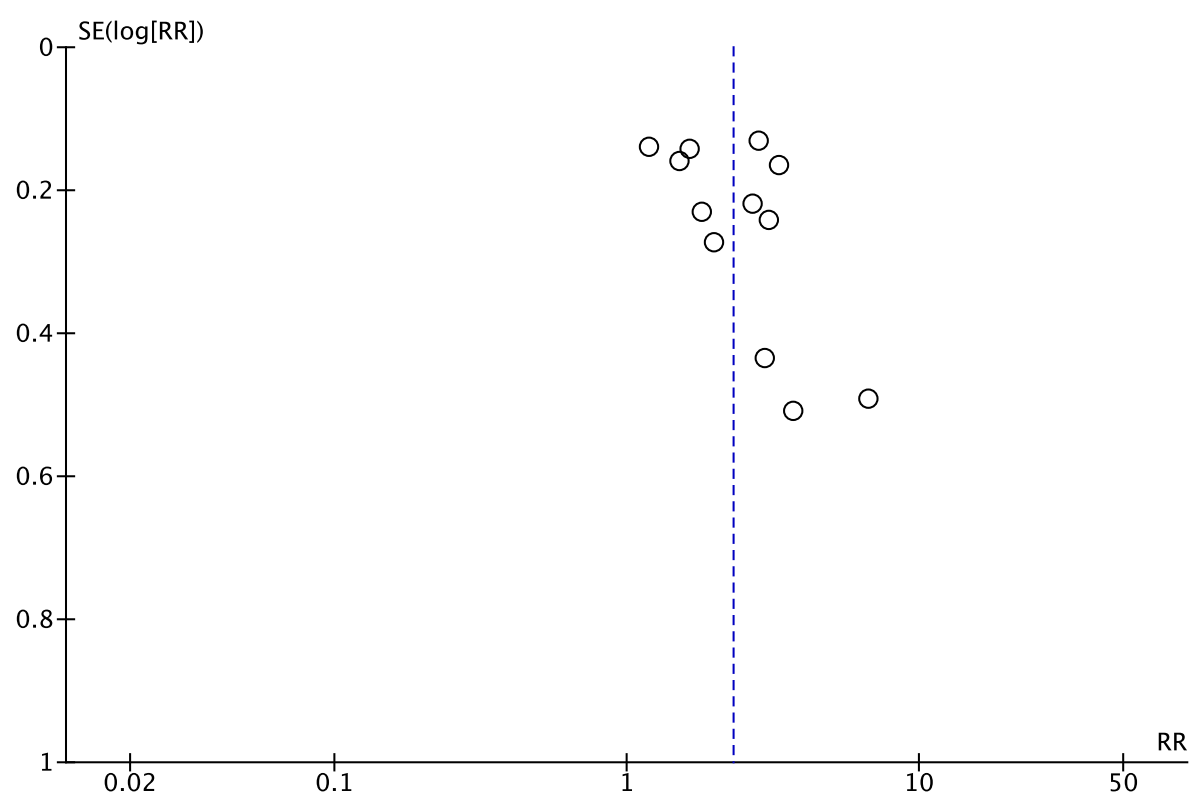

Figure S14. Forest plot (**a**) and funnel plot (**b**) on risk of disease flares following bDMARD withdrawal in the intermediate term (9–18 months). **a**. bDMARD withdrawal was associated with increased flare risk (RR 2.30, 95% CI 1.77–2.99; $p < 0.00001$) in the intermediate term, with high heterogeneity ($\tau^2 = 0.15$, $I^2 = 79\%$); **b**. Funnel plot indicated small-study effect.

Figure S15

a

Study or Subgroup	DMARDs withdrawal Events	DMARDs withdrawal Total	Standard DMARDs Events	Standard DMARDs Total	Weight	Odds Ratio M–H, Random, 95% CI	Year
Ward 2025	32	73	5	34	5.7%	4.53 [1.58, 13.01]	2025
Kedra 2024	44	90	18	112	9.8%	5.00 [2.60, 9.59]	2024
Lillegraven 2023	28	47	4	45	4.9%	15.11 [4.64, 49.18]	2023
Emery 2023	26	50	13	50	7.6%	3.08 [1.33, 7.15]	2023
Curtis 2021	72	101	24	51	9.2%	2.79 [1.39, 5.62]	2021
Weinblatt 2017	48	79	43	84	10.2%	1.48 [0.79, 2.75]	2017
Yamanaka 2016	13	28	4	32	4.3%	6.07 [1.68, 21.91]	2016
Moghadam 2016	272	531	52	286	14.3%	4.73 [3.35, 6.68]	2016
Fautrel 2016	49	64	34	73	8.7%	3.75 [1.79, 7.84]	2016
van Vollenhoven 2016	20	23	11	23	3.5%	7.27 [1.68, 31.43]	2016
van Herwaarden 2015	88	121	16	59	9.2%	7.17 [3.56, 14.43]	2015
Smolen 2013	113	197	35	201	12.6%	6.38 [4.02, 10.12]	2013
**Total (95% CI)**		**1404**		**1050**	**100.0%**	**4.46 [3.28, 6.07]**	
Total events	805		259				

Heterogeneity: Tau² = 0.14; Chi² = 23.87, df = 11 (P = 0.01); I² = 54%
Test for overall effect: Z = 9.52 (P < 0.00001)

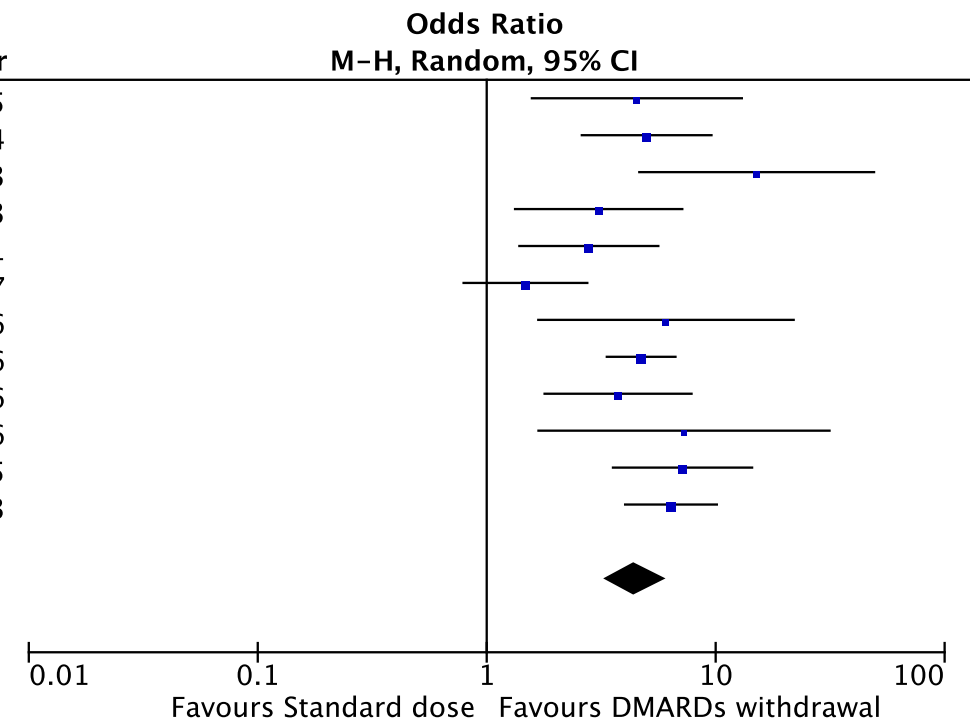

b

Study or Subgroup	DMARDs withdrawal Events	DMARDs withdrawal Total	Standard DMARDs Events	Standard DMARDs Total	Weight	Risk Ratio M–H, Random, 95% CI	Year
Ward 2025	32	73	5	34	5.5%	2.98 [1.27, 6.98]	2025
Kedra 2024	44	90	18	112	8.9%	3.04 [1.90, 4.88]	2024
Lillegraven 2023	28	47	4	45	4.8%	6.70 [2.55, 17.58]	2023
Emery 2023	26	50	13	50	8.3%	2.00 [1.17, 3.43]	2023
Curtis 2021	72	101	24	51	10.6%	1.51 [1.10, 2.08]	2021
Weinblatt 2017	48	79	43	84	11.0%	1.19 [0.90, 1.56]	2017
Yamanaka 2016	13	28	4	32	0.0%	3.71 [1.37, 10.09]	2016
Moghadam 2016	272	531	52	286	11.1%	2.82 [2.17, 3.65]	2016
Fautrel 2016	49	64	34	73	10.9%	1.64 [1.24, 2.18]	2016
van Vollenhoven 2016	20	23	11	23	9.1%	1.82 [1.15, 2.87]	2016
van Herwaarden 2015	88	121	16	59	9.4%	2.68 [1.74, 4.13]	2015
Smolen 2013	113	197	35	201	10.5%	3.29 [2.38, 4.56]	2013
**Total (95% CI)**		**1376**		**1018**	**100.0%**	**2.25 [1.72, 2.94]**	
Total events	792		255				

Heterogeneity: Tau² = 0.15; Chi² = 51.07, df = 10 (P < 0.00001); I² = 80%
Test for overall effect: Z = 5.93 (P < 0.00001)

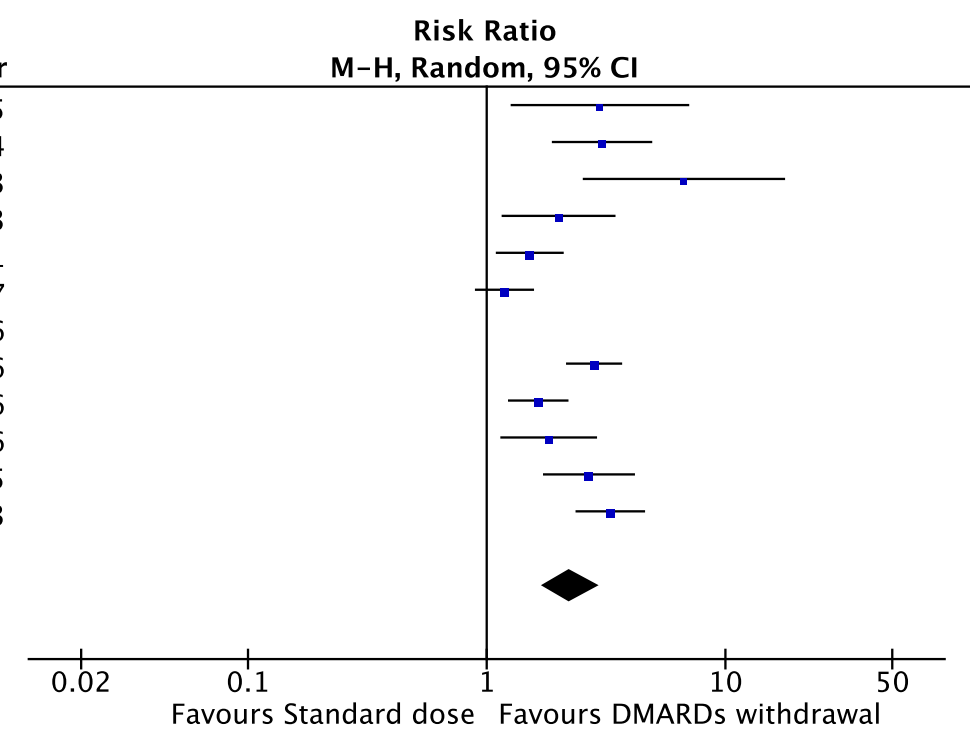

Figure S15. Sensitivity analyses confirmed the robustness of the finding that bDMARD withdrawal was associated with intermediate-term increase in flare risk. **a**. analysis using Odd Ratio (OR) as effect size yielded a pooled OR of 4.46 (95% CI 3.28–6.07; $p < 0.00001$); **b**. restricting analyses by excluding high risk-of-bias (RoB) studies produced similar results (RR 2.25, 95% CI 1.72–2.94).

Figure S16

Study or Subgroup	DMARDs withdrawal Events	DMARDs withdrawal Total	Standard DMARDs Events	Standard DMARDs Total	Weight	Risk Ratio M-H, Random, 95% CI	Year
**2.18.1 Sustained remission**							
Ward 2025	32	73	5	34	5.3%	2.98 [1.27, 6.98]	2025
Kedra 2024	44	90	18	112	8.5%	3.04 [1.90, 4.88]	2024
Lillegraven 2023	28	47	4	45	4.5%	6.70 [2.55, 17.58]	2023
Emery 2023	26	50	13	50	7.9%	2.00 [1.17, 3.43]	2023
Curtis 2021	72	101	24	51	10.1%	1.51 [1.10, 2.08]	2021
Yamanaka 2016	13	28	4	32	4.3%	3.71 [1.37, 10.09]	2016
Fautrel 2016	49	64	34	73	10.4%	1.64 [1.24, 2.18]	2016
**Subtotal (95% CI)**		**453**		**397**	**51.2%**	**2.35 [1.67, 3.32]**	
Total events	264		102				
Heterogeneity: $Tau^2 = 0.13$; $Chi^2 = 18.19$, df = 6 (P = 0.006); $I^2 = 67\%$							
Test for overall effect: Z = 4.87 (P < 0.00001)							
**2.18.2 Sustained LDA**							
Weinblatt 2017	48	79	43	84	10.5%	1.19 [0.90, 1.56]	2017
Moghadam 2016	272	531	52	286	10.6%	2.82 [2.17, 3.65]	2016
van Vollenhoven 2016	20	23	11	23	8.7%	1.82 [1.15, 2.87]	2016
van Herwaarden 2015	88	121	16	59	9.0%	2.68 [1.74, 4.13]	2015
Smolen 2013	113	197	35	201	10.0%	3.29 [2.38, 4.56]	2013
**Subtotal (95% CI)**		**951**		**653**	**48.8%**	**2.22 [1.44, 3.40]**	
Total events	541		157				
Heterogeneity: $Tau^2 = 0.21$; $Chi^2 = 32.98$, df = 4 (P < 0.00001); $I^2 = 88\%$							
Test for overall effect: Z = 3.64 (P = 0.0003)							
**Total (95% CI)**		**1404**		**1050**	**100.0%**	**2.30 [1.77, 2.99]**	
Total events	805		259				
Heterogeneity: $Tau^2 = 0.15$; $Chi^2 = 52.49$, df = 11 (P < 0.00001); $I^2 = 79\%$							
Test for overall effect: Z = 6.23 (P < 0.00001)							
Test for subgroup differences: $Chi^2 = 0.05$, df = 1 (P = 0.83), $I^2 = 0\%$							

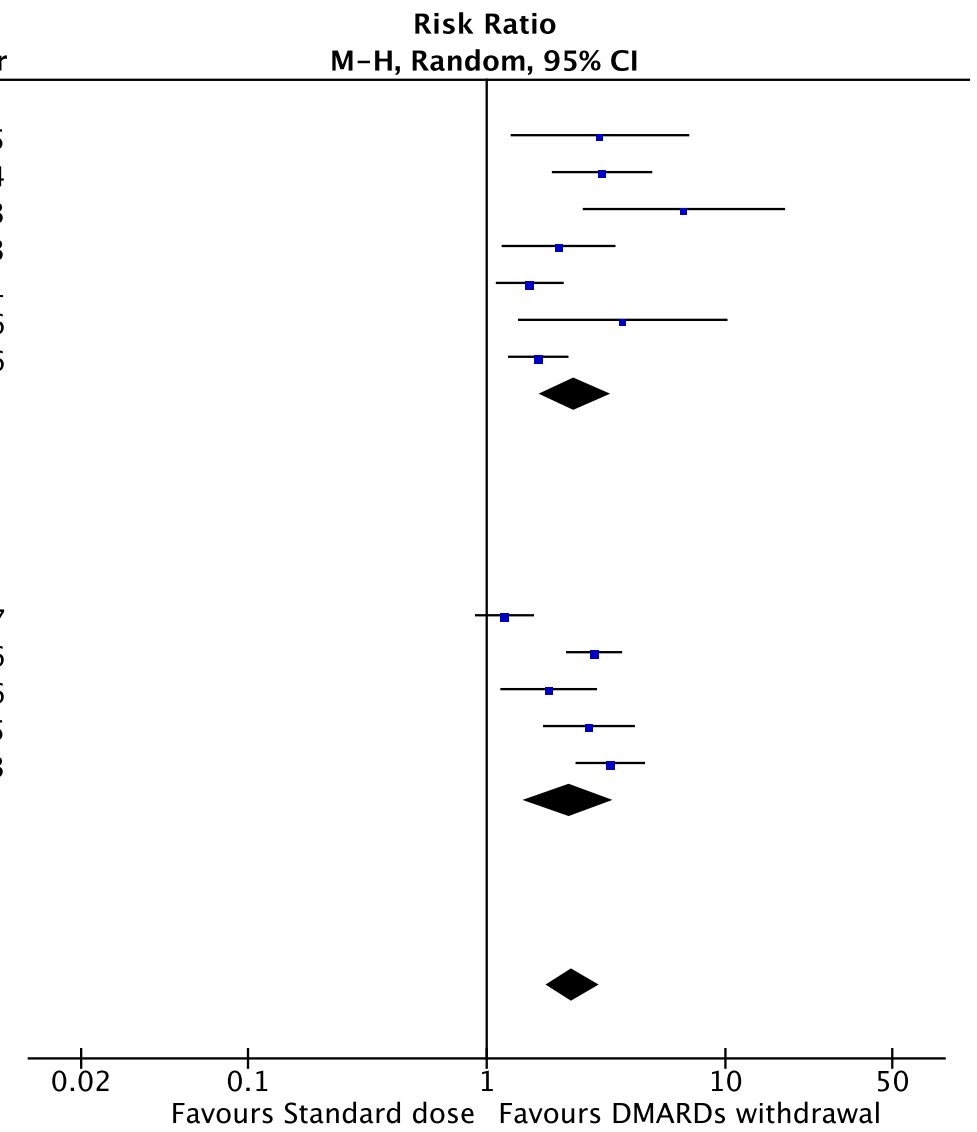

Figure S16. Subgroup analysis on disease flares upon bDMARD withdrawal during the intermediate-term follow-up period (remission *vs* LDA).

Figure S17

Study or Subgroup	DMARDs withdrawal Events	DMARDs withdrawal Total	Standard DMARDs Events	Standard DMARDs Total	Weight	Risk Ratio M-H, Random, 95% CI	Year
**2.19.1 Immediate withdrawal**							
Ward 2025	32	73	5	34	5.3%	2.98 [1.27, 6.98]	2025
Curtis 2021	72	101	24	51	10.1%	1.51 [1.10, 2.08]	2021
Weinblatt 2017	48	79	43	84	10.5%	1.19 [0.90, 1.56]	2017
Yamanaka 2016	13	28	4	32	4.3%	3.71 [1.37, 10.09]	2016
Moghadam 2016	272	531	52	286	10.6%	2.82 [2.17, 3.65]	2016
van Vollenhoven 2016	20	23	11	23	8.7%	1.82 [1.15, 2.87]	2016
Smolen 2013	113	197	35	201	10.0%	3.29 [2.38, 4.56]	2013
**Subtotal (95% CI)**		**1032**		**711**	**59.6%**	**2.17 [1.49, 3.16]**	
Total events	570		174				
Heterogeneity: $Tau^2 = 0.20$; $Chi^2 = 38.74$, df = 6 (P < 0.00001); $I^2 = 85\%$							
Test for overall effect: Z = 4.03 (P < 0.0001)							
**2.19.2 Tapering to withdrawal**							
Kedra 2024	44	90	18	112	8.5%	3.04 [1.90, 4.88]	2024
Emery 2023	26	50	13	50	7.9%	2.00 [1.17, 3.43]	2023
Lillegraven 2023	28	47	4	45	4.5%	6.70 [2.55, 17.58]	2023
Fautrel 2016	49	64	34	73	10.4%	1.64 [1.24, 2.18]	2016
van Herwaarden 2015	88	121	16	59	9.0%	2.68 [1.74, 4.13]	2015
**Subtotal (95% CI)**		**372**		**339**	**40.4%**	**2.52 [1.70, 3.74]**	
Total events	235		85				
Heterogeneity: $Tau^2 = 0.13$; $Chi^2 = 13.52$, df = 4 (P = 0.009); $I^2 = 70\%$							
Test for overall effect: Z = 4.62 (P < 0.00001)							
**Total (95% CI)**		**1404**		**1050**	**100.0%**	**2.30 [1.77, 2.99]**	
Total events	805		259				
Heterogeneity: $Tau^2 = 0.15$; $Chi^2 = 52.49$, df = 11 (P < 0.00001); $I^2 = 79\%$							
Test for overall effect: Z = 6.23 (P < 0.00001)							
Test for subgroup differences: $Chi^2 = 0.30$, df = 1 (P = 0.58), $I^2 = 0\%$							

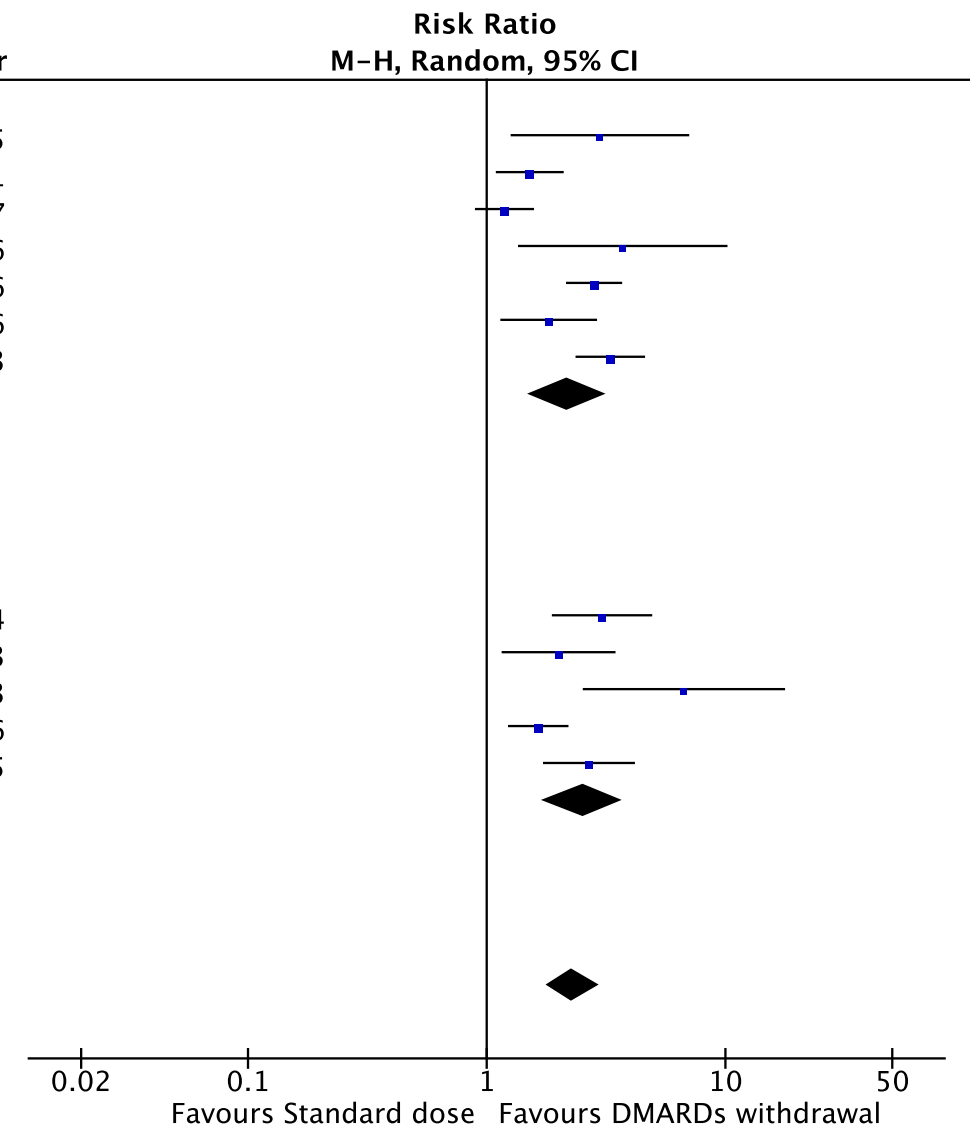

Figure S17. Subgroup analysis on disease flares upon bDMARD withdrawal during the intermediate-term follow-up period (immediate withdrawal *vs* tapering to withdrawal)

Figure S18

Study or Subgroup	DMARDs withdrawal Events	DMARDs withdrawal Total	Standard DMARDs Events	Standard DMARDs Total	Weight	Risk Ratio M-H, Random, 95% CI	Year
**2.20.1 With csDMARDs**							
Ward 2025	32	73	5	34	5.3%	2.98 [1.27, 6.98]	2025
Emery 2023	26	50	13	50	7.9%	2.00 [1.17, 3.43]	2023
Lillegraven 2023	28	47	4	45	4.5%	6.70 [2.55, 17.58]	2023
Curtis 2021	72	101	24	51	10.1%	1.51 [1.10, 2.08]	2021
Weinblatt 2017	48	79	43	84	10.5%	1.19 [0.90, 1.56]	2017
Yamanaka 2016	13	28	4	32	4.3%	3.71 [1.37, 10.09]	2016
Moghadam 2016	272	531	52	286	10.6%	2.82 [2.17, 3.65]	2016
van Vollenhoven 2016	20	23	11	23	8.7%	1.82 [1.15, 2.87]	2016
Smolen 2013	113	197	35	201	10.0%	3.29 [2.38, 4.56]	2013
**Subtotal (95% CI)**		**1129**		**806**	**72.1%**	**2.32 [1.65, 3.27]**	
Total events	624		191				
Heterogeneity: $Tau^2 = 0.20$; $Chi^2 = 45.25$, df = 8 (P < 0.00001); $I^2 = 82\%$							
Test for overall effect: Z = 4.83 (P < 0.00001)							
**2.20.2 Without csDMARDs**							
Fautrel 2016	49	64	34	73	10.4%	1.64 [1.24, 2.18]	2016
van Herwaarden 2015	88	121	16	59	9.0%	2.68 [1.74, 4.13]	2015
**Subtotal (95% CI)**		**185**		**132**	**19.4%**	**2.05 [1.23, 3.40]**	
Total events	137		50				
Heterogeneity: $Tau^2 = 0.10$; $Chi^2 = 3.93$, df = 1 (P = 0.05); $I^2 = 75\%$							
Test for overall effect: Z = 2.76 (P = 0.006)							
**2.20.3 Uncertain**							
Kedra 2024	44	90	18	112	8.5%	3.04 [1.90, 4.88]	2024
**Subtotal (95% CI)**		**90**		**112**	**8.5%**	**3.04 [1.90, 4.88]**	
Total events	44		18				
Heterogeneity: Not applicable							
Test for overall effect: Z = 4.61 (P < 0.00001)							
**Total (95% CI)**		**1404**		**1050**	**100.0%**	**2.30 [1.77, 2.99]**	
Total events	805		259				
Heterogeneity: $Tau^2 = 0.15$; $Chi^2 = 52.49$, df = 11 (P < 0.00001); $I^2 = 79\%$							
Test for overall effect: Z = 6.23 (P < 0.00001)							
Test for subgroup differences: $Chi^2 = 1.37$, df = 2 (P = 0.50), $I^2 = 0\%$							

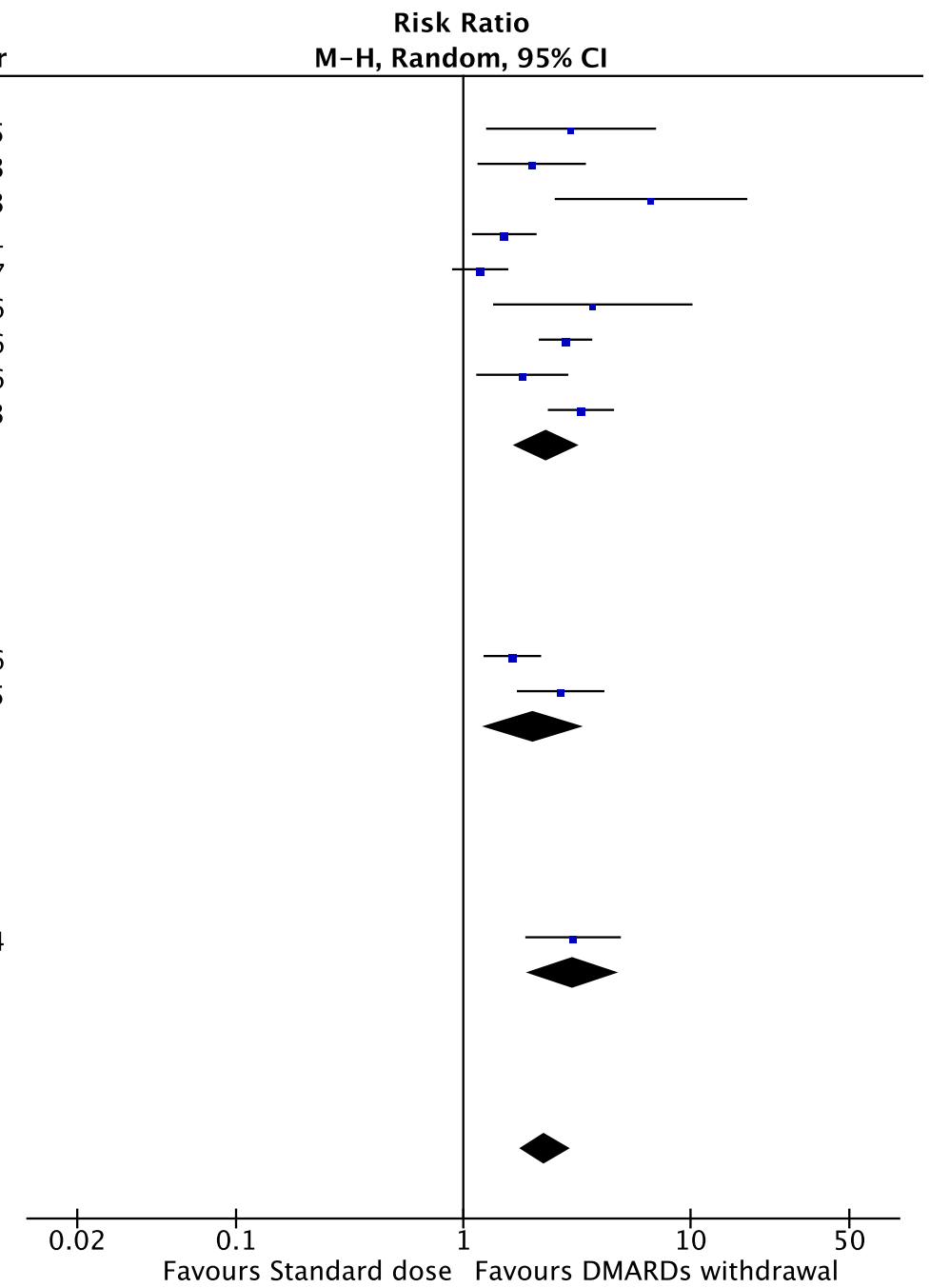

Figure S18. Subgroup analysis on disease flares upon bDMARD withdrawal during the intermediate-term follow-up period (with csDMARDs *vs* without csDMARDs)

Figure S19

a

Study or Subgroup	DMARDs withdrawal Events	DMARDs withdrawal Total	Standard DMARDs Events	Standard DMARDs Total	Weight	Risk Ratio M-H, Random, 95% CI	Year
**2.21.1 Flare**							
Ward 2025	32	73	5	34	5.3%	2.98 [1.27, 6.98]	2025
Kedra 2024	44	90	18	112	8.5%	3.04 [1.90, 4.88]	2024
Lillegraven 2023	28	47	4	45	4.5%	6.70 [2.55, 17.58]	2023
Weinblatt 2017	48	79	43	84	10.5%	1.19 [0.90, 1.56]	2017
Yamanaka 2016	13	28	4	32	4.3%	3.71 [1.37, 10.09]	2016
Moghadam 2016	272	531	52	286	10.6%	2.82 [2.17, 3.65]	2016
van Vollenhoven 2016	20	23	11	23	8.7%	1.82 [1.15, 2.87]	2016
van Herwaarden 2015	88	121	16	59	9.0%	2.68 [1.74, 4.13]	2015
**Subtotal (95% CI)**		**992**		**675**	**61.5%**	**2.55 [1.75, 3.72]**	
Total events	545		153				
Heterogeneity: $Tau^2 = 0.21$; $Chi^2 = 36.28$, df = 7 (P < 0.00001); $I^2 = 81\%$							
Test for overall effect: Z = 4.85 (P < 0.00001)							
**2.21.2 Other outcomes**							
Emery 2023	26	50	13	50	7.9%	2.00 [1.17, 3.43]	2023
Curtis 2021	72	101	24	51	10.1%	1.51 [1.10, 2.08]	2021
Fautrel 2016	49	64	34	73	10.4%	1.64 [1.24, 2.18]	2016
Smolen 2013	113	197	35	201	10.0%	3.29 [2.38, 4.56]	2013
**Subtotal (95% CI)**		**412**		**375**	**38.5%**	**2.01 [1.36, 2.96]**	
Total events	260		106				
Heterogeneity: $Tau^2 = 0.12$; $Chi^2 = 15.08$, df = 3 (P = 0.002); $I^2 = 80\%$							
Test for overall effect: Z = 3.50 (P = 0.0005)							
**Total (95% CI)**		**1404**		**1050**	**100.0%**	**2.30 [1.77, 2.99]**	
Total events	805		259				
Heterogeneity: $Tau^2 = 0.15$; $Chi^2 = 52.49$, df = 11 (P < 0.00001); $I^2 = 79\%$							
Test for overall effect: Z = 6.23 (P < 0.00001)							
Test for subgroup differences: $Chi^2 = 0.75$, df = 1 (P = 0.39), $I^2 = 0\%$							

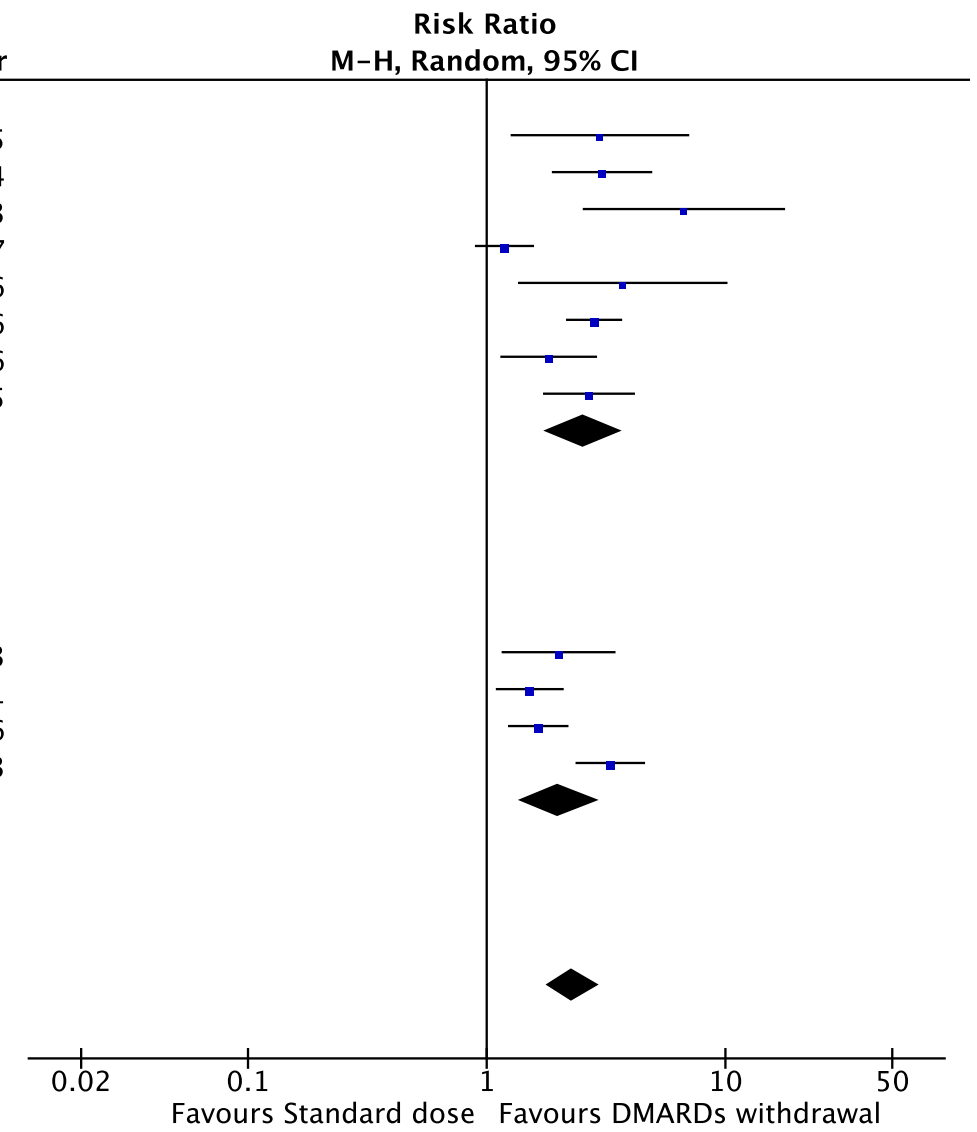

b

Study or Subgroup	DMARDs withdrawal Events	DMARDs withdrawal Total	Standard DMARDs Events	Standard DMARDs Total	Weight	Risk Ratio M-H, Random, 95% CI	Year
**2.21.1 Flare**							
Ward 2025	32	73	5	34	5.6%	2.98 [1.27, 6.98]	2025
Kedra 2024	44	90	18	112	10.6%	3.04 [1.90, 4.88]	2024
Lillegraven 2023	28	47	4	45	4.7%	6.70 [2.55, 17.58]	2023
Weinblatt 2017	48	79	43	84	0.0%	1.19 [0.90, 1.56]	2017
Yamanaka 2016	13	28	4	32	4.5%	3.71 [1.37, 10.09]	2016
Moghadam 2016	272	531	52	286	14.7%	2.82 [2.17, 3.65]	2016
van Vollenhoven 2016	20	23	11	23	11.0%	1.82 [1.15, 2.87]	2016
van Herwaarden 2015	88	121	16	59	11.4%	2.68 [1.74, 4.13]	2015
**Subtotal (95% CI)**		**913**		**591**	**62.5%**	**2.77 [2.25, 3.42]**	
Total events	497		110				
Heterogeneity: $Tau^2 = 0.01$; $Chi^2 = 7.36$, df = 6 (P = 0.29); $I^2 = 19\%$							
Test for overall effect: Z = 9.53 (P < 0.00001)							
**2.21.2 Other outcomes**							
Emery 2023	26	50	13	50	9.5%	2.00 [1.17, 3.43]	2023
Curtis 2021	72	101	24	51	13.6%	1.51 [1.10, 2.08]	2021
Fautrel 2016	49	64	34	73	14.3%	1.64 [1.24, 2.18]	2016
Smolen 2013	113	197	35	201	0.0%	3.29 [2.38, 4.56]	2013
**Subtotal (95% CI)**		**215**		**174**	**37.5%**	**1.64 [1.34, 1.99]**	
Total events	147		71				
Heterogeneity: $Tau^2 = 0.00$; $Chi^2 = 0.77$, df = 2 (P = 0.68); $I^2 = 0\%$							
Test for overall effect: Z = 4.93 (P < 0.00001)							
**Total (95% CI)**		**1128**		**765**	**100.0%**	**2.34 [1.84, 2.99]**	
Total events	644		181				
Heterogeneity: $Tau^2 = 0.09$; $Chi^2 = 25.66$, df = 9 (P = 0.002); $I^2 = 65\%$							
Test for overall effect: Z = 6.84 (P < 0.00001)							
Test for subgroup differences: $Chi^2 = 13.01$, df = 1 (P = 0.0003), $I^2 = 92.3\%$							

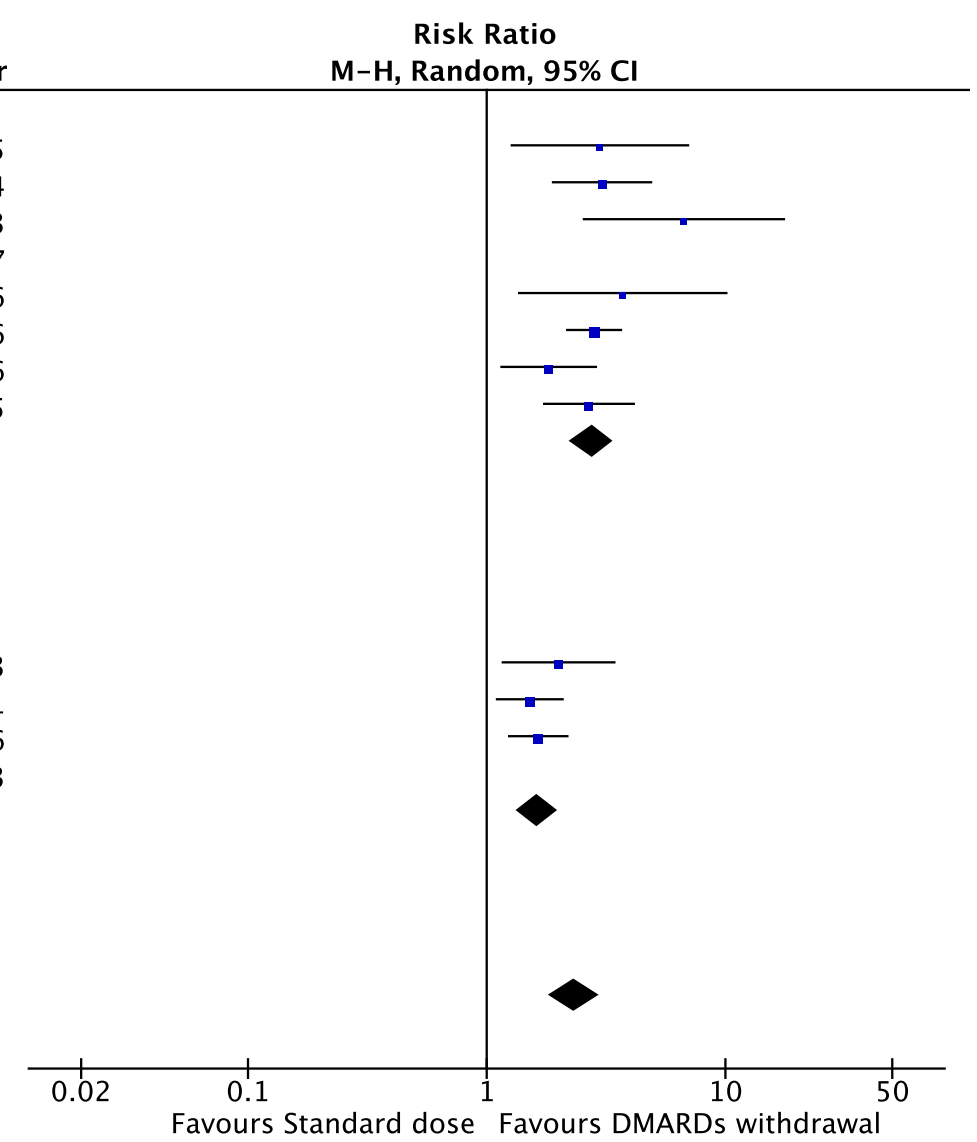

Figure S19. Subgroup analysis (**a**) followed by leave-one-out (LOO) sensitivity analysis (**b**) on disease flares upon bDMARD withdrawal during the intermediate-term follow-up period (flare defined *vs* flare undefined). **a**. risk ratio (RRs) of the subgroups were similar to the pooled effect size; **b**. LOO analysis identified one study in each subgroup significantly contributing to heterogeneity.

Figure S20

a

Study or Subgroup	DMARDs withdrawal Events	DMARDs withdrawal Total	Standard DMARDs Events	Standard DMARDs Total	Weight	Risk Ratio M–H, Random, 95% CI	Year
Kjorholt 2025	35	47	7	45	19.9%	4.79 [2.37, 9.65]	2025
Kedra 2024	76	113	33	115	39.7%	2.34 [1.71, 3.21]	2024
Bouman 2017	96	115	25	57	40.4%	1.90 [1.40, 2.58]	2017
**Total (95% CI)**		**275**		**217**	**100.0%**	**2.48 [1.67, 3.69]**	
Total events	207		65				

Heterogeneity: $Tau^2 = 0.08$; $Chi^2 = 5.90$, df = 2 (P = 0.05); $I^2 = 66\%$
Test for overall effect: Z = 4.50 (P < 0.00001)

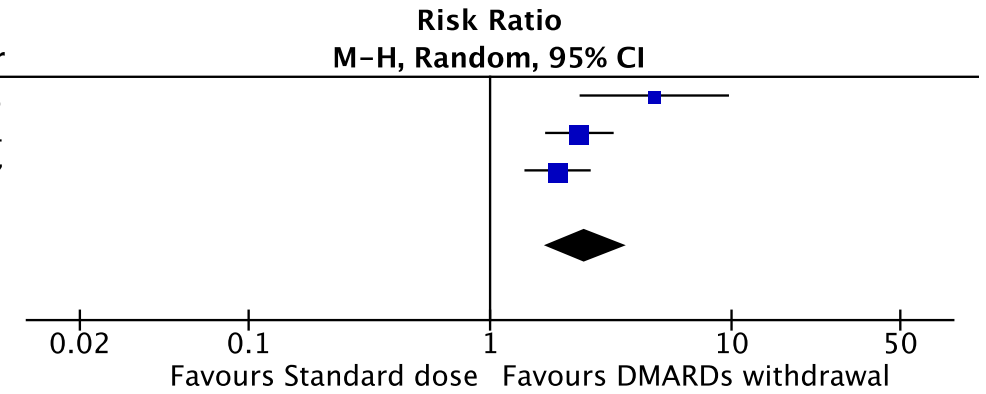

b

Study or Subgroup	DMARDs withdrawal Events	DMARDs withdrawal Total	Standard DMARDs Events	Standard DMARDs Total	Weight	Risk Ratio M–H, Random, 95% CI	Year
Kjorholt 2025	35	47	7	45	0.0%	4.79 [2.37, 9.65]	2025
Kedra 2024	76	113	33	115	48.3%	2.34 [1.71, 3.21]	2024
Bouman 2017	96	115	25	57	51.7%	1.90 [1.40, 2.58]	2017
**Total (95% CI)**		**228**		**172**	**100.0%**	**2.10 [1.69, 2.62]**	
Total events	172		58				

Heterogeneity: $Tau^2 = 0.00$; $Chi^2 = 0.87$, df = 1 (P = 0.35); $I^2 = 0\%$
Test for overall effect: Z = 6.65 (P < 0.00001)

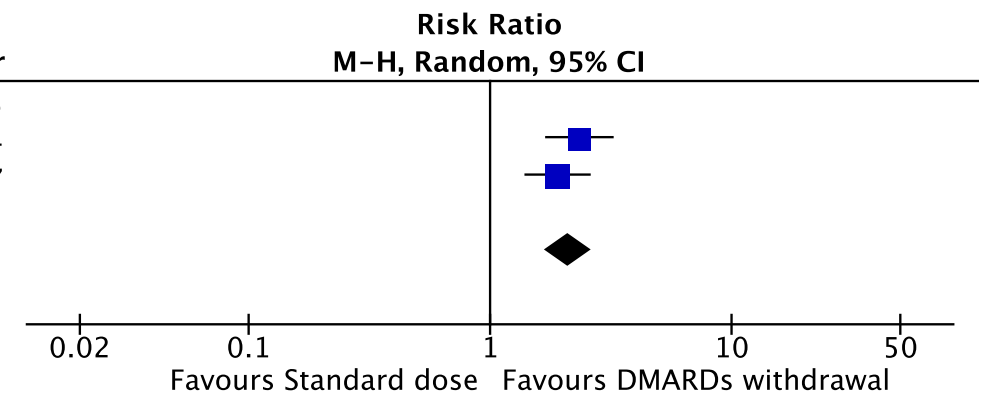

Figure S20. Forest plot (**a**) and leave-one-out (LOO) sensitivity analysis (**b**) on risk of disease flare following bDMARD withdrawal in the long term (>18 months). **a**. bDMARD withdrawal was associated with increased flare risk (RR 2.48, 95% CI 1.67–3.69; $p < 0.00001$), with substantial heterogeneity ($\tau^2 = 0.08$; $I^2 = 66\%$); **b**. LOO analysis identified the ARCTIC REWIND study as the major contributor to heterogeneity.

Figure S21

a

Study or Subgroup	DMARDs withdrawal Events	DMARDs withdrawal Total	Standard DMARDs Events	Standard DMARDs Total	Weight	Risk Ratio M–H, Random, 95% CI	Year
Asai 2025	7	43	6	41	17.0%	1.11 [0.41, 3.03]	2025
Emery 2023	17	47	11	50	41.1%	1.64 [0.86, 3.14]	2023
Curtis 2021	28	101	10	51	41.9%	1.41 [0.75, 2.68]	2021
**Total (95% CI)**		**191**		**142**	**100.0%**	**1.44 [0.95, 2.18]**	
Total events	52		27				

Heterogeneity: Tau² = 0.00; Chi² = 0.42, df = 2 (P = 0.81); I² = 0%
Test for overall effect: Z = 1.74 (P = 0.08)

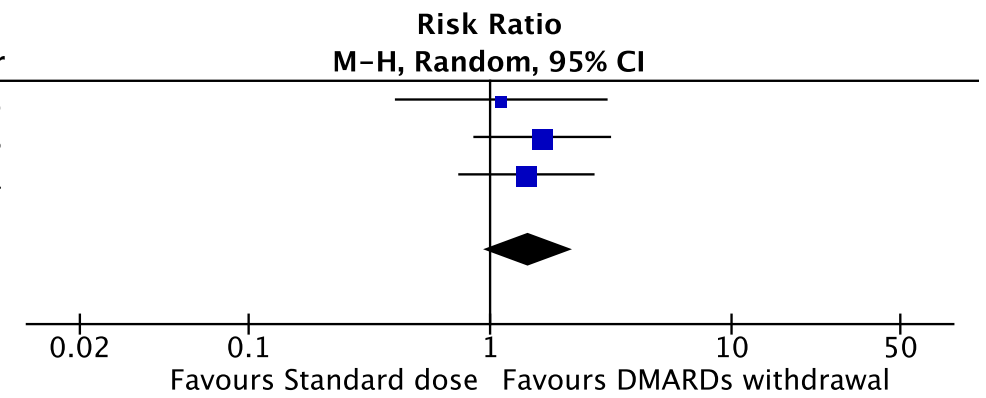

b

Study or Subgroup	DMARDs withdrawal Events	DMARDs withdrawal Total	Standard DMARDs Events	Standard DMARDs Total	Weight	Risk Ratio M–H, Random, 95% CI	Year
Emery 2023	20	47	13	50	35.1%	1.64 [0.92, 2.90]	2023
Curtis 2021	51	101	24	51	64.9%	1.07 [0.76, 1.52]	2021
**Total (95% CI)**		**148**		**101**	**100.0%**	**1.24 [0.84, 1.85]**	
Total events	71		37				

Heterogeneity: Tau² = 0.03; Chi² = 1.54, df = 1 (P = 0.21); I² = 35%
Test for overall effect: Z = 1.08 (P = 0.28)

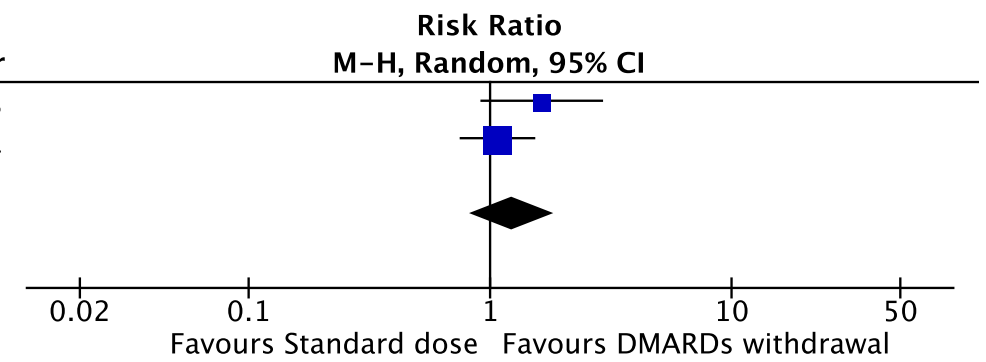

c

Study or Subgroup	DMARDs withdrawal Events	DMARDs withdrawal Total	Standard DMARDs Events	Standard DMARDs Total	Weight	Risk Ratio M–H, Random, 95% CI	Year
Kjorholt 2024	24	37	16	78	100.0%	3.16 [1.92, 5.20]	2024
**Total (95% CI)**		**37**		**78**	**100.0%**	**3.16 [1.92, 5.20]**	
Total events	24		16				

Heterogeneity: Not applicable
Test for overall effect: Z = 4.54 (P < 0.00001)

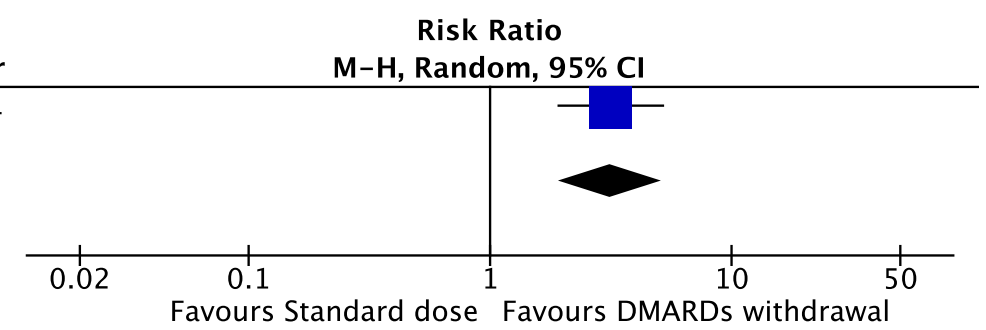

Figure S21. csDMARD withdrawal was not associated with a significant increase in flare risk in the short term (RR 1.44, 95% CI 0.95–2.18; $p = 0.08$) (**a**) or the intermediate term (RR 1.24, 95% CI 0.84–1.85; $p = 0.28$) (**b**), and only one study (ARCTIC REWIND) reported the long-term effect size (RR 3.16, 95% CI 1.92–5.20; $p < 0.00001$) (**c**).

Figure S22

Study or Subgroup	DMARDs withdrawal Events	Total	Standard DMARDs Events	Total	Weight	Risk Ratio M-H, Random, 95% CI	Year
Wang 2023	31	39	4	41	100.0%	8.15 [3.17, 20.95]	2023
**Total (95% CI)**		**39**		**41**	**100.0%**	**8.15 [3.17, 20.95]**	
Total events	31		4				

Heterogeneity: Not applicable
Test for overall effect: Z = 4.35 (P < 0.0001)

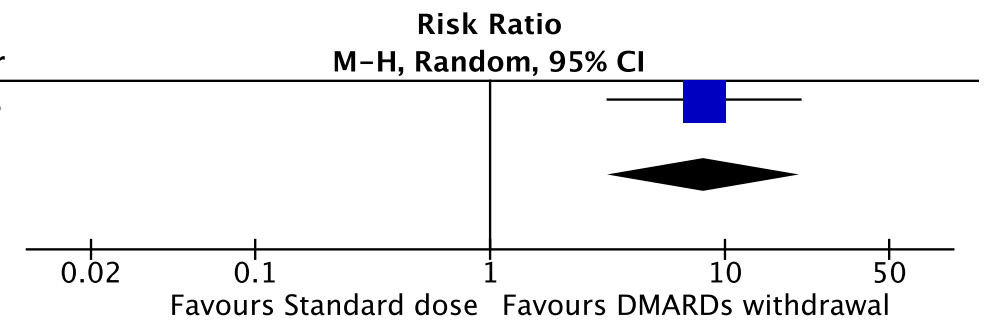

Figure S22. Only one study reported the short-term effect size of tsDMARD withdrawal on the risk of disease flare (RR 8.15, 95% CI 3.17–20.95; $p < 0.0001$).

Figure S23

a

Study or Subgroup	DMARDs withdrawal Events	DMARDs withdrawal Total	Standard DMARDs Events	Standard DMARDs Total	Weight	Risk Ratio M-H, Random, 95% CI	Year
Tascilar 2021	43	96	9	93	100.0%	4.63 [2.39, 8.95]	2021
**Total (95% CI)**		**96**		**93**	**100.0%**	**4.63 [2.39, 8.95]**	
Total events	43		9				

Heterogeneity: Not applicable
Test for overall effect: Z = 4.55 (P < 0.00001)

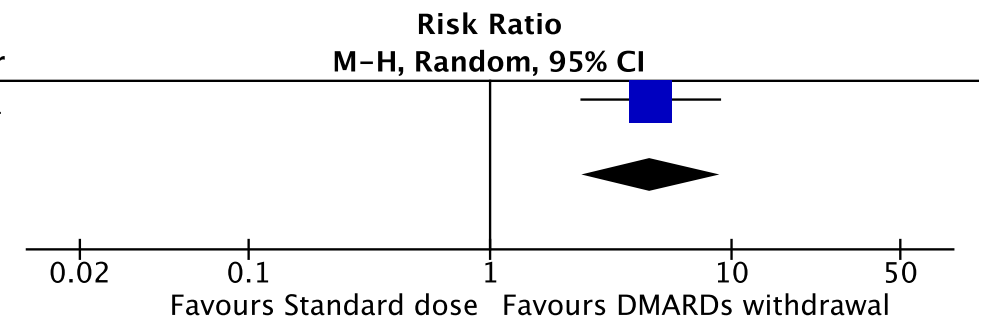

b

Study or Subgroup	DMARDs withdrawal Events	DMARDs withdrawal Total	Standard DMARDs Events	Standard DMARDs Total	Weight	Risk Ratio M-H, Random, 95% CI	Year
Tascilar 2021	53	96	16	93	61.2%	3.21 [1.98, 5.19]	2021
El Miedany 2016	24	32	2	32	38.8%	12.00 [3.09, 46.60]	2016
**Total (95% CI)**		**128**		**125**	**100.0%**	**5.35 [1.45, 19.79]**	
Total events	77		18				

Heterogeneity: $Tau^2 = 0.67$; $Chi^2 = 3.47$, df = 1 (P = 0.06); $I^2 = 71\%$
Test for overall effect: Z = 2.52 (P = 0.01)

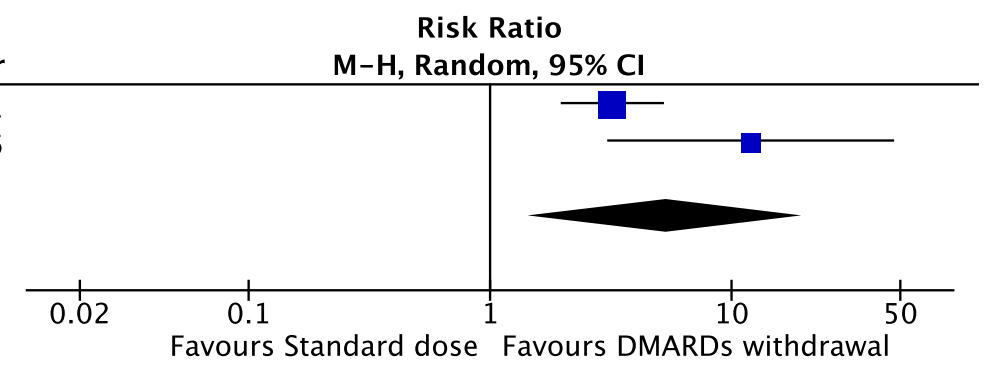

Figure S23. The short-term estimate (from one study) of flare risk following withdrawing all DMARDs: RR 4.63, 95% CI 2.39–8.95 (**a**); and the intermediate-term pooled estimate (from two studies): RR 5.35, 95% CI 1.45–19.79 (**b**).

Figure S24

a

Study or Subgroup	DMARDs tapering Events	DMARDs tapering Total	Standard DMARDs Events	Standard DMARDs Total	Weight	Risk Ratio M-H, Fixed, 95% CI	Year
Lillegraven 2021	14	75	5	73	50.3%	2.73 [1.03, 7.18]	2021
Smolen 2013	8	184	5	184	49.7%	1.60 [0.53, 4.80]	2013
**Total (95% CI)**		**259**		**257**	**100.0%**	**2.17 [1.05, 4.45]**	
Total events	22		10				

Heterogeneity: $Chi^2 = 0.51$, df = 1 (P = 0.48); $I^2 = 0\%$
Test for overall effect: Z = 2.10 (P = 0.04)

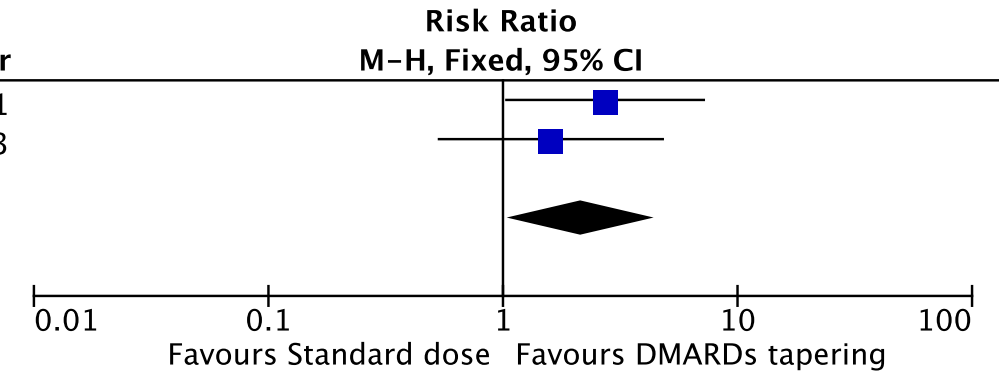

b

Study or Subgroup	DMARDs tapering Events	DMARDs tapering Total	Standard DMARDs Events	Standard DMARDs Total	Weight	Risk Ratio M-H, Fixed, 95% CI	Year
Kjorholt 2024	7	36	1	68	100.0%	13.22 [1.69, 103.32]	2024
**Total (95% CI)**		**36**		**68**	**100.0%**	**13.22 [1.69, 103.32]**	
Total events	7		1				

Heterogeneity: Not applicable
Test for overall effect: Z = 2.46 (P = 0.01)

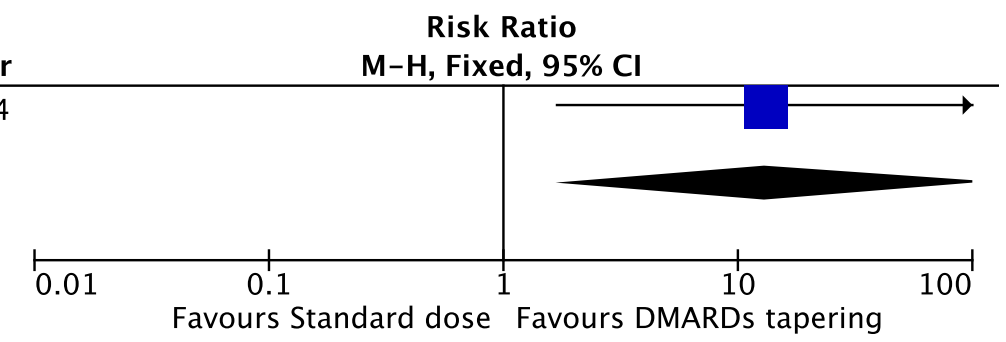

Figure S24. Pooled estimates of risk of radiographic progression following DMARD tapering in the intermediate term (**a**) and long term (**b**).

Figure S25

a

Study or Subgroup	DMARDs withdrawal Events	DMARDs withdrawal Total	Standard DMARDs Events	Standard DMARDs Total	Weight	Risk Ratio M-H, Fixed, 95% CI	Year
Ward 2025	0	52	1	27	20.8%	0.18 [0.01, 4.18]	2025
Lillegraven 2023	2	42	2	40	21.7%	0.95 [0.14, 6.44]	2023
van Herwaarden 2015	5	116	0	59	7.0%	5.64 [0.32, 100.32]	2015
Smolen 2013	18	167	5	184	50.5%	3.97 [1.51, 10.45]	2013
**Total (95% CI)**		**377**		**310**	**100.0%**	**2.64 [1.27, 5.49]**	
Total events	25		8				

Heterogeneity: $Chi^2 = 4.85$, df = 3 (P = 0.18); $I^2 = 38\%$
Test for overall effect: Z = 2.60 (P = 0.009)

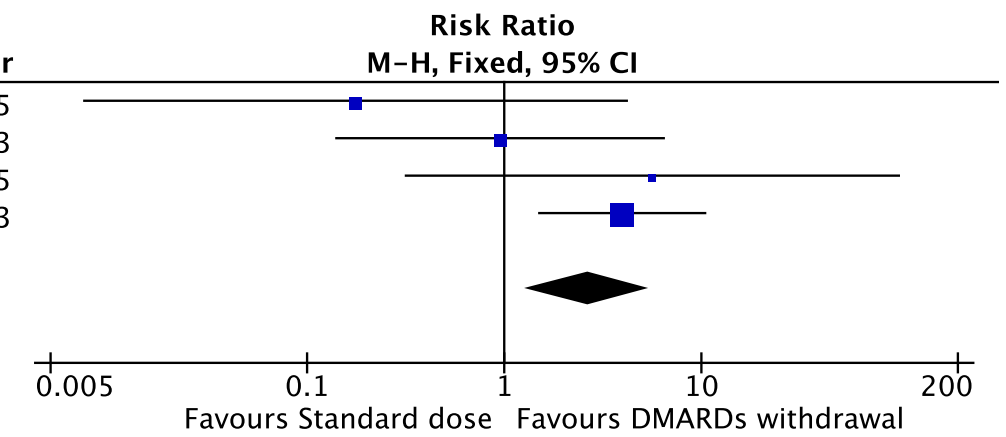

b

Study or Subgroup	DMARDs withdrawal Events	DMARDs withdrawal Total	Standard DMARDs Events	Standard DMARDs Total	Weight	Risk Ratio M-H, Fixed, 95% CI	Year
Kjorholt 2025	7	43	3	43	33.9%	2.33 [0.65, 8.43]	2025
Kedra 2024	8	28	5	34	51.1%	1.94 [0.72, 5.28]	2024
Kjorholt 2024	4	35	1	68	7.7%	7.77 [0.90, 66.92]	2024
Bouman 2017	5	101	0	55	7.3%	6.04 [0.34, 107.22]	2017
**Total (95% CI)**		**207**		**200**	**100.0%**	**2.82 [1.39, 5.75]**	
Total events	24		9				

Heterogeneity: $Chi^2 = 1.74$, df = 3 (P = 0.63); $I^2 = 0\%$
Test for overall effect: Z = 2.86 (P = 0.004)

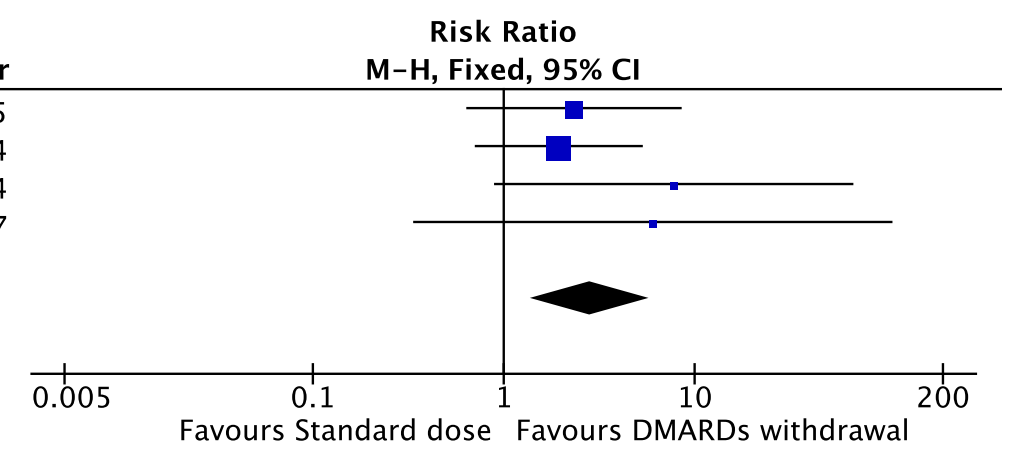

Figure S25. Forest plots of subgroup analysis showing the risk of radiographic progression associated with DMARD withdrawal versus maintaining standard doses across different follow-up durations. Pooled fixed-effect estimates support an increased risk of radiographic progression following DMARD withdrawal at both the intermedium term (**a**) (RR = 2.64, 95% CI 1.27–5.49, $p = 0.009$), and long term (**b**) (RR = 2.82, 95% CI 1.39–5.75, $p = 0.004$).

Figure S26

a

Study or Subgroup	DMARDs withdrawal Events	DMARDs withdrawal Total	Standard DMARDs Events	Standard DMARDs Total	Weight	Risk Ratio M-H, Fixed, 95% CI	Year
Ward 2025	0	52	1	27	0.0%	0.18 [0.01, 4.18]	2025
Lillegraven 2023	2	42	2	40	27.4%	0.95 [0.14, 6.44]	2023
van Herwaarden 2015	5	116	0	59	8.9%	5.64 [0.32, 100.32]	2015
Smolen 2013	18	167	5	184	63.7%	3.97 [1.51, 10.45]	2013
**Total (95% CI)**		**325**		**283**	**100.0%**	**3.29 [1.47, 7.36]**	
Total events	25		7				

Heterogeneity: $Chi^2 = 1.89$, df = 2 (P = 0.39); $I^2 = 0\%$
Test for overall effect: Z = 2.89 (P = 0.004)

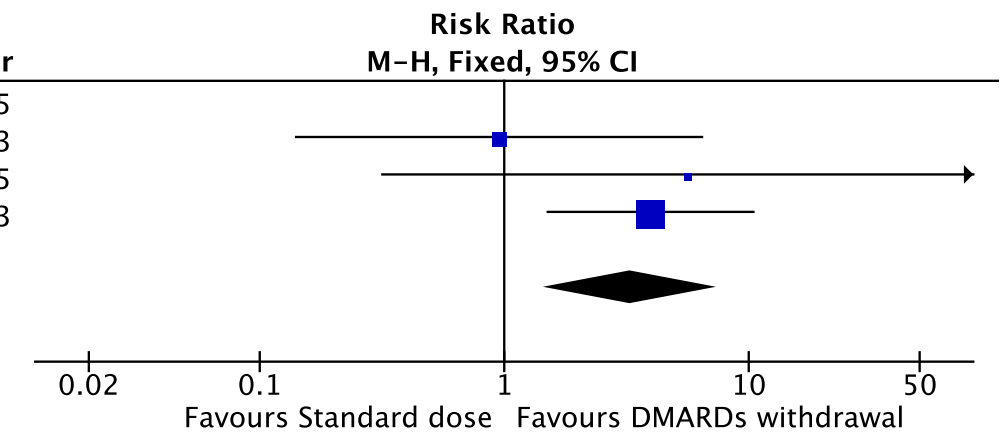

b

Study or Subgroup	DMARDs withdrawal Events	DMARDs withdrawal Total	Standard DMARDs Events	Standard DMARDs Total	Weight	Risk Ratio M-H, Random, 95% CI	Year
Kjorholt 2025	7	43	3	43	31.2%	2.33 [0.65, 8.43]	2025
Kedra 2024	8	28	5	34	51.5%	1.94 [0.72, 5.28]	2024
Kjorholt 2024	4	35	1	68	11.1%	7.77 [0.90, 66.92]	2024
Bouman 2017	5	101	0	55	6.2%	6.04 [0.34, 107.22]	2017
**Total (95% CI)**		**207**		**200**	**100.0%**	**2.57 [1.26, 5.27]**	
Total events	24		9				

Heterogeneity: $Tau^2 = 0.00$; $Chi^2 = 1.74$, df = 3 (P = 0.63); $I^2 = 0\%$
Test for overall effect: Z = 2.58 (P = 0.010)

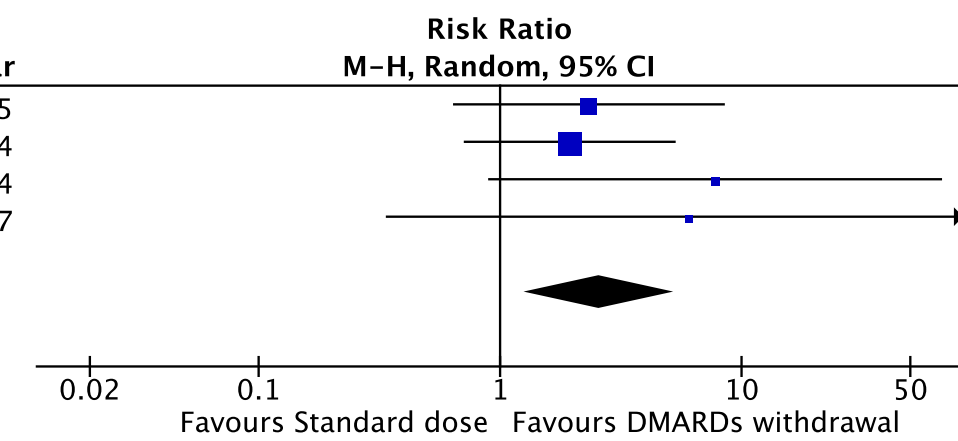

c

Study or Subgroup	DMARDs withdrawal Events	DMARDs withdrawal Total	Standard DMARDs Events	Standard DMARDs Total	Weight	Risk Ratio M-H, Fixed, 95% CI	Year
Kjorholt 2025	7	43	3	43	69.4%	2.33 [0.65, 8.43]	2025
Kedra 2024	8	28	5	34	0.0%	1.94 [0.72, 5.28]	2024
Kjorholt 2024	4	35	1	68	15.7%	7.77 [0.90, 66.92]	2024
Bouman 2017	5	101	0	55	14.9%	6.04 [0.34, 107.22]	2017
**Total (95% CI)**		**179**		**166**	**100.0%**	**3.74 [1.36, 10.32]**	
Total events	16		4				

Heterogeneity: $Chi^2 = 1.07$, df = 2 (P = 0.59); $I^2 = 0\%$
Test for overall effect: Z = 2.55 (P = 0.01)

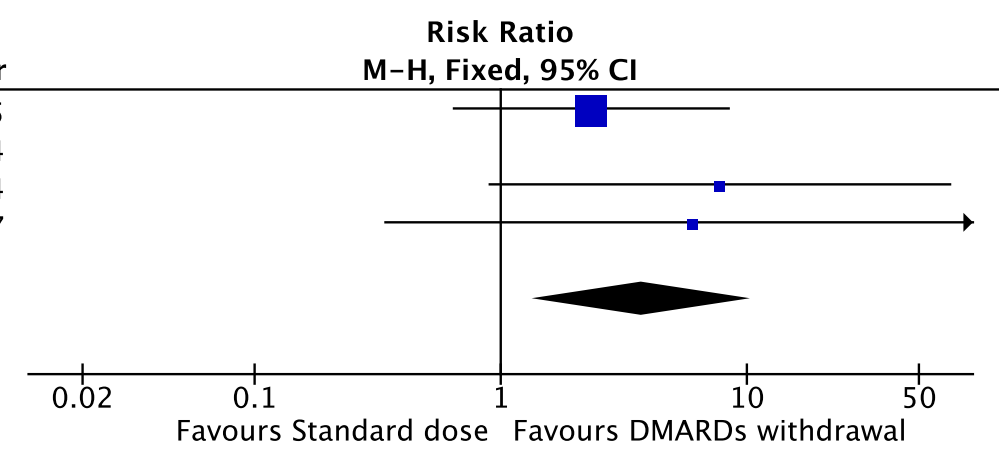

Figure S26. Sensitivity analyses confirmed the robustness of the finding that DMARD withdrawal was associated with increased risk of radiographic progression. **a**. leave-one-out (LOO) sensitivity analysis eliminated heterogeneity and maintained the direction and statistical significance on the risk of radiographic progression in the intermediate term (RR 3.29, 95% CI 1.47–7.36; $I^2 = 0\%$; $p = 0.004$); **b**. sensitivity analysis using a random-effects model yielded consistent significance on the increased risk of radiographic progression in the long term (RR 2.57, 95% CI 1.26–5.27; $I^2 = 0\%$; $p = 0.01$); **c**. Excluding the highest-weight study maintained the significance on the increased risk of radiographic progression in the long term (RR 3.74, 95% CI 1.36–10.32; $I^2 = 0\%$; $p = 0.01$).

Figure S27

a

Study or Subgroup	DMARDs tapering Events	DMARDs tapering Total	Standard DMARDs Events	Standard DMARDs Total	Weight	Risk Ratio M-H, Random, 95% CI	Year
Kjorholt 2024	36	40	65	78	26.4%	1.08 [0.94, 1.25]	2024
Uhrenholt 2023	31	41	12	20	3.4%	1.26 [0.85, 1.88]	2023
Wang 2023	10	42	14	41	1.1%	0.70 [0.35, 1.39]	2023
Bertrand 2022	16	32	19	34	2.6%	0.89 [0.57, 1.41]	2022
Lillegraven 2021	34	78	42	78		Not estimable	2021
Sanmarti 2019	63	90	50	89	10.4%	1.25 [0.99, 1.56]	2019
Takeuchi 2019	120	245	123	245	17.0%	0.98 [0.82, 1.17]	2019
Weinblatt 2017	81	127	53	83	12.5%	1.00 [0.81, 1.23]	2017
van Vollenhoven 2016	20	27	16	23	4.4%	1.06 [0.75, 1.51]	2016
Smolen 2013	122	202	124	202	22.2%	0.98 [0.84, 1.15]	2013
**Total (95% CI)**		**846**		**815**	**100.0%**	**1.04 [0.97, 1.12]**	
Total events	499		476				

Heterogeneity: $Tau^2 = 0.00$; $Chi^2 = 6.66$, df = 8 (P = 0.57); $I^2 = 0\%$
Test for overall effect: Z = 1.04 (P = 0.30)

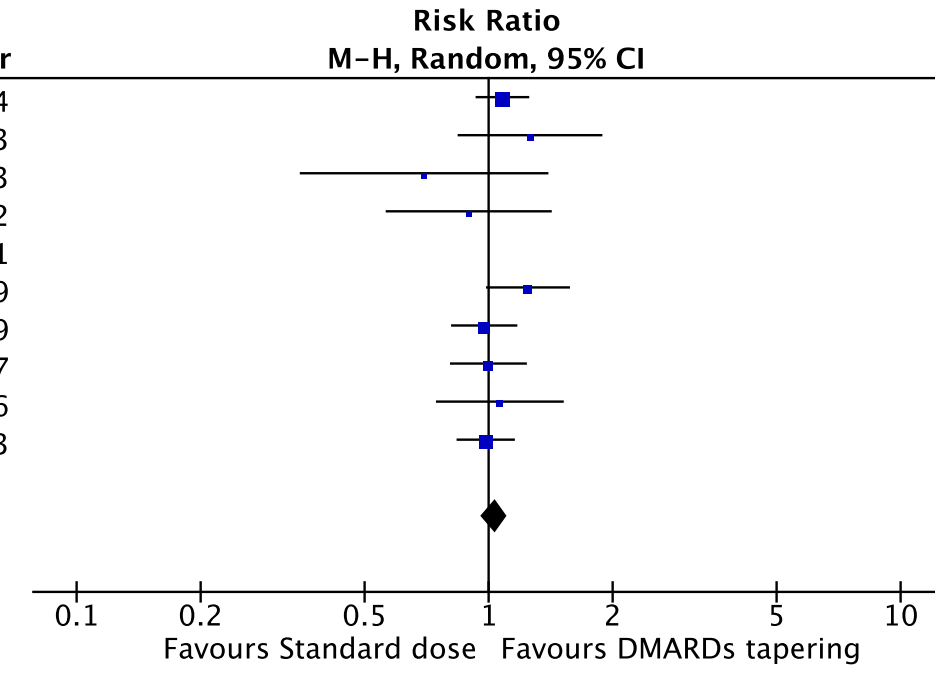

b

Study or Subgroup	DMARDs withdrawal Events	DMARDs withdrawal Total	Standard DMARDs Events	Standard DMARDs Total	Weight	Risk Ratio M-H, Random, 95% CI	Year
Kjorholt 2025	39	47	40	45	9.8%	0.93 [0.79, 1.10]	2025
Ward 2025	35	69	13	33	2.9%	1.29 [0.79, 2.09]	2025
Asai 2025	5	43	7	39	0.7%	0.65 [0.22, 1.87]	2025
Kedra 2024	82	113	78	115	9.6%	1.07 [0.90, 1.27]	2024
Kjorholt 2024	36	37	65	78	11.8%	1.17 [1.04, 1.31]	2024
Lillegraven 2023	23	47	31	45		Not estimable	2023
Wang 2023	3	39	14	41	0.6%	0.23 [0.07, 0.72]	2023
Emery 2023	32	50	22	50	4.2%	1.45 [1.00, 2.12]	2023
Curtis 2021	63	100	33	53	6.7%	1.01 [0.78, 1.31]	2021
Bouman 2017	104	115	54	57	12.8%	0.95 [0.88, 1.04]	2017
Weinblatt 2017	48	81	53	83	7.2%	0.93 [0.73, 1.18]	2017
Fautrel 2016	46	64	51	73	8.0%	1.03 [0.83, 1.28]	2016
van Vollenhoven 2016	7	23	16	23	1.6%	0.44 [0.22, 0.86]	2016
Chatzidionysiou 2016	15	15	14	16	8.0%	1.14 [0.92, 1.41]	2016
Yamanaka 2016	9	40	15	45	1.5%	0.68 [0.33, 1.37]	2016
Moghadam 2016	95	531	43	286	5.0%	1.19 [0.86, 1.66]	2016
van Herwaarden 2015	95	121	45	59		Not estimable	2015
Smolen 2013	105	200	124	202	9.6%	0.86 [0.72, 1.02]	2013
**Total (95% CI)**		**1567**		**1239**	**100.0%**	**1.01 [0.92, 1.10]**	
Total events	724		642				

Heterogeneity: $Tau^2 = 0.02$; $Chi^2 = 35.58$, df = 15 (P = 0.002); $I^2 = 58\%$
Test for overall effect: Z = 0.12 (P = 0.91)

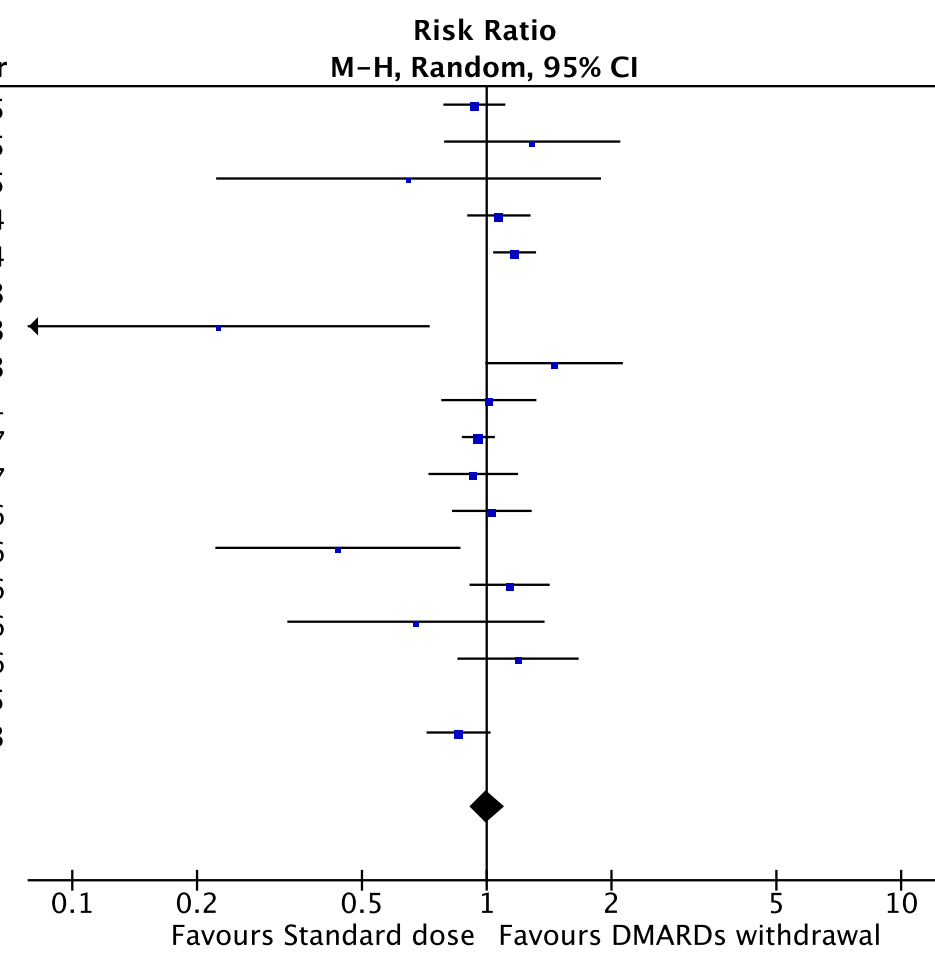

Figure S27. Forest plots of subgroup analysis showing the risk of any adverse events upon DMARD tapering (**a**) or withdrawal (**b**) compared with full-dose treatments. Pooled random-effects estimates indicate that neither DMARD tapering (RR = 1.04, 95% CI 0.97–1.12, $p = 0.30$), nor withdrawal (RR = 1.01, 95% CI 0.92–1.10, $p = 0.91$) was associated with changes of any adverse events.

Figure S28

a

Study or Subgroup	DMARDs tapering Events	DMARDs tapering Total	Standard DMARDs Events	Standard DMARDs Total	Weight	Risk Ratio M-H, Random, 95% CI	Year
Kjorholt 2024	20	40	40	78	15.2%	0.97 [0.67, 1.42]	2024
Uhrenholt 2023	28	41	10	20	10.2%	1.37 [0.84, 2.22]	2023
Wang 2023	1	42	4	41	0.6%	0.24 [0.03, 2.09]	2023
Bertrand 2022	7	32	9	34	3.6%	0.83 [0.35, 1.96]	2022
Lillgraven 2021	17	78	21	78	0.0%	0.81 [0.46, 1.41]	2021
Samarti 2019	27	90	25	89	11.2%	1.07 [0.68, 1.69]	2019
Takeuchi 2019	59	278	78	281	21.6%	0.76 [0.57, 1.03]	2019
Weinblatt 2017	49	127	26	83	14.7%	1.23 [0.84, 1.81]	2017
Smolen 2013	60	202	72	202	23.0%	0.83 [0.63, 1.10]	2013
**Total (95% CI)**		**852**		**828**	**100.0%**	**0.95 [0.80, 1.13]**	
Total events	251		264				

Heterogeneity: $Tau^2 = 0.01$; $Chi^2 = 8.77$, df = 7 (P = 0.27); $I^2 = 20\%$
Test for overall effect: Z = 0.58 (P = 0.56)

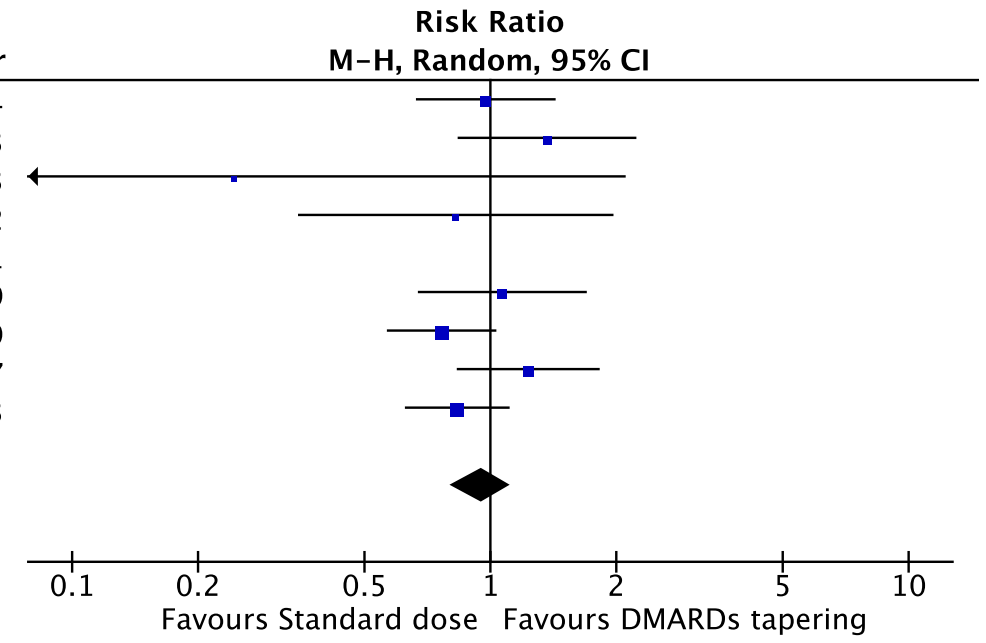

b

Study or Subgroup	DMARDs tapering Events	DMARDs tapering Total	Standard DMARDs Events	Standard DMARDs Total	Weight	Odds Ratio M-H, Random, 95% CI	Year
Kjorholt 2024	20	40	40	78	9.7%	0.95 [0.44, 2.04]	2024
Uhrenholt 2023	28	41	10	20	5.0%	2.15 [0.72, 6.44]	2023
Wang 2023	1	42	4	41	1.3%	0.23 [0.02, 2.11]	2023
Bertrand 2022	7	32	9	34	4.7%	0.78 [0.25, 2.41]	2022
Lillgraven 2021	17	78	21	78	0.0%	0.81 [0.46, 1.41]	2021
Samarti 2019	27	90	25	89	12.8%	1.10 [0.58, 2.09]	2019
Takeuchi 2019	59	278	78	281	26.9%	0.70 [0.48, 1.03]	2019
Weinblatt 2017	49	127	26	83	15.0%	1.38 [0.77, 2.47]	2017
Smolen 2013	60	202	72	202	24.5%	0.76 [0.50, 1.16]	2013
**Total (95% CI)**		**852**		**828**	**100.0%**	**0.91 [0.70, 1.17]**	
Total events	251		264				

Heterogeneity: $Tau^2 = 0.02$; $Chi^2 = 8.53$, df = 7 (P = 0.29); $I^2 = 18\%$
Test for overall effect: Z = 0.76 (P = 0.45)

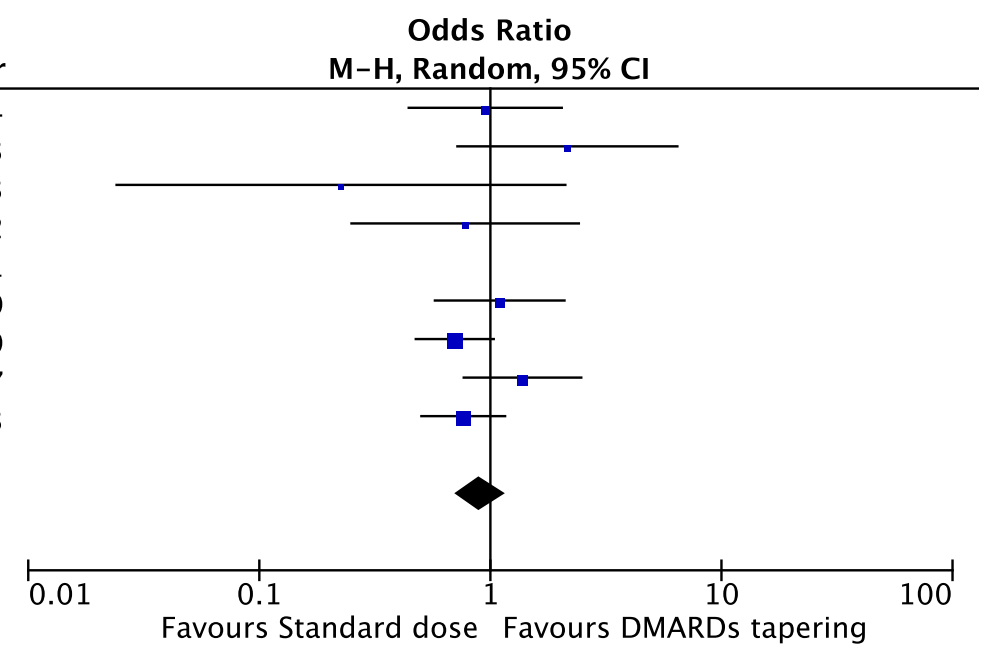

Figure S28. Forest plots showing that the risk of infections did not change upon DMARD tapering (pooled RR 0.95, 95% CI 0.80–1.13; $p = 0.56$) with low heterogeneity (n = 0.01, $I^2 = 20\%$) (**a**), which was further validated by sensitivity analyses using OR as effect size (**b**).

Figure S29

Study or Subgroup	DMARDs tapering Events	DMARDs tapering Total	Standard DMARDs Events	Standard DMARDs Total	Weight	Risk Ratio M-H, Random, 95% CI	Year
**5.2.1 bDMARDs**							
Uhrenholt 2023	31	41	12	20	3.4%	1.26 [0.85, 1.88]	2023
Bertrand 2022	16	32	19	34	2.6%	0.89 [0.57, 1.41]	2022
Sanmarti 2019	63	90	50	89	10.4%	1.25 [0.99, 1.56]	2019
Weinblatt 2017	81	127	53	83	12.5%	1.00 [0.81, 1.23]	2017
van Vollenhoven 2016	20	27	16	23	4.4%	1.06 [0.75, 1.51]	2016
Smolen 2013	122	202	124	202	22.2%	0.98 [0.84, 1.15]	2013
**Subtotal (95% CI)**		**519**		**451**	**55.5%**	**1.05 [0.95, 1.16]**	
Total events	333		274				
Heterogeneity: Tau^2 = 0.00; Chi^2 = 4.34, df = 5 (P = 0.50); I^2 = 0%							
Test for overall effect: Z = 0.96 (P = 0.33)							
**5.2.2 csDMARDs**							
Kjorholt 2024	36	40	65	78	26.4%	1.08 [0.94, 1.25]	2024
Lillegraven 2021	34	78	42	78		Not estimable	2021
**Subtotal (95% CI)**		**40**		**78**	**26.4%**	**1.08 [0.94, 1.25]**	
Total events	36		65				
Heterogeneity: Not applicable							
Test for overall effect: Z = 1.05 (P = 0.29)							
**5.2.3 tsDMARDs**							
Wang 2023	10	42	14	41	1.1%	0.70 [0.35, 1.39]	2023
Takeuchi 2019	120	245	123	245	17.0%	0.98 [0.82, 1.17]	2019
**Subtotal (95% CI)**		**287**		**286**	**18.1%**	**0.96 [0.80, 1.14]**	
Total events	130		137				
Heterogeneity: Tau^2 = 0.00; Chi^2 = 0.87, df = 1 (P = 0.35); I^2 = 0%							
Test for overall effect: Z = 0.52 (P = 0.60)							
**5.2.4 all DMARDs**							
**Subtotal (95% CI)**		**0**		**0**		**Not estimable**	
Total events	0		0				
Heterogeneity: Not applicable							
Test for overall effect: Not applicable							
**Total (95% CI)**		**846**		**815**	**100.0%**	**1.04 [0.97, 1.12]**	
Total events	499		476				
Heterogeneity: Tau^2 = 0.00; Chi^2 = 6.66, df = 8 (P = 0.57); I^2 = 0%							
Test for overall effect: Z = 1.04 (P = 0.30)							
Test for subgroup differences: Chi^2 = 1.23, df = 2 (P = 0.54), I^2 = 0%							

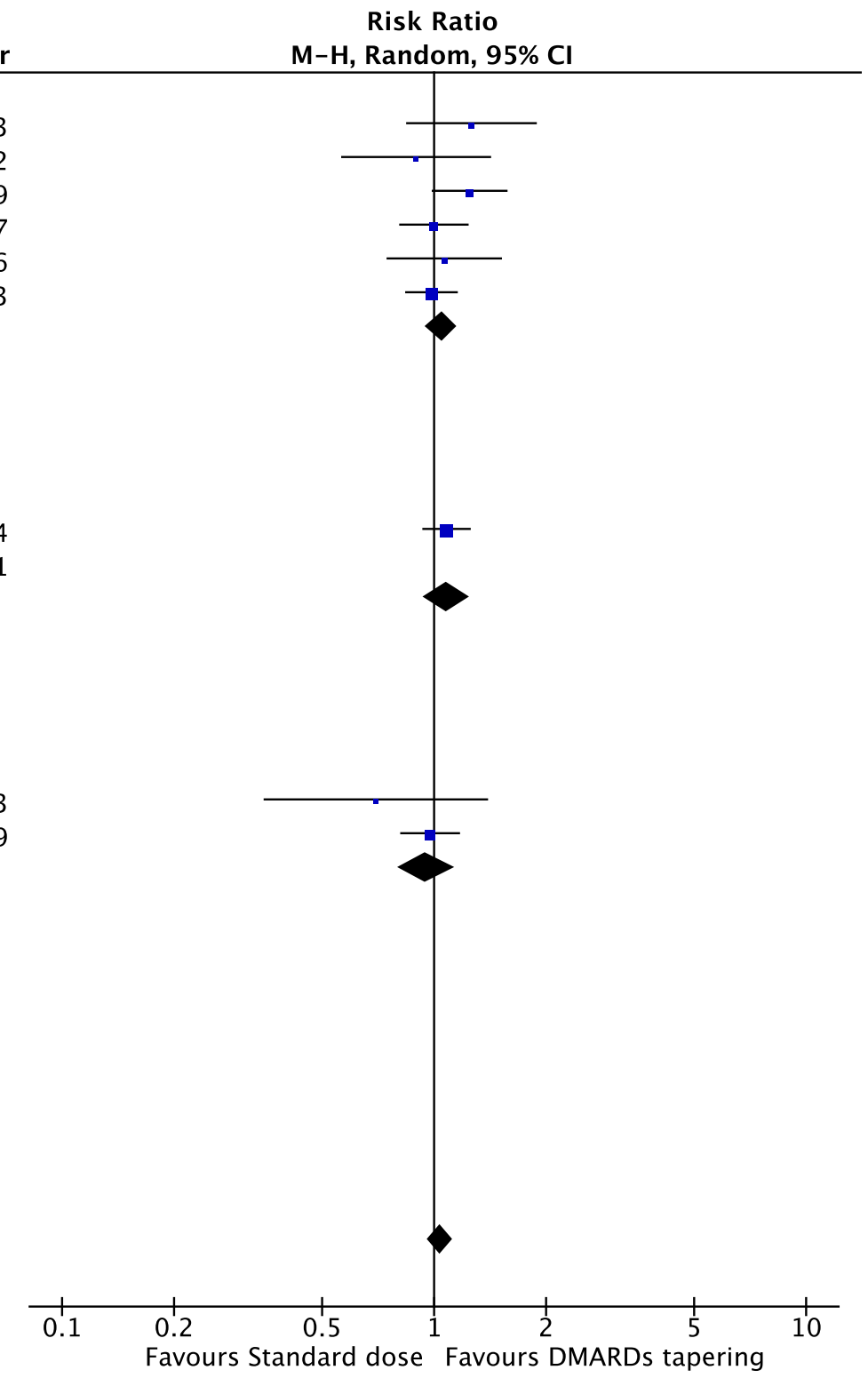

Figure S29. Subgroup analysis revealed that tapering was not associated with an increased risk of any adverse events overall (pooled RR 1.04, 95% CI 0.97–1.12; p = 0.30) or in any subgroups, and no heterogeneity was detected ($\tau^2 = 0$, $I^2 = 0\%$).

Figure S30

a

Study or Subgroup	DMARDs tapering Events	Total	Standard DMARDs Events	Total	Weight	Risk Ratio M–H, Fixed, 95% CI	Year
Kjorholt 2024	4	40	13	78	12.8%	0.60 [0.21, 1.72]	2024
Wang 2023	0	42	1	41	2.2%	0.33 [0.01, 7.77]	2023
Uhrenholt 2023	3	41	2	20	3.9%	0.73 [0.13, 4.04]	2023
Bertrand 2022	7	32	4	34	5.6%	1.86 [0.60, 5.75]	2022
Lillegraven 2021	4	78	2	78	2.9%	2.00 [0.38, 10.60]	2021
Tascilar 2021	7	93	10	93	14.5%	0.70 [0.28, 1.76]	2021
Verhoef 2019	6	58	3	29	5.8%	1.00 [0.27, 3.71]	2019
Sanmarti 2019	1	90	2	89	2.9%	0.49 [0.05, 5.36]	2019
Takeuchi 2019	15	245	16	245	23.2%	0.94 [0.47, 1.85]	2019
Weinblatt 2017	9	127	4	83	7.0%	1.47 [0.47, 4.62]	2017
van Vollenhoven 2016	1	27	1	23	1.6%	0.85 [0.06, 12.87]	2016
Smolen 2013	7	202	12	202	17.4%	0.58 [0.23, 1.45]	2013
**Total (95% CI)**		**1075**		**1015**	**100.0%**	**0.89 [0.64, 1.24]**	
Total events	64		70				

Heterogeneity: Chi² = 5.63, df = 11 (P = 0.90); I² = 0%
Test for overall effect: Z = 0.71 (P = 0.48)

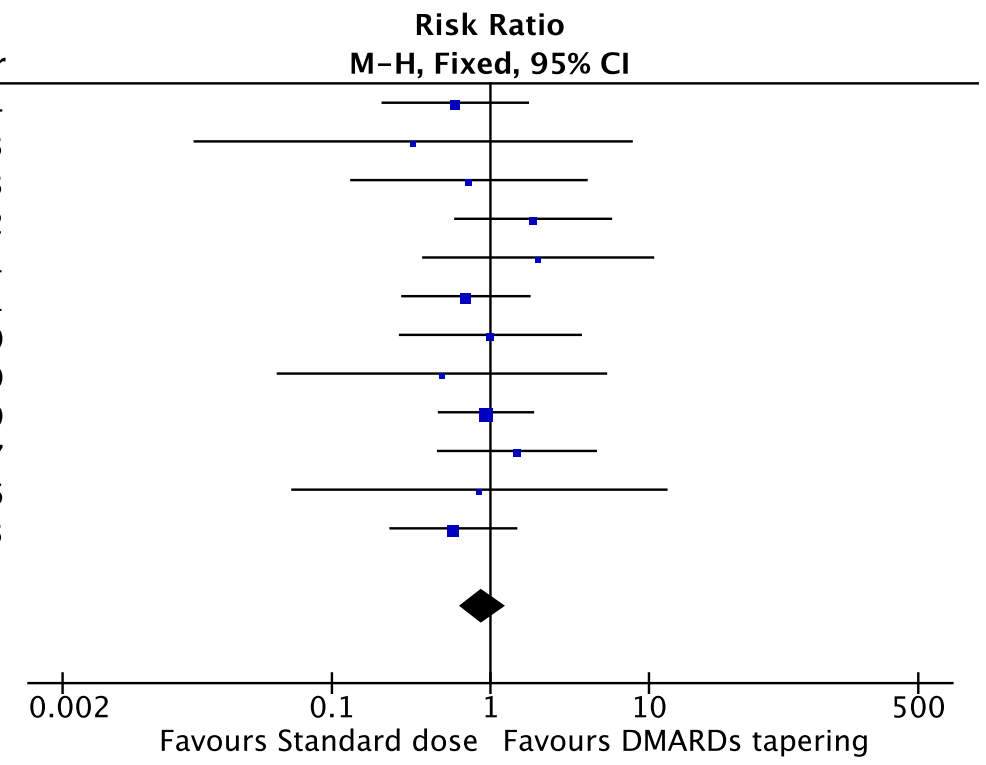

b

Study or Subgroup	DMARDs withdrawal Events	Total	Standard DMARDs Events	Total	Weight	Risk Ratio M–H, Fixed, 95% CI	Year
Asai 2025	5	43	0	39	0.5%	10.00 [0.57, 175.18]	2025
Kjorholt 2025	10	47	5	45	4.5%	1.91 [0.71, 5.17]	2025
Ward 2025	8	69	4	33	4.8%	0.96 [0.31, 2.95]	2025
Kjorholt 2024	6	37	13	78	7.4%	0.97 [0.40, 2.36]	2024
Kedra 2024	12	113	18	115	15.8%	0.68 [0.34, 1.34]	2024
Lillegraven 2023	3	47	2	45		Not estimable	2023
Wang 2023	0	39	1	41	1.3%	0.35 [0.01, 8.34]	2023
Emery 2023	3	50	3	50	2.7%	1.00 [0.21, 4.72]	2023
Tascilar 2021	13	96	10	93	9.0%	1.26 [0.58, 2.73]	2021
Curtis 2021	4	100	3	53	3.5%	0.71 [0.16, 3.04]	2021
Bouman 2017	50	115	15	57	17.8%	1.65 [1.02, 2.67]	2017
Weinblatt 2017	6	81	4	83	3.5%	1.54 [0.45, 5.25]	2017
Fautrel 2016	6	64	10	73	8.3%	0.68 [0.26, 1.78]	2016
van Vollenhoven 2016	0	23	1	23	1.3%	0.33 [0.01, 7.78]	2016
Chatzidionysiou 2016	3	15	1	16	0.9%	3.20 [0.37, 27.49]	2016
Moghadam 2016	34	531	7	286	8.1%	2.62 [1.17, 5.83]	2016
van Herwaarden 2015	30	121	7	59		Not estimable	2015
Smolen 2013	15	200	12	202	10.6%	1.26 [0.61, 2.63]	2013
**Total (95% CI)**		**1623**		**1287**	**100.0%**	**1.31 [1.04, 1.65]**	
Total events	175		107				

Heterogeneity: Chi² = 15.27, df = 15 (P = 0.43); I² = 2%
Test for overall effect: Z = 2.28 (P = 0.02)

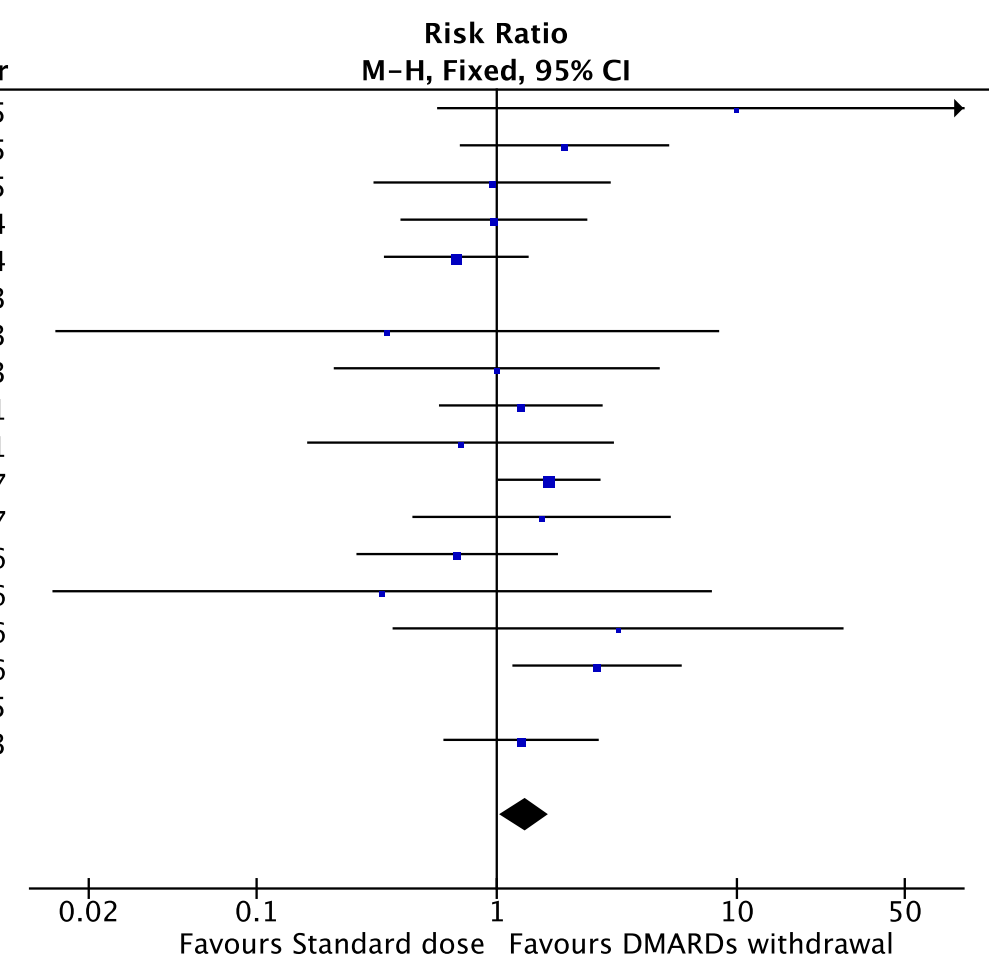

Figure S30. Forest plots of subgroup analysis showing the risk of serious adverse events upon DMARD tapering (**a**) or withdrawal (**b**) compared with full-dose treatments. Pooled fixed-effect estimates indicate that DMARD tapering was not associated with the risk of serious adverse events (RR = 0.89, 95% CI 0.64–1.24, $p = 0.48$), but DMARD withdrawal was associated (RR = 1.31, 95% CI 1.04–1.65, $p = 0.02$).

Figure S31

Study or Subgroup	DMARDs tapering Events	DMARDs tapering Total	Standard DMARDs Events	Standard DMARDs Total	Weight	Risk Ratio M–H, Fixed, 95% CI	Year
**7.2.1 bDMARDs**							
Uhrenholt 2023	3	41	2	20	4.0%	0.73 [0.13, 4.04]	2023
Bertrand 2022	7	32	4	34	5.8%	1.86 [0.60, 5.75]	2022
Verhoef 2019	6	58	3	29	6.0%	1.00 [0.27, 3.71]	2019
Sanmarti 2019	1	90	2	89	3.0%	0.49 [0.05, 5.36]	2019
Weinblatt 2017	9	127	4	83	7.2%	1.47 [0.47, 4.62]	2017
van Vollenhoven 2016	1	27	1	23	1.6%	0.85 [0.06, 12.87]	2016
Smolen 2013	7	202	12	202	18.0%	0.58 [0.23, 1.45]	2013
**Subtotal (95% CI)**		**577**		**480**	**45.6%**	**0.96 [0.59, 1.56]**	
Total events	34		28				
Heterogeneity: Chi² = 3.40, df = 6 (P = 0.76); I² = 0%							
Test for overall effect: Z = 0.17 (P = 0.86)							
**7.2.2 csDMARDs**							
Kjorholt 2024	4	40	13	78	13.2%	0.60 [0.21, 1.72]	2024
Lillegraven 2021	4	78	2	78		Not estimable	2021
**Subtotal (95% CI)**		**40**		**78**	**13.2%**	**0.60 [0.21, 1.72]**	
Total events	4		13				
Heterogeneity: Not applicable							
Test for overall effect: Z = 0.95 (P = 0.34)							
**7.2.3 tsDMARDs**							
Wang 2023	0	42	1	41	2.3%	0.33 [0.01, 7.77]	2023
Takeuchi 2019	15	245	16	245	23.9%	0.94 [0.47, 1.85]	2019
**Subtotal (95% CI)**		**287**		**286**	**26.2%**	**0.88 [0.46, 1.72]**	
Total events	15		17				
Heterogeneity: Chi² = 0.41, df = 1 (P = 0.52); I² = 0%							
Test for overall effect: Z = 0.36 (P = 0.72)							
**7.2.4 all DMARDs**							
Tascilar 2021	7	93	10	93	15.0%	0.70 [0.28, 1.76]	2021
**Subtotal (95% CI)**		**93**		**93**	**15.0%**	**0.70 [0.28, 1.76]**	
Total events	7		10				
Heterogeneity: Not applicable							
Test for overall effect: Z = 0.76 (P = 0.45)							
**Total (95% CI)**		**997**		**937**	**100.0%**	**0.85 [0.61, 1.20]**	
Total events	60		68				
Heterogeneity: Chi² = 4.69, df = 10 (P = 0.91); I² = 0%							
Test for overall effect: Z = 0.92 (P = 0.36)							
Test for subgroup differences: Chi² = 0.83, df = 3 (P = 0.84), I² = 0%							

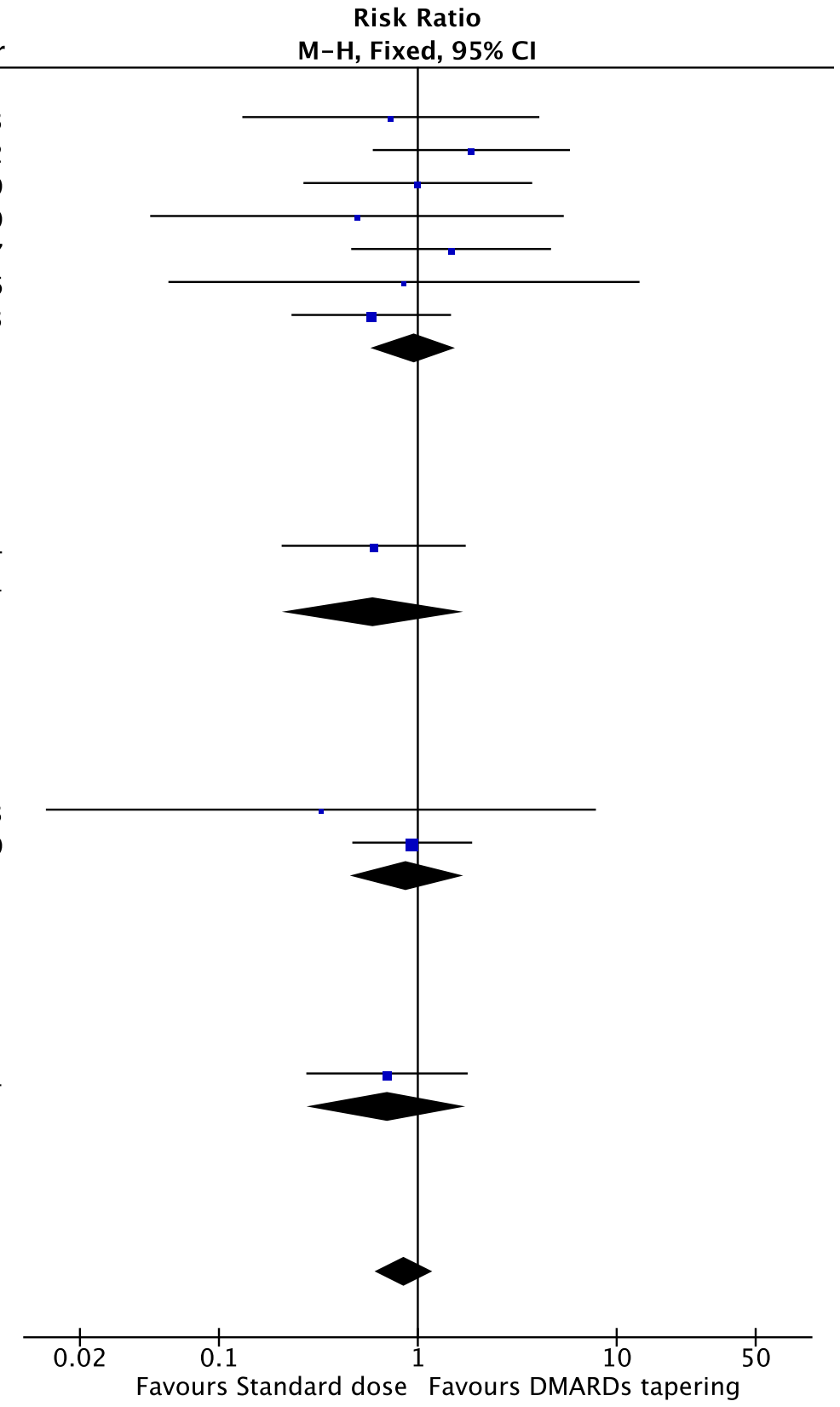

Figure S31. Subgroup analysis revealed that tapering was not associated with an increased risk of serious adverse events overall (RR 0.85, 95% CI 0.61–1.20; *p* = 0.36) or in any subgroups, and no heterogeneity was detected ($I^2 = 0\%$).

Figure S32

a

Study or Subgroup	DMARDs withdrawal Events	DMARDs withdrawal Total	Standard DMARDs Events	Standard DMARDs Total	Weight	Risk Ratio M–H, Random, 95% CI	Year
Kjorholt 2025	39	47	40	45	11.9%	0.93 [0.79, 1.10]	2025
Ward 2025	35	69	13	33	2.6%	1.29 [0.79, 2.09]	2025
Kedra 2024	82	113	78	115	11.6%	1.07 [0.90, 1.27]	2024
Lillegraven 2023	23	47	31	45	0.0%	0.71 [0.50, 1.01]	2023
Emery 2023	32	50	22	50	3.9%	1.45 [1.00, 2.12]	2023
Curtis 2021	63	100	33	53	7.0%	1.01 [0.78, 1.31]	2021
Bouman 2017	104	115	54	57	18.5%	0.95 [0.88, 1.04]	2017
Weinblatt 2017	48	81	53	83	7.6%	0.93 [0.73, 1.18]	2017
Fautrel 2016	46	64	51	73	8.9%	1.03 [0.83, 1.28]	2016
van Vollenhoven 2016	7	23	16	23	1.4%	0.44 [0.22, 0.86]	2016
Chatzidionysiou 2016	15	15	14	16	8.9%	1.14 [0.92, 1.41]	2016
Yamanaka 2016	9	40	15	45	1.3%	0.68 [0.33, 1.37]	2016
Moghadam 2016	95	531	43	286	4.9%	1.19 [0.86, 1.66]	2016
van Herwaarden 2015	95	121	45	59	0.0%	1.03 [0.87, 1.22]	2015
Smolen 2013	105	200	124	202	11.5%	0.86 [0.72, 1.02]	2013
**Total (95% CI)**		**1448**		**1081**	**100.0%**	**1.00 [0.92, 1.08]**	
Total events	680		556				

Heterogeneity: Tau^2 = 0.01; Chi^2 = 19.87, df = 12 (P = 0.07); I^2 = 40%
Test for overall effect: Z = 0.11 (P = 0.91)

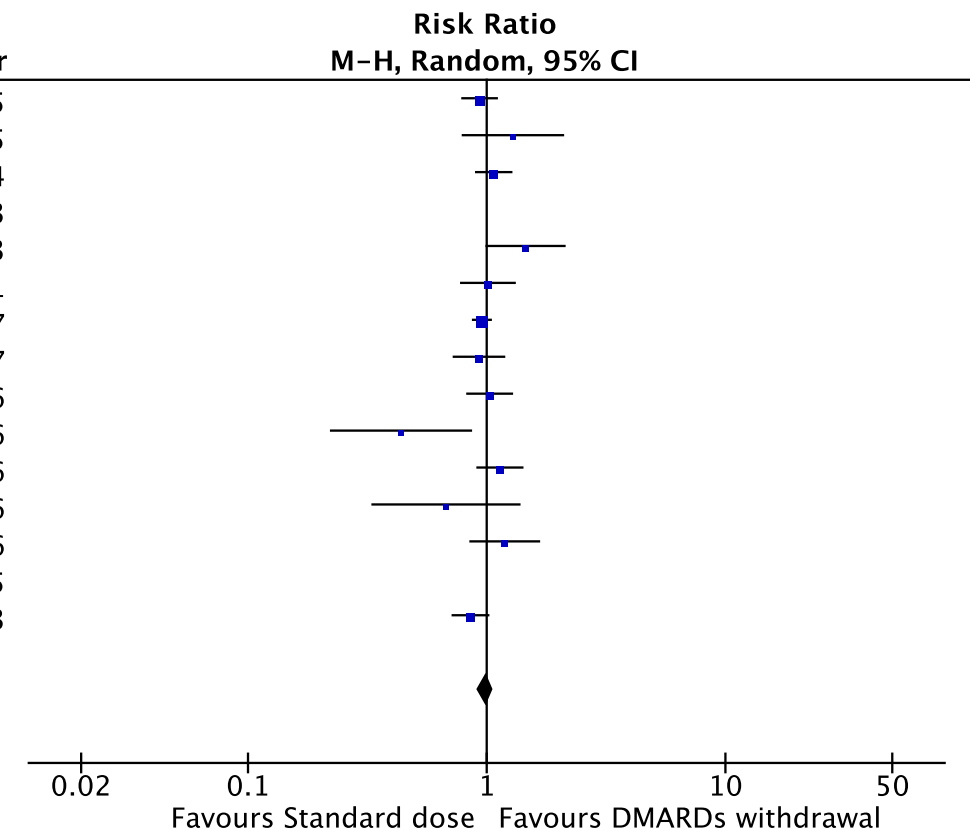

b

Study or Subgroup	DMARDs withdrawal Events	DMARDs withdrawal Total	Standard DMARDs Events	Standard DMARDs Total	Weight	Risk Ratio M–H, Random, 95% CI	Year
Asai 2025	5	43	7	39	4.7%	0.65 [0.22, 1.87]	2025
Kjorholt 2024	36	37	65	78	45.0%	1.17 [1.04, 1.31]	2024
Emery 2023	24	47	22	50	19.9%	1.16 [0.76, 1.77]	2023
Curtis 2021	55	99	33	53	30.4%	0.89 [0.68, 1.17]	2021
**Total (95% CI)**		**226**		**220**	**100.0%**	**1.05 [0.82, 1.33]**	
Total events	120		127				

Heterogeneity: Tau^2 = 0.03; Chi^2 = 6.94, df = 3 (P = 0.07); I^2 = 57%
Test for overall effect: Z = 0.36 (P = 0.72)

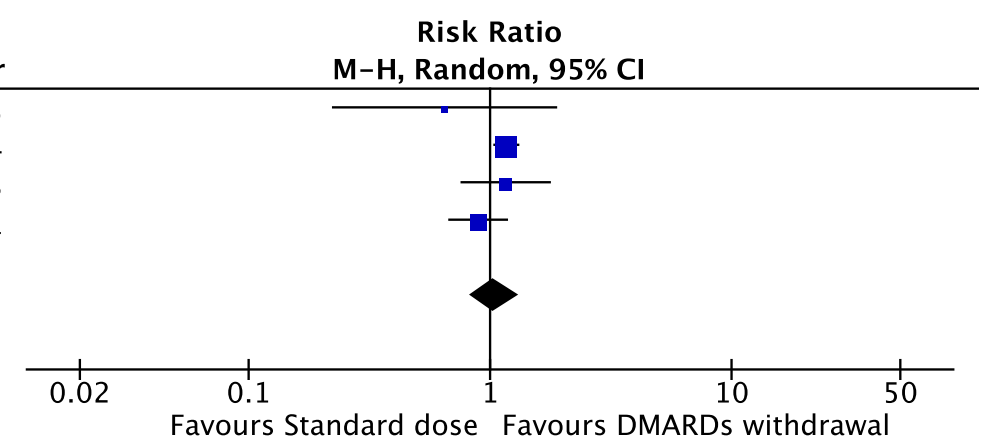

c

Study or Subgroup	DMARDs withdrawal Events	DMARDs withdrawal Total	Standard DMARDs Events	Standard DMARDs Total	Weight	Risk Ratio M–H, Random, 95% CI	Year
Wang 2023	3	39	14	41	100.0%	0.23 [0.07, 0.72]	2023
**Total (95% CI)**		**39**		**41**	**100.0%**	**0.23 [0.07, 0.72]**	
Total events	3		14				

Heterogeneity: Not applicable
Test for overall effect: Z = 2.50 (P = 0.01)

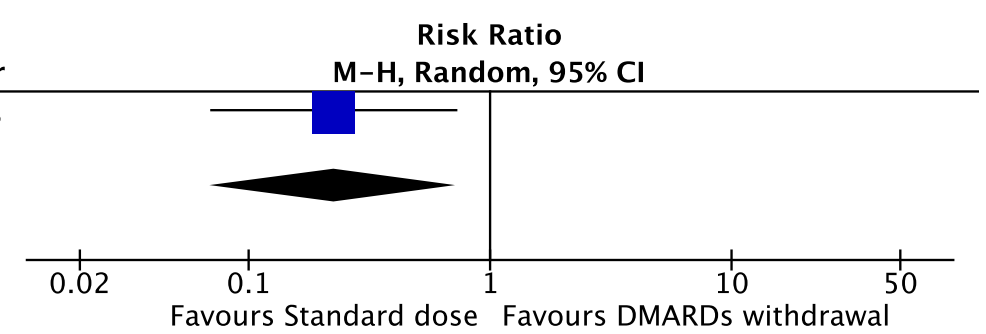

Figure S32. Subgroup analysis revealed that withdrawing bDMARDs (**a**) or csDMARDs (**b**) did not alter the risk of any adverse events; withdrawing bDMARDs: RR 1.00, 95% CI 0.92–1.08; I^2 = 40%; p = 0.91; withdrawing csDMARDs: RR 1.05, 95% CI 0.82–1.33; I^2 = 57%; p = 0.72. Withdrawing tsDMARDs (**c**) reduced the risk of any adverse events (from one study): RR 0.23, 95% CI 0.07–0.72.

Figure S33

a

Study or Subgroup	DMARDs withdrawal Events	DMARDs withdrawal Total	Standard DMARDs Events	Standard DMARDs Total	Weight	Risk Ratio M-H, Fixed, 95% CI	Year
Kjorholt 2025	10	47	5	45	5.5%	1.91 [0.71, 5.17]	2025
Ward 2025	8	69	4	33	5.9%	0.96 [0.31, 2.95]	2025
Kedra 2024	12	113	18	115	19.4%	0.68 [0.34, 1.34]	2024
Lillegraven 2023	3	47	2	45	0.0%	1.44 [0.25, 8.20]	2023
Emery 2023	3	50	3	50	3.3%	1.00 [0.21, 4.72]	2023
Curtis 2021	4	100	3	53	4.3%	0.71 [0.16, 3.04]	2021
Bouman 2017	50	115	15	57	21.8%	1.65 [1.02, 2.67]	2017
Weinblatt 2017	6	81	4	83	4.3%	1.54 [0.45, 5.25]	2017
Fautrel 2016	6	64	10	73	10.1%	0.68 [0.26, 1.78]	2016
van Vollenhoven 2016	0	23	1	23	1.6%	0.33 [0.01, 7.78]	2016
Chatzidionysiou 2016	3	15	1	16	1.1%	3.20 [0.37, 27.49]	2016
Moghadam 2016	34	531	7	286	9.9%	2.62 [1.17, 5.83]	2016
van Herwaarden 2015	30	121	7	59	0.0%	2.09 [0.98, 4.48]	2015
Smolen 2013	15	200	12	202	13.0%	1.26 [0.61, 2.63]	2013
**Total (95% CI)**		**1408**		**1036**	**100.0%**	**1.31 [1.02, 1.70]**	
Total events	151		83				

Heterogeneity: Chi² = 12.23, df = 11 (P = 0.35); I² = 10%
Test for overall effect: Z = 2.08 (P = 0.04)

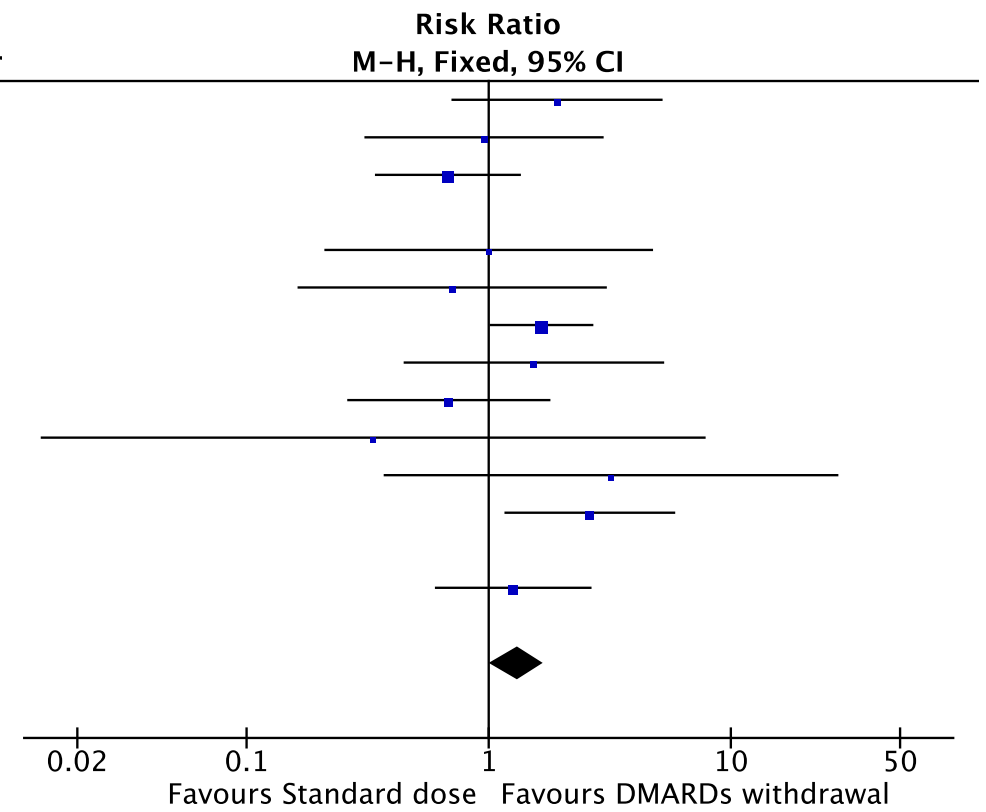

b

Study or Subgroup	DMARDs withdrawal Events	DMARDs withdrawal Total	Standard DMARDs Events	Standard DMARDs Total	Weight	Risk Ratio M-H, Random, 95% CI	Year
Asai 2025	5	43	0	39	10.7%	10.00 [0.57, 175.18]	2025
Kjorholt 2024	6	37	13	78	49.3%	0.97 [0.40, 2.36]	2024
Emery 2023	0	47	3	50	10.2%	0.15 [0.01, 2.86]	2023
Curtis 2021	4	99	3	53	29.8%	0.71 [0.17, 3.07]	2021
**Total (95% CI)**		**226**		**220**	**100.0%**	**0.94 [0.34, 2.57]**	
Total events	15		19				

Heterogeneity: Tau² = 0.33; Chi² = 4.31, df = 3 (P = 0.23); I² = 30%
Test for overall effect: Z = 0.12 (P = 0.91)

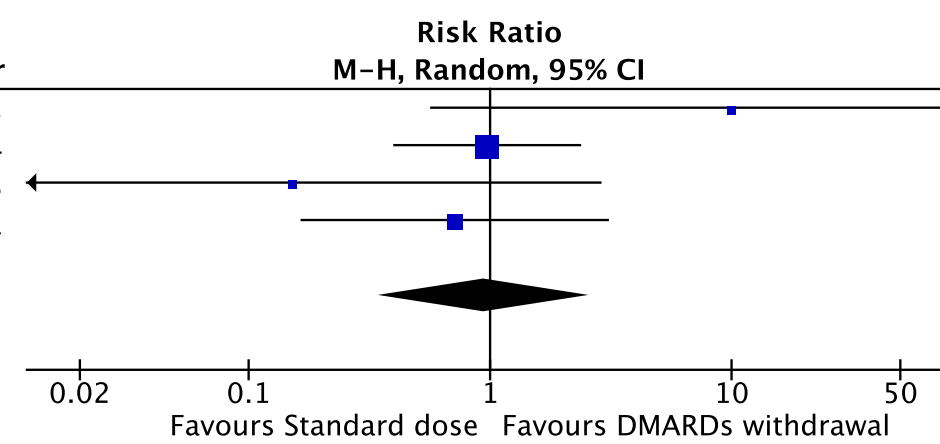

c

Study or Subgroup	DMARDs withdrawal Events	DMARDs withdrawal Total	Standard DMARDs Events	Standard DMARDs Total	Weight	Risk Ratio M-H, Fixed, 95% CI	Year
Wang 2023	0	39	1	41	100.0%	0.35 [0.01, 8.34]	2023
**Total (95% CI)**		**39**		**41**	**100.0%**	**0.35 [0.01, 8.34]**	
Total events	0		1				

Heterogeneity: Not applicable
Test for overall effect: Z = 0.65 (P = 0.52)

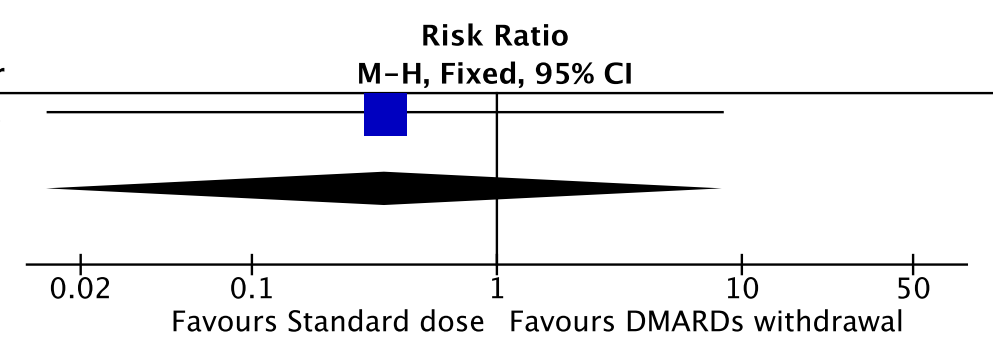

d

Study or Subgroup	DMARDs withdrawal Events	DMARDs withdrawal Total	Standard DMARDs Events	Standard DMARDs Total	Weight	Risk Ratio M-H, Fixed, 95% CI	Year
Tascilar 2021	13	96	10	93	100.0%	1.26 [0.58, 2.73]	2021
**Total (95% CI)**		**96**		**93**	**100.0%**	**1.26 [0.58, 2.73]**	
Total events	13		10				

Heterogeneity: Not applicable
Test for overall effect: Z = 0.58 (P = 0.56)

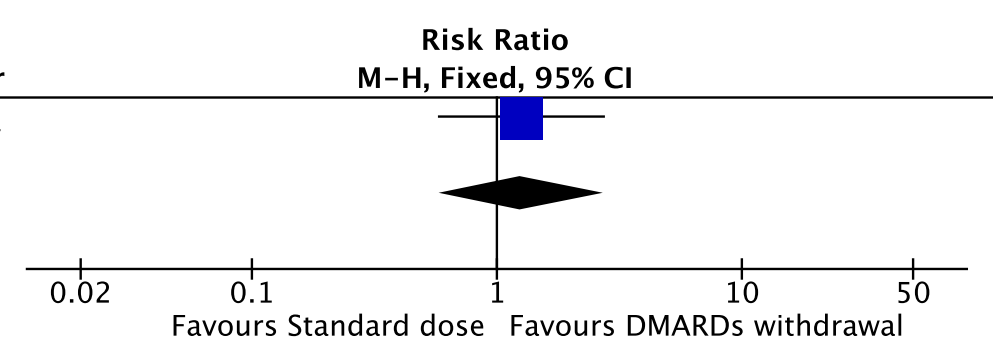

Figure S33. Subgroup analysis indicated that bDMARD withdrawal (**a**) was associated with increased risk of serious adverse events (RR 1.31, 95% CI 1.02–1.70; $p = 0.04$; $I^2 = 10\%$), whereas csDMARD withdrawal (**b**) (RR 0.94, 95% CI 0.34–2.57; $p = 0.91$), tsDMARD withdrawal (**c**) (RR 0.35, 95% CI 0.01–8.34; $p = 0.52$), or multiple DMARD withdrawal (**d**) (RR 1.26, 95% CI 0.58–2.73; $p = 0.56$) did not increase the risk of serious adverse events.

Figure S34

a

Study or Subgroup	DMARDs withdrawal Events	DMARDs withdrawal Total	Standard DMARDs Events	Standard DMARDs Total	Weight	Risk Ratio M-H, Fixed, 95% CI	Year
Chatzidionysiou 2016	3	15	1	16	100.0%	3.20 [0.37, 27.49]	2016
**Total (95% CI)**		**15**		**16**	**100.0%**	**3.20 [0.37, 27.49]**	
Total events	3		1				

Heterogeneity: Not applicable
Test for overall effect: Z = 1.06 (P = 0.29)

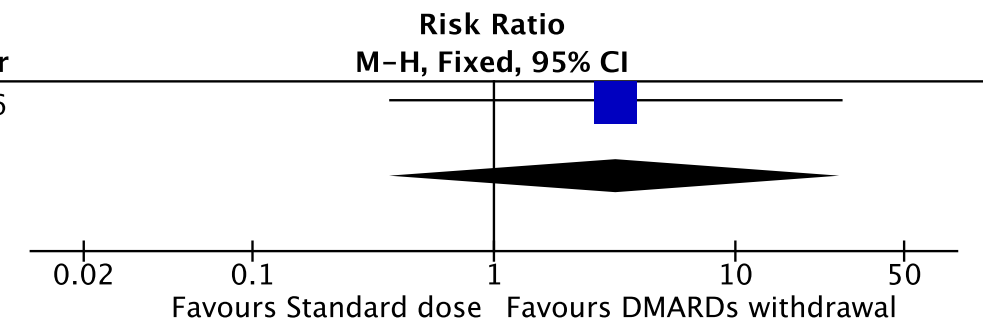

b

Study or Subgroup	DMARDs withdrawal Events	DMARDs withdrawal Total	Standard DMARDs Events	Standard DMARDs Total	Weight	Risk Ratio M-H, Fixed, 95% CI	Year
Ward 2025	8	69	4	33	9.1%	0.96 [0.31, 2.95]	2025
Lillegraven 2023	3	47	2	45	3.4%	1.44 [0.25, 8.20]	2023
Emery 2023	3	50	3	50	5.0%	1.00 [0.21, 4.72]	2023
Curtis 2021	4	100	3	53	6.6%	0.71 [0.16, 3.04]	2021
Weinblatt 2017	6	81	4	83	6.6%	1.54 [0.45, 5.25]	2017
Fautrel 2016	6	64	10	73	15.7%	0.68 [0.26, 1.78]	2016
van Vollenhoven 2016	0	23	1	23	2.5%	0.33 [0.01, 7.78]	2016
Moghadam 2016	34	531	7	286	15.3%	2.62 [1.17, 5.83]	2016
van Herwaarden 2015	30	121	7	59	15.8%	2.09 [0.98, 4.48]	2015
Smolen 2013	15	200	12	202	20.0%	1.26 [0.61, 2.63]	2013
**Total (95% CI)**		**1286**		**907**	**100.0%**	**1.43 [1.03, 1.98]**	
Total events	109		53				

Heterogeneity: $Chi^2 = 7.97$, df = 9 (P = 0.54); $I^2 = 0\%$
Test for overall effect: Z = 2.16 (P = 0.03)

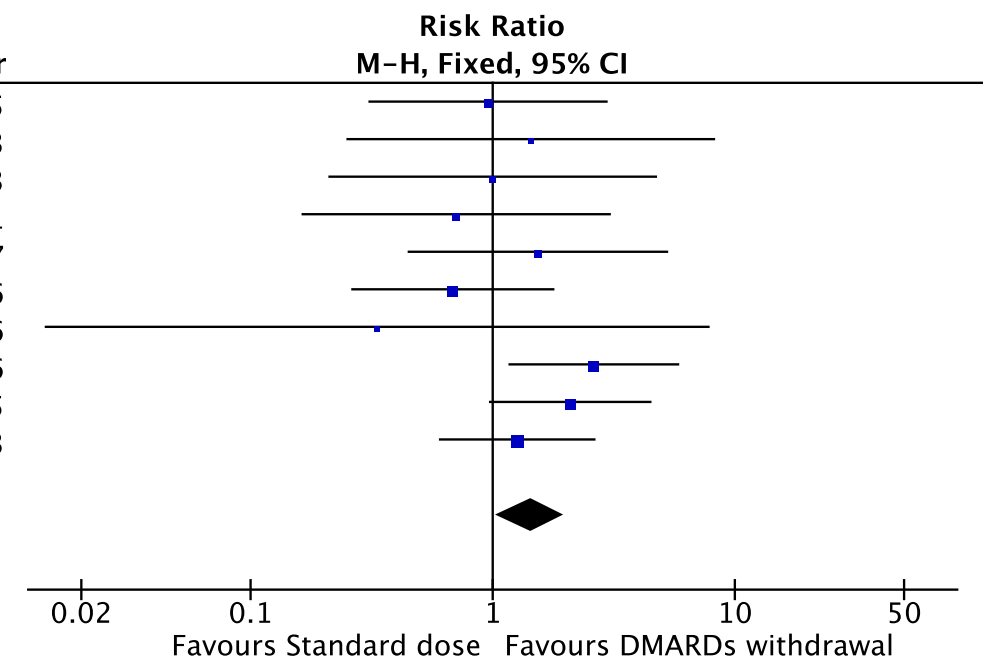

c

Study or Subgroup	DMARDs withdrawal Events	DMARDs withdrawal Total	Standard DMARDs Events	Standard DMARDs Total	Weight	Risk Ratio M-H, Fixed, 95% CI	Year
Kjorholt 2025	10	47	5	45	11.9%	1.91 [0.71, 5.17]	2025
Kedra 2024	12	113	18	115	41.5%	0.68 [0.34, 1.34]	2024
Bouman 2017	50	115	15	57	46.6%	1.65 [1.02, 2.67]	2017
**Total (95% CI)**		**275**		**217**	**100.0%**	**1.28 [0.89, 1.83]**	
Total events	72		38				

Heterogeneity: $Chi^2 = 5.03$, df = 2 (P = 0.08); $I^2 = 60\%$
Test for overall effect: Z = 1.35 (P = 0.18)

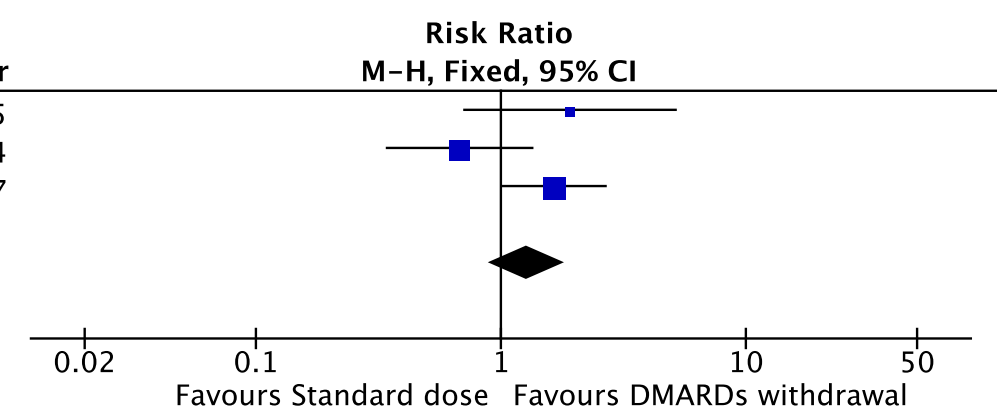

Figure S34. Subgroup analysis indicated that bDMARD withdrawal was associated with an increased risk of serious adverse events only during the intermediate-term follow-up (**b**) (RR 1.43, 95% CI 1.03–1.98; $p = 0.03$), but not in the short term (**a**) (RR 3.20, 95% CI 0.37–27.49; $p = 0.29$) or the long term (**c**) (1.28, 95% CI 0.89–1.83; $p = 0.18$).

Figure S35

a

Study or Subgroup	DMARDs withdrawal Events	DMARDs withdrawal Total	Standard DMARDs Events	Standard DMARDs Total	Weight	Risk Ratio M-H, Random, 95% CI	Year
Ward 2025	8	69	4	33	8.8%	0.96 [0.31, 2.95]	2025
Lillegraven 2023	3	47	2	45	3.7%	1.44 [0.25, 8.20]	2023
Emery 2023	3	50	3	50	4.6%	1.00 [0.21, 4.72]	2023
Curtis 2021	4	100	3	53	5.2%	0.71 [0.16, 3.04]	2021
Weinblatt 2017	6	81	4	83	7.4%	1.54 [0.45, 5.25]	2017
Fautrel 2016	6	64	10	73	12.2%	0.68 [0.26, 1.78]	2016
van Vollenhoven 2016	0	23	1	23	1.1%	0.33 [0.01, 7.78]	2016
Moghadam 2016	34	531	7	286	17.3%	2.62 [1.17, 5.83]	2016
van Herwaarden 2015	30	121	7	59	19.1%	2.09 [0.98, 4.48]	2015
Smolen 2013	15	200	12	202	20.6%	1.26 [0.61, 2.63]	2013
**Total (95% CI)**		**1286**		**907**	**100.0%**	**1.38 [0.99, 1.92]**	
Total events	109		53				

Heterogeneity: $Tau^2 = 0.00$; $Chi^2 = 7.97$, df = 9 (P = 0.54); $I^2 = 0\%$
Test for overall effect: Z = 1.88 (P = 0.06)

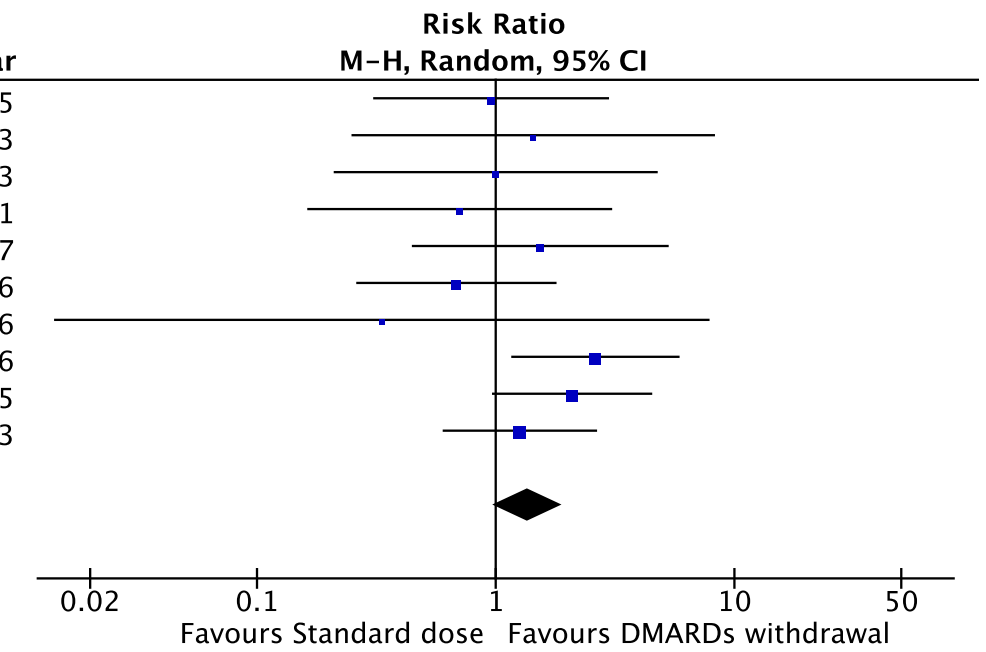

b

Study or Subgroup	DMARDs withdrawal Events	DMARDs withdrawal Total	Standard DMARDs Events	Standard DMARDs Total	Weight	Risk Ratio M-H, Fixed, 95% CI	Year
Ward 2025	8	69	4	33	10.8%	0.96 [0.31, 2.95]	2025
Lillegraven 2023	3	47	2	45	4.1%	1.44 [0.25, 8.20]	2023
Emery 2023	3	50	3	50	6.0%	1.00 [0.21, 4.72]	2023
Curtis 2021	4	100	3	53	7.8%	0.71 [0.16, 3.04]	2021
Weinblatt 2017	6	81	4	83	7.9%	1.54 [0.45, 5.25]	2017
Fautrel 2016	6	64	10	73	18.6%	0.68 [0.26, 1.78]	2016
van Vollenhoven 2016	0	23	1	23	3.0%	0.33 [0.01, 7.78]	2016
Moghadam 2016	34	531	7	286	18.1%	2.62 [1.17, 5.83]	2016
van Herwaarden 2015	30	121	7	59	0.0%	2.09 [0.98, 4.48]	2015
Smolen 2013	15	200	12	202	23.8%	1.26 [0.61, 2.63]	2013
**Total (95% CI)**		**1165**		**848**	**100.0%**	**1.31 [0.91, 1.88]**	
Total events	79		46				

Heterogeneity: $Chi^2 = 6.55$, df = 8 (P = 0.59); $I^2 = 0\%$
Test for overall effect: Z = 1.46 (P = 0.14)

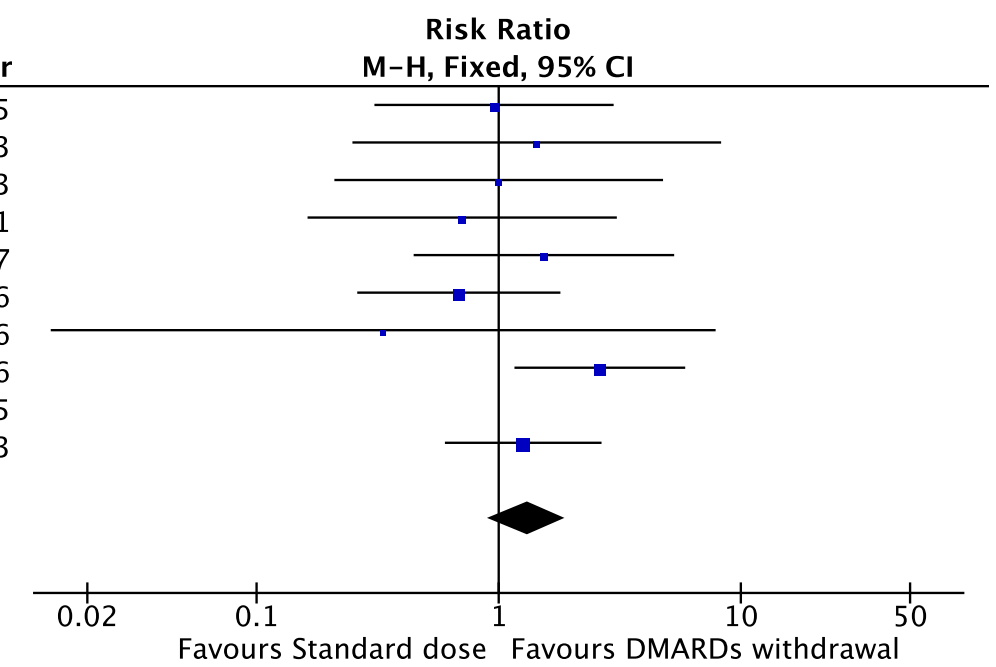

c

Study or Subgroup	DMARDs withdrawal Events	DMARDs withdrawal Total	Standard DMARDs Events	Standard DMARDs Total	Weight	Risk Ratio M-H, Fixed, 95% CI	Year
Ward 2025	8	69	4	33	10.7%	0.96 [0.31, 2.95]	2025
Lillegraven 2023	3	47	2	45	4.0%	1.44 [0.25, 8.20]	2023
Emery 2023	3	50	3	50	5.9%	1.00 [0.21, 4.72]	2023
Curtis 2021	4	100	3	53	7.8%	0.71 [0.16, 3.04]	2021
Weinblatt 2017	6	81	4	83	7.8%	1.54 [0.45, 5.25]	2017
Fautrel 2016	6	64	10	73	18.5%	0.68 [0.26, 1.78]	2016
van Vollenhoven 2016	0	23	1	23	3.0%	0.33 [0.01, 7.78]	2016
Moghadam 2016	34	531	7	286	0.0%	2.62 [1.17, 5.83]	2016
van Herwaarden 2015	30	121	7	59	18.6%	2.09 [0.98, 4.48]	2015
Smolen 2013	15	200	12	202	23.6%	1.26 [0.61, 2.63]	2013
**Total (95% CI)**		**755**		**621**	**100.0%**	**1.22 [0.85, 1.75]**	
Total events	75		46				

Heterogeneity: $Chi^2 = 4.94$, df = 8 (P = 0.76); $I^2 = 0\%$
Test for overall effect: Z = 1.08 (P = 0.28)

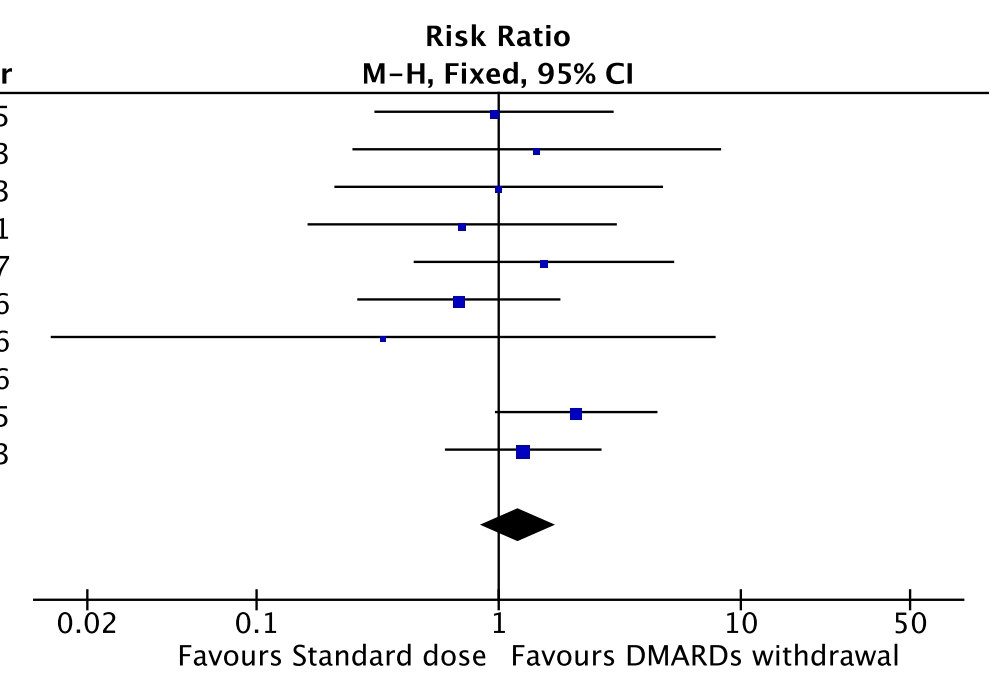

Figure S35. Sensitivity analysis did not confirm the robustness of the finding that bDMARD withdrawal was associated with increased risk of serious adverse events in the intermediate term. Sensitivity analysis using a random-effects model (**a**) attenuated statistical significance (RR 1.38, 95% CI 0.99–1.92; p = 0.06; $I^2 = 0\%$), and leave-one-out (LOO) analyses excluding either the DRESS trial (**b**) or the POET trial (**c**) also rendered the estimate non-significant.

Supplementary note 1

Searching strategies

PubMed
("antirheumatic agents"[MeSH Terms] OR "DMARD"[Title/Abstract] OR "Methotrexate"[Title/Abstract] OR "MTX"[Title/Abstract] OR "Leflunomide"[Title/Abstract] OR "Hydroxychloroquine"[Title/Abstract] OR "Sulfasalazine"[Title/Abstract] OR "tumor necrosis factor inhibitors"[MeSH Terms] OR "TNFi"[Title/Abstract] OR "Adalimumab"[Title/Abstract] OR "Etanercept"[Title/Abstract] OR "Infliximab"[Title/Abstract] OR "Golimumab"[Title/Abstract] OR "certolizumab pegol"[Title/Abstract] OR "janus kinase inhibitors"[MeSH Terms] OR "JAKi"[Title/Abstract] OR "Tofacitinib"[Title/Abstract] OR "Baricitinib"[Title/Abstract] OR "Upadacitinib"[Title/Abstract])
AND
("taper*"[Title/Abstract] OR "reduc*"[Title/Abstract] OR "de-escalat*"[Title/Abstract] OR "half dose"[Title/Abstract] OR "withdraw*"[Title/Abstract] OR "discontinu*"[Title/Abstract] OR "stop*"[Title/Abstract])
AND
("arthritis, rheumatoid"[MeSH Terms] OR "rheumatoid arthritis"[Title/Abstract])
AND
("remission induction"[MeSH Terms] OR "pathologic complete response"[MeSH Terms] OR "remission"[Title/Abstract] OR "sustained remission"[Title/Abstract] OR "clinical remission"[Title/Abstract] OR "low disease activity"[Title/Abstract] OR "LDA"[Title/Abstract])
AND
("randomized controlled trials as topic"[MeSH Terms] OR "randomized"[Title/Abstract] OR "randomised"[Title/Abstract] OR "trial"[Title/Abstract])

Embase including MEDLINE
('rheumatoid arthritis'/exp OR 'rheumatoid arthritis':ti,ab) AND ('remission'/exp OR remission:ti,ab OR 'sustained remission':ti,ab OR 'clinical remission':ti,ab OR 'low disease activity':ti,ab OR 'lda':ti,ab) AND (taper*:ti,ab OR discontinu*:ti,ab OR withdraw*:ti,ab OR 'de escalat*':ti,ab OR 'de escalat*' OR stop* OR reduc*) AND ('antirheumatic agent'/exp OR dmard:ti,ab OR methotrexate:ti,ab OR mtx:ti,ab OR leflunomide:ti,ab OR hydroxychloroquine:ti,ab OR sulfasalazine:ti,ab OR 'tumor necrosis factor inhibitor'/exp OR tnfi:ti,ab OR adalimumab:ti,ab OR etanercept:ti,ab OR infliximab:ti,ab OR golimumab:ti,ab OR 'certolizumab pegol':ti,ab OR 'janus kinase inhibitor'/exp OR jaki:ti,ab OR tofacitinib:ti,ab OR baricitinib:ti,ab OR upadacitinib:ti,ab) AND ('randomized controlled trial'/exp OR 'clinical trial'/exp) AND ('article'/it OR 'clinical trial'/it)

Cochrane library

**ID**	**Search**
#1	MeSH descriptor: [Arthritis, Rheumatoid] explode all trees
#2	("rheumatoid arthritis"):ti,ab,kw
#3	#1 OR #2
#4	MeSH descriptor: [Remission Induction] explode all trees
#5	remission:ti,ab,kw OR "sustained remission":ti,ab,kw OR "clinical remission":ti,ab,kw

OR "low disease activity":ti,ab,kw OR LDA:ti,ab,kw
#6 #4 OR #5
#7 taper*:ti,ab,kw OR reduc*:ti,ab,kw
OR discontinu*:ti,ab,kw OR withdraw*:ti,ab,kw
OR de-escalat*:ti,ab,kw OR stop*:ti,ab,kw
OR "half dose":ti,ab,kw
#8 MeSH descriptor: [Antirheumatic Agents] explode all trees
#9 DMARD:ti,ab,kw OR methotrexate:ti,ab,kw OR MTX:ti,ab,kw
OR leflunomide:ti,ab,kw OR hydroxychloroquine:ti,ab,kw
OR sulfasalazine:ti,ab,kw
#10 MeSH descriptor: [Tumor Necrosis Factor Inhibitors] explode all trees
#11 TNFi:ti,ab,kw OR adalimumab:ti,ab,kw OR etanercept:ti,ab,kw
OR infliximab:ti,ab,kw OR golimumab:ti,ab,kw
OR certolizumab:ti,ab,kw
#12 MeSH descriptor: [Janus Kinase Inhibitors] explode all trees
#13 JAKi:ti,ab,kw OR tofacitinib:ti,ab,kw
OR baricitinib:ti,ab,kw OR upadacitinib:ti,ab,kw
#14 #8 OR #9 OR #10 OR #11 OR #12 OR #13
#15 #3 AND #6 AND #7 AND #14
